# Bradykinin contributes to vasogenic edema in murine experimental cerebral malaria

**DOI:** 10.1172/JCI202285

**Published:** 2026-07-14

**Authors:** Alessandro de Sa Pinheiro, Douglas E. Teixeira, Rodrigo P. Silva-Aguiar, Young Jun Shim, Alona A. Merkulova, Sadiq Silbak, Yelenna Skomorovska-Prokvolit, David Midem, Sidney Ogolla, Bjoern B. Burckhardt, Tanja Gangnus, Julio Scharfstein, Celso Caruso-Neves, Owen J.T. McCarty, David Gailani, Michael Bader, Philip J. Rosenthal, Arlene E. Dent, Chris J. Janse, Keith R. McCrae, Ana Acacia de Sa Pinheiro, James W. Kazura, Alvin H. Schmaier

**Affiliations:** 1 Case Western Reserve University, Cleveland, Ohio, USA.; 2 Federal University of Rio de Janeiro, Rio de Janeiro, Brazil.; 3 Cleveland Clinic, Cleveland, Ohio, USA.; 4 Chulaimbo Sub-County Hospital, Kisumu, Kenya.; 5 Kenya Medical Research Institute, Kisumu, Kenya.; 6 Pharmacotherapy, Institute of Pharmaceutical and Medicinal Chemistry, University of Muenster, Muenster, Germany.; 7 Oregon Health & Science University, Portland Oregon, USA.; 8 Vanderbilt University Medical Center, Nashville, Tennessee, USA.; 9 Max-Delbrück-Center for Molecular Medicine in the Helmholtz Association (MDC), Charité – University Medicine, Berlin, Germany.; 10 DZHK (German Center for Cardiovascular Research), Partner Site Berlin, Berlin, Germany, Institute for Biology, University of Lübeck, Lübeck, Germany.; 11 University of California, San Francisco, San Francisco, California, USA.; 12 Leiden University Medical Center, Leiden, Netherlands.

**Keywords:** Hematology, Infectious disease, Vascular biology, Kinin

## Abstract

Cerebral malaria (CM) from *Plasmodium falciparum* is a major cause of death in African children. Since bradykinin (BK) is a mediator of vasogenic edema, we hypothesized that it contributes to the pathogenesis of CM in Kenyan children and *Plasmodium* *berghei* ANKA–infected (*PbA*-infected) C57BL/6J mice in experimental CM (ECM). Cleaved plasma high-molecular-weight kininogen (cHK) is a marker for BK release. 40% of children with central nervous system malaria had plasma cHK versus 18% of children with uncomplicated malaria. Wild-type *PbA*-infected mice with ECM had circulating cHK, elevated BK levels, and reduced HK and prekallikrein activity/antigen levels. HK-null (*Kng1^–/–^*), combined BK B1– and B2 receptor–null (*Bdkrb1^–/–^Bdkrb2^–/–^*), BK B2 receptor–null (*Bdkrb2^–/–^*), or BK B1 receptor–null (*Bdkrb1^–/–^*) mice were protected significantly from neurologic deterioration and brain edema compared with wild-type mice. *F12^–/–^* mice were not protected from neurological deterioration. Prekallikrein-null (*Klkb1^–/–^*), prolylcarboxypeptidase hypomorph (*Prcp^gt/gt^*), and brain endothelial cell conditional KO of PRCP (*Prcp^fl/fl^* Cre) mice with ECM had reduced neurologic deterioration and brain edema. Adjuvant plasma kallikrein inhibition combined with artesunate treatment in *PbA*-infected mice reversed neurologic deterioration and brain edema and significantly prolonged survival over artesunate alone. BK-induced vasogenic edema contributes to human and murine CM.

## Abstract 2

Bradykinin contributes to cerebral malaria and plasma kallkrein inhibitors to block its formation. Combining kallikrein inhibition with artesunate will reduce death from cerebral malaria.

## Graphical abstract

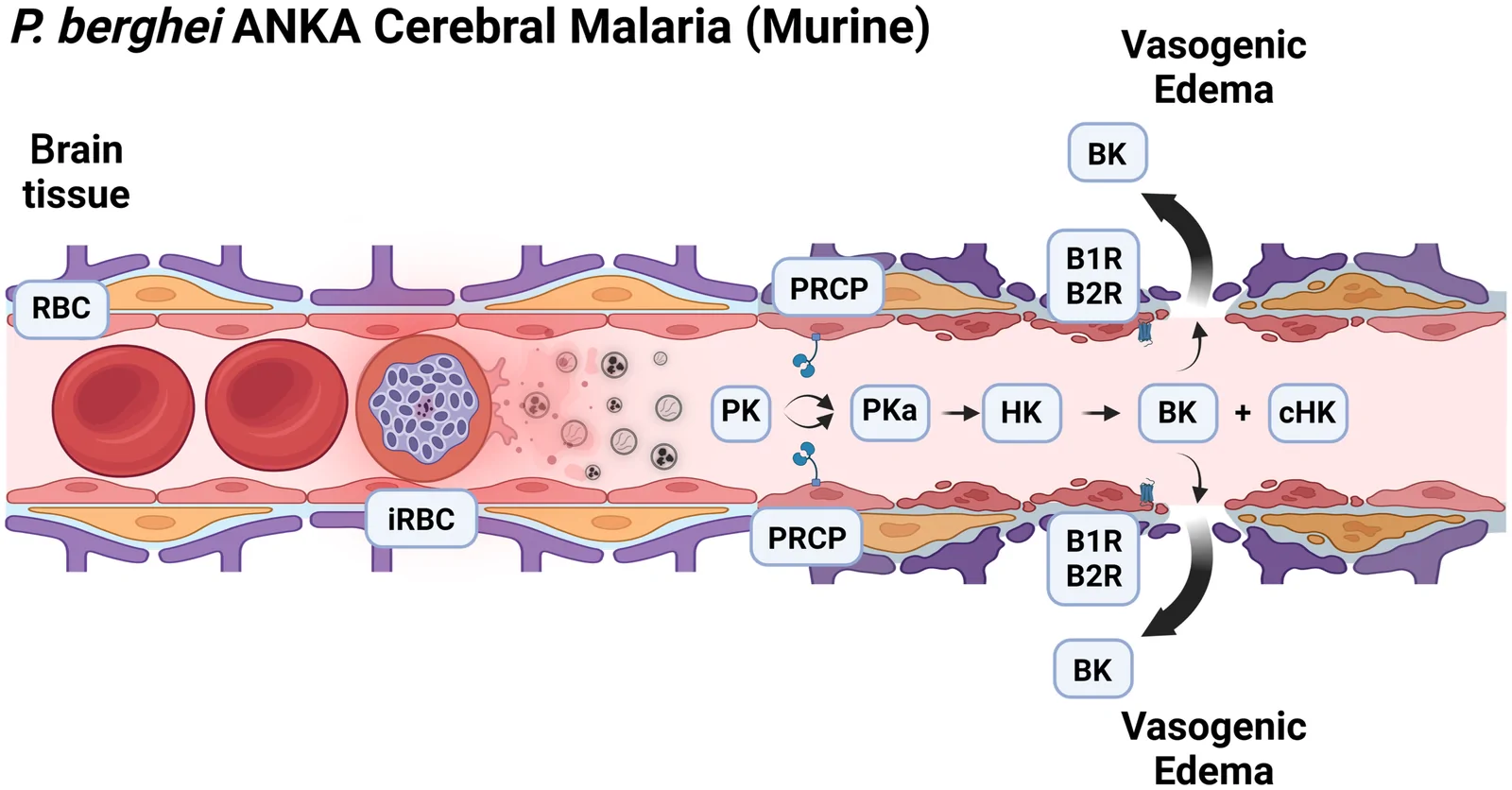

## Introduction

Malaria remains a major global health burden and a leading cause of death among children in sub-Saharan Africa. According to the World Health Organization World Malaria Report, there were an estimated 282 million malaria cases leading to 610,000 deaths in 2024 (1). Seventy-six percent of these deaths occurred in African children under 5 years old. Children with cerebral malaria (CM), a severe malaria phenotype, die despite artesunate therapy (2). Moreover, 30% of pediatric CM survivors experience postrecovery neurocognitive, motor, and learning deficits that have unclear significance to future growth and development (3–5).

Neuroimaging studies of pediatric CM in endemic sites by magnetic resonance show that the brain swelling is associated with vasogenic edema (breakdown of the blood brain barrier) and obstructed cerebral venous blood outflow (6, 7). In fatal CM, progressive brain swelling leads to brainstem dysfunction and uncal herniation. Features of pediatric CM pathogenesis include a high *Plasmodium falciparum* (*Pf*) biomass estimated by elevated *Pf* histidine–rich protein-2 (*Pf*HRP2) levels and increased circulating DNA accompanied by sequestration *of* *Pf*-infected erythrocytes in cerebral microvasculature, endothelial cell activation, and brain neuroinflammation with neutrophils and CD4^+^ and CD8^+^ T cells (8–13). However, the proximal mechanism(s) that initiates and mediates progressive brain swelling is not known. An investigation by Higgins et al. showed that administration of angiopoietin 1 in conjunction with antiparasite therapy increased survival of mice with experimental CM (ECM) (14). This ground-breaking study shows that improving the integrity of brain endothelial cell tight junctions with angiopoietin 1 reduces vasogenic edema and, along with artesunate therapy, improves murine ECM survival relative to artesunate alone (14). Adjuvant treatments to reduce the host response of brain vasogenic edema along with antiparasite therapy are a major unmet management need in CM treatment.

We previously reported that *Pf* culture medium coincubated with human brain microvascular endothelial cells induces disruption of intercellular junctions through bradykinin (BK) B2 and B1 receptors (15). Other investigations suggest that BK and related proteins participate in malarial disease (16). BK is a well-established mediator of increased vascular permeability and initiates edema formation in hereditary angioedema (HAE), implying that similar mechanisms might mediate endothelial barrier dysfunction and vasogenic edema in CM (17). Types I and II HAE are disorders characterized by acute bouts of localized tissue edema due to C1 inhibitor (C1INH) deficiency or defects. This disorder is initiated by activation of plasma prekallikrein (PK) to plasma kallikrein (PKa) that proteolyzes high-molecular-weight kininogen (HK), with release of BK leaving residual circulating plasma cleaved HK (cHK) (18–22). BK is a short-lived 9–amino acid peptide generated by proteolytic cleavage of HK and low-molecular-weight kininogen, both encoded by the single *KNG1* gene. Measurement of BK levels in vivo is challenging because the bioactive peptide is rapidly degraded and cleared from plasma with a half-life of approximately 34 seconds (23, 24). cHK has a half-life of approximately 10 hours and serves as an established biomarker of prior BK generation in plasma (20–22, 25).

In this study, we examined plasma samples from Kenyan children who presented with CM diagnosed at a rural hospital in western Kenya for detection of HK levels and cHK. The median (interquartile) Blantyre coma score was 2 (1–3, 26). The term central nervous system malaria (CNS-M), not CM, is used in describing these patient’s plasma HK and cHK levels since fundoscopic examination for malaria retinopathy was not performed and half the patients had Blantyre scores of 3 (27). Using the ECM model of *Plasmodium berghei* ANKA–infected (*PbA*-infected) C57BL/6J mice, we examined plasma for activation of the kallikrein/kinin systems and factor XII. Finally, we examined mouse genetic deletion models of the kallikrein/kinin systems and factor XII and the effect of inhibitors of selected proteins in this system on neurologic deterioration, vasogenic brain edema, and overall survival.

## Results

### Changes in plasma HK levels in Kenyan children with CNS-M, uncomplicated malaria, or acute febrile nonmalaria illness or healthy community individuals acting as controls.

Plasma from venous blood collected in heparin anticoagulant was isolated from Kenyan children with CNS-M, uncomplicated malaria (UM), and acute febrile nonmalaria illness (AFN) and healthy individuals in the community acting as controls (Healthy). Demographic and clinical characteristics of these children are presented in Table 1 (26). Children with CNS-M were younger than those with UM, had Blantyre coma scores of ≤3, and exhibited significantly higher plasma *Pf*HRP2 levels, consistent with increased *Pf* biomass compared with children with UM (Table 1).

**Figure 1:**
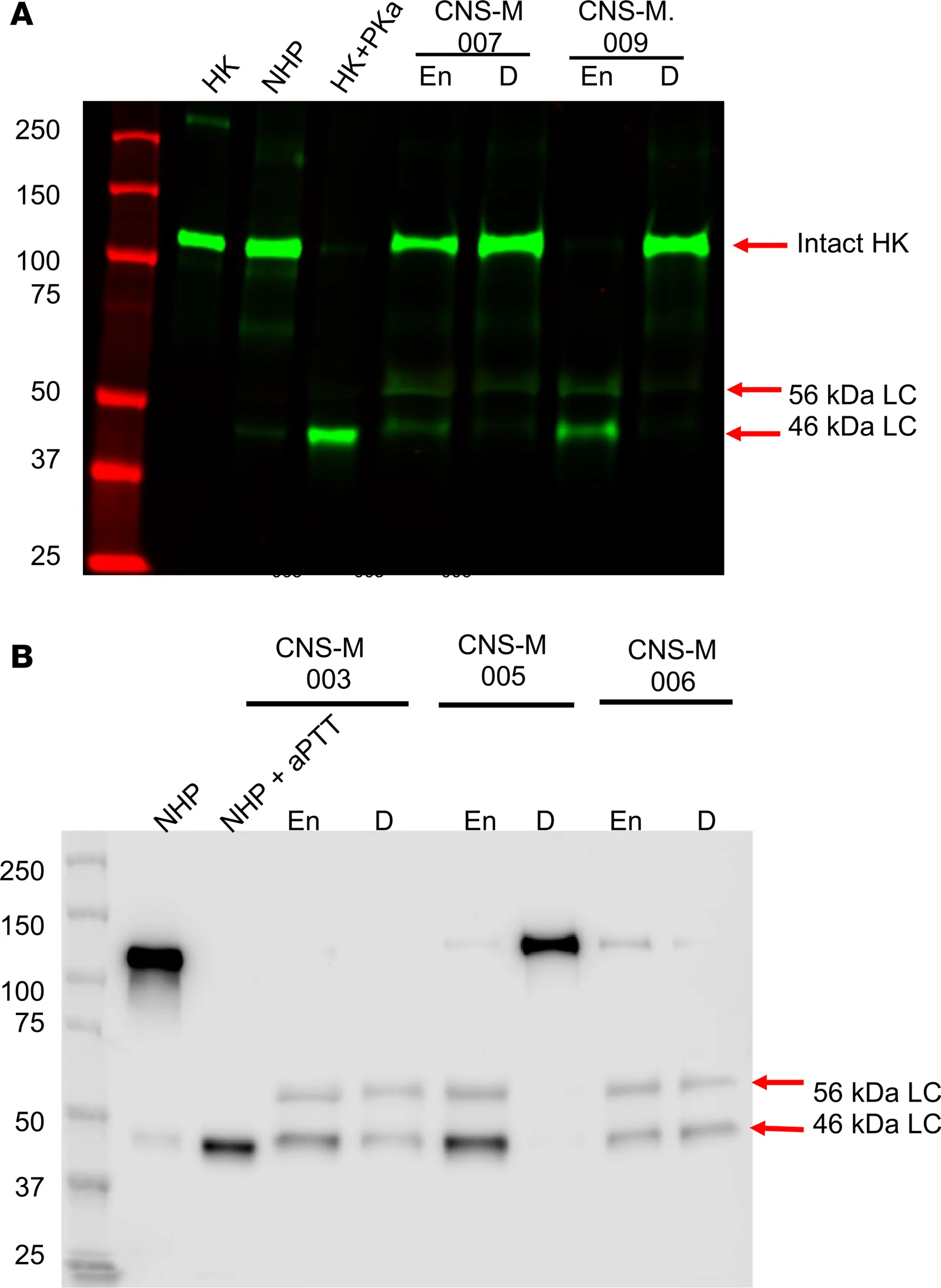
Immunoblot investigations of plasma high-molecular-weight kininogen on Kenyan children’s samples. (**A**) Immunoblot of 2 CNS-M patient plasma samples at hospital entry (En) and discharge (D) using a rabbit antibody against a human kininogen (HK) D5 peptide. The samples were reduced and added to the 7.5% SDS-PAGE. On the immunoblot, HK represents pure protein, NHP represents an immunoblot of pooled normal human plasma HK, and HK + PKa represents an immunoblot of a purified human HK cleaved by purified plasma kallikrein. Two arrows on the right side of the immunoblot point out the 56 and 46 kDa light chains of cleaved HK (cHK). A rabbit anti-D5 peptide antibody was used in the immunoblots (See Supplemental Tables 1 and 5) and developed using the Licor system. (**B**) Three additional CNS-M patient plasma samples at entry and discharge were studied. Normal human plasma was pooled from healthy North American donors. NHP + aPTT represents an immunoblot of pooled NHP activated with activated partial thromboplastin time (aPTT) reagent. The immunoblots were performed with the same anti-human HK D5 antibody as in **A**. It was developed with ECL. Note all plasmas (normal pooled or patient samples) in both blocks were added to the gel as 5 μL of a 1:10 dilution of plasma.

Plasma HK levels were measured by a competitive ELISA using a goat polyclonal antibody reared with isolated HK that detected HK light chain protein in both intact and cHK (Figure 1A, Table 2, and Supplemental Table 1; supplemental material available online with this article; https://doi.org/10.1172/JCI202285DS1). Patients with CNS-M at hospital entry (CNS-M-En) had HK levels that were significantly lower (*P* = 0.02) than those at hospital discharge (CNS-M-D) following artesunate treatment and clinical recovery (Supplemental Figure 1, A and B). All patients increased their plasma HK concentration from hospital entry to discharge (Supplemental Figure 1B). Patients with CNS-M-D and UM also had HK levels significantly higher than those with AFN (*P* = 0.003) and Healthy (*P* < 0.0001) children who were blood smear negative for Plasmodium parasites and PCR negative for Plasmodium 18s ribosomal RNA gene. Comparison between other groups was not significant (Table 2).

Since the differences in the quantity of total HK was not strikingly different among the patient samples, structural studies of HK by immunoblot were performed. These investigations showed that 8 of 20 CNS-M-En (40%) plasma samples collected before starting artesunate treatment had circulating cHK**,** a marker of prior BK released from HK (19–22). Four of these 8 children had Blantyre scores of ≤2.0; 4 children had Blantyre scores of 3. Using an anti-HK antibody reared to a peptide from human domain 5 of HK, pure intact HK and HK in normal human plasma on reduced SDS-PAGE appeared as a single approximately 120 kDa band (see the left side of Figure 1A). When the purified HK was treated with purified PKa, intact HK was mostly cleaved to a terminal 42–46 kDa light chain. These samples were used as controls for immunoblot studies of plasma from patients with CNS-M. In Figure 1A, for patient CNS-M 007, intact HK at 120 kDa was quantitatively less at hospital entry versus that at discharge, with intermediate 56 kDa and terminal 46 kDa bands of the cleaved light chain of HK. At the time of hospital discharge, plasma from patient CNS-M 007 had mostly intact HK with a faint 56 kDa light chain remaining. In Figure 1A, patient CNS-M 009 had no intact 120 kDa HK at study entry; only the two HK light chain fragments of cHK were detected at 56 and 46 kDa. At hospital discharge, plasma from this patient only contained intact 120 kDa HK. Three additional patients’ plasmas were characterized by immunoblot using the ECL system (Figure 1B). Patients CNS-M 003, 005, or 006 had little or no intact HK at entry. At discharge, only patient CNS-M 005 had no cHK and was discharged with intact HK (Figure 1B). In contrast to the patients with CNS-M, only 3 of 17 (18%) of patients with UM had circulating cHK at study entry. None of the plasma immunoblot samples from children with AFN or from the healthy uninfected children had cHK (data not shown). Thus, children with CNS-M and, less so, UM were associated with the presence of circulating cHK, a structure consistent with prior in vivo BK release (19–22). These observations suggested that BK could contribute to the vasogenic edema observed in human CM.

### Hematologic and coagulation parameters in C57BL/6J mice with ECM.

**Figure 2:**
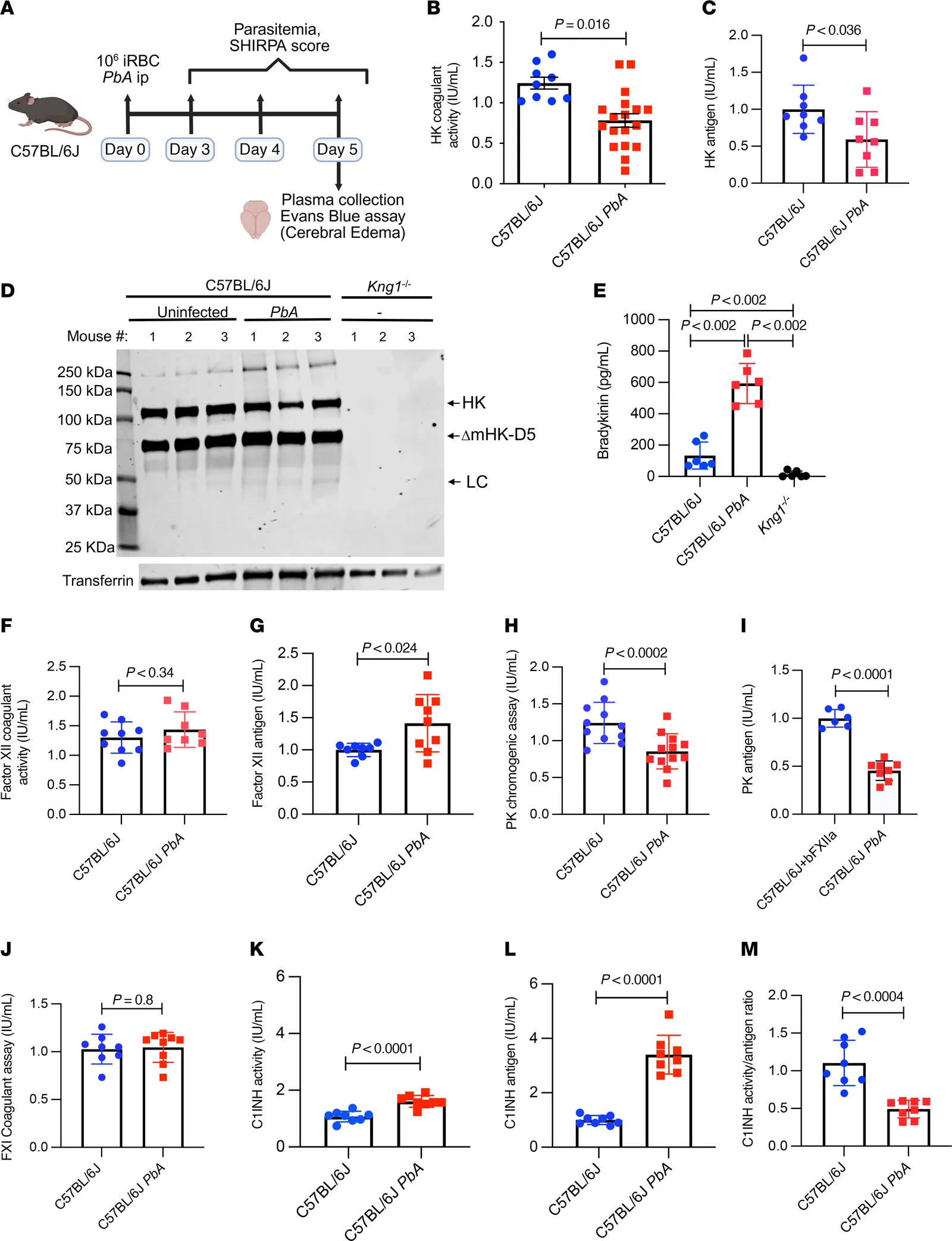
Characterization of plasma kallikrein-kinin proteins in murine ECM. (**A**) Schematic of *Plasmodium berghei* ANKA (*PbA*) infection ECM model (BioRender). (**B**) Plasma HK coagulant activity in uninfected (C57BL/6J) (*n* = 9) or *P*. *berghei* ANKA–infected (C57BL/6J *PbA*) (*n* = 17) mice. (**C**) Relative murine HK antigen by immunoblot densitometry of equal plasma volumes from uninfected (*n* = 8) or infected (*n* = 8) mice. (**D**) An immunoblot of murine HK with an antibody against its domain 6 with 3 reduced samples of uninfected murine plasma (Uninfected), *PbA*-infected (*PbA*) C57BL/6J mice, or total kininogen-deficient mice (*Kng1^–/–^*) collected on day 5. The immunoblot shows intact HK at ~120 kDa, ΔmHK-D5 ~ at 70 kDa (29), and LC — the cleaved 46 kDa light chain of HK. Transferin is the loading control. (**E**) Bradykinin measurement by LC/MS/MS in uninfected C57BL/6J mice (*n* = 6), PbA-infected C57BL/6J mice (*n* = 6), or total kininogen *Kng1^–/–^* mice (*n* = 6). The nonparametric data were analyzed by ANOVA with a correction for multiple comparisons using the Dunn’s test. (**F**) FXII coagulant activity from uninfected (*n* = 9) and *PbA*-infected (*n* = 8) C57BL/6J mouse samples. (**G**) The relative amount of murine FXII antigen by immunoblot densitometry of equal plasma volumes from uninfected (*n* = 8) and *PbA*-infected C57BL/6J mice (*n* = 9). (**H**) Plasma prekallikrein (PK) chromogenic activity assays in uninfected C57BL/6J (*n* = 11) and *PbA*-infected C57BL/6J (*n* = 12) mice. (**I**) Relative murine PK antigen by immunoblot densitometry of equal plasma volumes from uninfected (*n* = 6) and *PbA*-infected (*n* = 8) C57BL/6J mice. (**J**) FXI coagulant activity in uninfected (*n* = 8) and *PbA*-infected (*n* = 9) C57BL/6J mice. (**K**) C1INH functional activity in uninfected (*n* = 8) and *PbA*-infected (*n* = 8) mice. (**L**) C1INH antigen determined by immunoblot densitometry with 8 samples in each group. (**M**) Ratio of C1INH activity to antigen in the 8 samples from each group. All values represent mean ± SD. For HK, PK, FXII, FXI, and C1INH assays, all values were normalized to pooled normal mouse plasma and are expressed as IU/mL, which is the amount of that protein in 1 mL plasma. Comparisons between uninfected and infected mouse samples were performed by unpaired 2-tailed *t* test. *P* < 0.05 was considered significant.

Since blood samples from Kenyan children were collected in heparin, functional assays of the kallikrein/kinin system could not reliably be performed. We therefore established the ECM model of *PbA*-infected C57BL/6J mice to evaluate plasma hematologic and coagulation parameters of malaria pathogenesis. *PbA* murine malaria was used because *Pf* only infects homo sapiens. After i.p. inoculation with 10^6^ *PbA*-infected red blood cells (iRBCs) (Figure 2A), murine blood was collected at day 5 after infection at the time of onset of objective neurologic deterioration measured by the SHIRPA score (28). Relative to uninfected control mice, *PbA*-infected mice had significantly lower (*P* < 0.0001) white blood cell counts, lymphocyte counts, hematocrits, RBC mean corpuscular volume, and platelet counts (Supplemental Figure 2, A–C, and Supplemental Table 2). The mean corpuscular hemoglobin concentration in the *PbA*-infected mice was significantly higher than that of uninfected mice (Supplemental Figure 2A). The mean activated partial thromboplastin time (*P* = 0.0005) and prothrombin time (PT) (*P* = 0.008) were prolonged in *PbA*-infected mice compared with uninfected group (Supplemental Figure 2D and Supplemental Table 2). There was a trimodal distribution of the PT (Supplemental Figure 2D) that included a cluster of 5 mice with markedly prolonged PTs, a group with moderately prolonged PTs, and a third group with PTs comparable to uninfected mice. The clinical significance of these prothrombin time findings is presently not known since these 3 groups of mice had the same advanced, objective neurologic deficits on the SHIRPA score at the time of euthanasia on day 5 after infection.

We next examined if *PbA*-infected mice had changes in the levels of proteins of the PKa/kinin system and factor XII. Plasma HK levels in *PbA*-infected mice (0.78 ± 0.36 IU/mL coagulant activity and 0.62 ± 0.21 IU/mL HK antigen) were lower than values in uninfected mice (1.2 ± 0.21 IU/mL and 0.99 ± 0.15 IU/mL, *P* = 0.016 and *P* = 0.036, respectively) (Figure 2, B and C, and Supplemental Figure 3). An immunoblot of plasma samples using an antibody against murine HK domain 6 (D6) revealed 2 bands, one at approximately 120 kDa and a second at 100 kDa (ΔmHK-D5) detected in plasma from both uninfected and infected mice (Figure 2D). This latter form of murine HK had been previously described and corresponds to HK lacking domain 5 (D5) (29). Bands of the 46 kDa light chain of murine HK were seen in plasma from infected mice (Figure 2D). No bands were observed in HK-deficient plasma (*Kng1^–/–^*) mice (Figure 2D). These observations were confirmed with a second antibody targeting murine D6 in a different gel system (Supplemental Figure 4). Thus, mice with ECM had the same cHK phenotype observed in plasma from children with CNS-M or UM due to *Pf* infection. Further studies measured plasma BK levels that were significantly different by ANOVA with a Dunn’s test (*P* <0.0001). Plasma from wild-type C57BL/6J mice had a mean BK level of 134 ± 86 (mean ± SD) pg/mL (Figure 2E), while *PbA*-infected mice exhibited elevated BK levels of 593 ± 128 (mean ± SD) pg/mL (*P* < 0.002 vs. uninfected mice). In contrast, *Kng1^–/–^* plasma, total HK-deficient murine plasma, contained only 15 ± 19 (SD) pg/mL, a value below the lower limit of the standard curve of BK values in normal murine plasma.

Investigations next showed that individual murine FXII activity, although elevated compared with a pool of normal mouse plasma, was not different between uninfected and *PbA*-infected mice (Figure 2F). FXII antigen levels were increased not decreased in *PbA* infection (Figure 2G and Supplemental Figure 5). In contrast, plasma PK activity and antigen levels in *PbA*-infected mice were significantly lower (*P* = 0.0002 and *P* = 0.0001, respectively) than uninfected mice (Figure 2, H and I; Supplemental Figure 6; and Supplemental Table 3). Factor XI coagulant activity in plasma was similar between uninfected and *PbA*-infected mice (Figure 2J). Finally, plasma C1INH activity was 60% higher in *PbA*-infected versus uninfected mice (Figure 2K). Notably, infected mice had C1INH antigen levels 3-fold higher than uninfected mice and 2-fold higher than their activity values (Figure 2L and Supplemental Figure 7). Infected mice had a C1INH activity–to-antigen ratio of 0.5 (Figure 2M). These data indicated that *PbA*-infected mice had circulating C1INH that was 50% functional relative to uninfected mice. Combined, these results show that murine ECM is associated with HK cleavage, BK generation, and kallikrein activation.

### Role of HK and the BK receptors in ECM.

**Figure 3:**
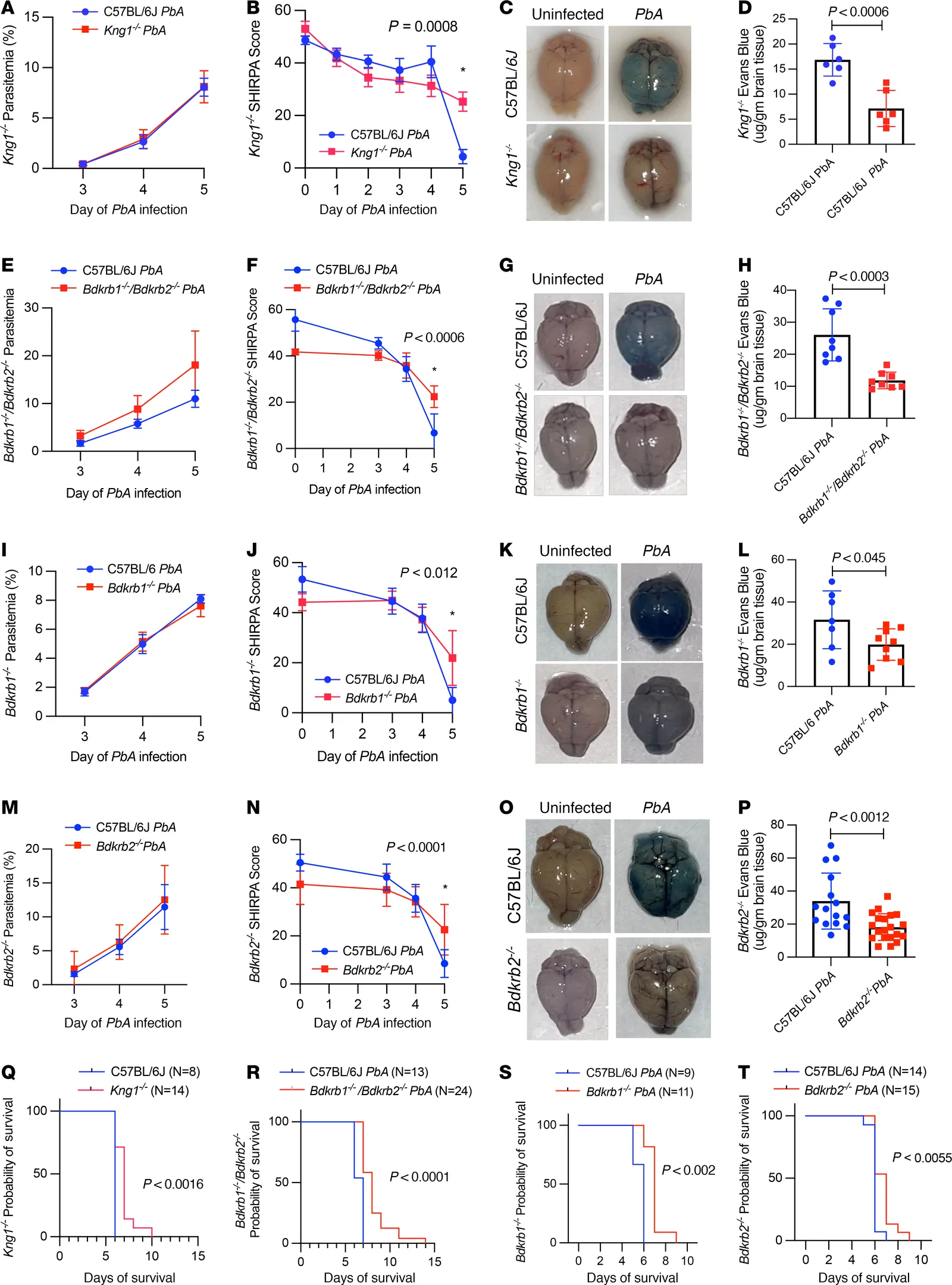
Characterization of *P*. *berghei* ANKA infection in HK- and bradykinin receptor–deleted mice. (**A**) Percentage parasitemia of infected wild-type C57BL/6J (*n* = 6) vs. infected *Kng1^–/–^* mouse samples (*n* = 7). (**B**) The SHIRPA score (28) is plotted for each day 3–5 for infected *Kng1^–/–^* (*n* = 7) or C57BL/6J (*n* = 6) mice. (**C**) Representative brain images of wild-type versus *Kng1^–/–^* mice brains after Evans Blue injection without (Uninfected) or with *P*. *berghei* ANKA infection (PbA). (**D**) Quantification of Evans Blue dye extravasation in brain from infected wild-type C57BL/6J (*n* = 6) versus *Kng1^–/–^* mice (*n* = 6). Studies on PbA-infected combined *Bdkrb1^–/–^Bdkrb2^–/–^* mice (*n* = 8) and control C57BL/6J mice (*n* = 8) are shown. (**E**) Parasitemia, (**F**) SHIRPA score, (**G**) representative brain images, and (**H**) Evans blue staining. Characterization of mice infected with PbA in bradykinin 1 receptor–null mice (*Bdkrb1^–/–^*) and controls: (**I**) parasitemia (*n* = 4 in each group), (**J**) SHIRPA score (*n* = 4 in each group), (**K**) representative brain images, and (**L**) Evans blue dye staining (*n* = 7 wild-type C57BL/6J and *n* = 9 *Bdkrb1^–/–^* mice) (mean ± SD of 2 experiments). *Bdkrb2^–/–^* mice and controls are as follows: (**M**) parasitemia, (**N**) SHIRPA score, (**O**) representative brain image, and (**P**) Evans blue dye staining. These data were with *n* = 18 wild-type and *n* = 20 *Bdkrb2^–/–^* mice for the parasitemia and SHIRPA scoring and *n* = 16 wild-type and *n* = 21 *Bdkrb2^–/–^* mice for the Evans blue study. (**Q**) *Kng1^–/–^* mouse Kaplan-Meier survival plot of *PbA*-infected C57BL/6J (*n* = 8) versus KO (*n* = 14) mice. (**R**) Combined *Bdrkb1^–/–-^Bdkrb2^–/–^* mouse Kaplan-Meier survival plot of *PbA*-infected C57BL/6J (*n* = 13) vs. combined KO (*n* = 24) mice. (**S**) *Bdkrb1^–/–^* mouse survival on a Kaplan-Meier plot of *PbA*-infected C57BL/6J (*n* = 9) vs. KO (*n* = 11) mice. (**T**) *Bdkrb2^–/–^* mouse Kaplan-Meier survival plot showed *PbA*-infected C57BL/6J (*n* = 14) vs. KO (*n* = 15) mice. Each graph represents the mean ± SD of 2 or 4 independent experiments. Comparisons between groups on day 5 of the SHIRPA scoring and Evans blue uptake were performed by unpaired 2-tailed *t* test. *P* < 0.05 was considered significant. Differences in the Kaplan-Meier plots survival curves were determined by Mantel-Cox and Gehan-Breslow-Wilcoxon tests performed independently.

Utilizing C57BL/6J mice with deletions in each gene encoding proteins of the kallikrein/kinin system and factor XII, we examined whether these animal models alone were associated with protection from *PbA*-induced neurologic deterioration, measured by the SHIRPA score and blood-brain barrier integrity measured by brain extravasation of Evans Blue dye and increased overall survival. The SHIRPA score is a detailed 5-point neurologic assessment of the mice that includes transfer arousal, locomotor activity, tail elevation, wire maneuvers, and contact righting reflex (28). Total *Kng1^–/–^* mice had a progressive increase in the level of parasitemia (approximately 8% at day 5 after infection) like that of wild-type mice (Figure 3A). Neurologic assessment at day 5 by SHIRPA score indicated that *Kng1*^–/–^ mice were protected against deterioration relative to wild-type mice (*P* = 0.0008) (Figure 3B and Supplemental Video 1) and had a significant reduction (*P* < 0.0006) in Evans Blue dye uptake into the brain (Figure 3D). In a representative image of a brain of a *PbA*-infected *Kng1^–/–^* mouse, the degree of Evans blue dye uptake was much less than that of a *PbA*-infected wild-type mouse (Figure 3C). Furthermore, *Kng1^–/–^* mice had increased survival, with a mean of 7 days. One animal lived for 10 days compared with infected C57BL/6J wild-type mice that all died by day 6 (Figure 3Q) (Supplemental Table 4). These studies showed that eliminating the source of BK alone without treating the infection delayed the time of disease onset and increased survival.

Combined BK B1 and B2 receptor–null mice (*Bdkrb1^–/–^Bdkrb2^–/–^)* had a level of parasitemia at day 5 after infection higher (17%) than that of control wild-type mice (10%) (Figure 3E). However, the SHIRPA score (Figure 3F) and Evans Blue dye uptake (Figure 3, G and H) showed a significant reduction in neurologic deterioration (*P* < 0.0006) and brain swelling (*P* < 0.0003) relative to that of wild-type *PbA*-infected mice, respectively (Supplemental Video 1). *PbA*-infected C57BL/6J wild-type mice all died by day 7, but the double BK receptor–KO mice had a mean survival of 8 days, with 1 animal living for 14 days after infection (Figure 3R) (Supplemental Table 4).

Studies next examined murine deletion mutants from each of the 2 individual BK receptors. Parasitemia levels of control wild-type and BK B1 receptor–KO (*Bdkrb1^–/–^*) mice on day 5 were identical (Figure 3I). However, SHIRPA scores showed a reduction in neurologic deterioration (*P* < 0.012) and brain Evans Blue dye uptake (*P* = 0.045) in the KO mice (Figure 3, J and L). *Bdkrb1^–/–^* mice had increased mean survival of 7 days compared with 6 days for wild-type mice (Figure 3S and Supplemental Table 4). BK B2 receptor–KO (*Bdkrb2^–/–^*) mice with 10% parasitemia also had a significance reduction from neurologic deterioration (*P* < 0.0001) and Evans Blue dye uptake (*P* = 0.0009) (Figure 3, M–P). *Bdkrb2^–/–^* mice also had an overall mean survival of 7 days (Figure 3T and Supplemental Table 4).

### PKa is causal in ECM.

**Figure 4:**
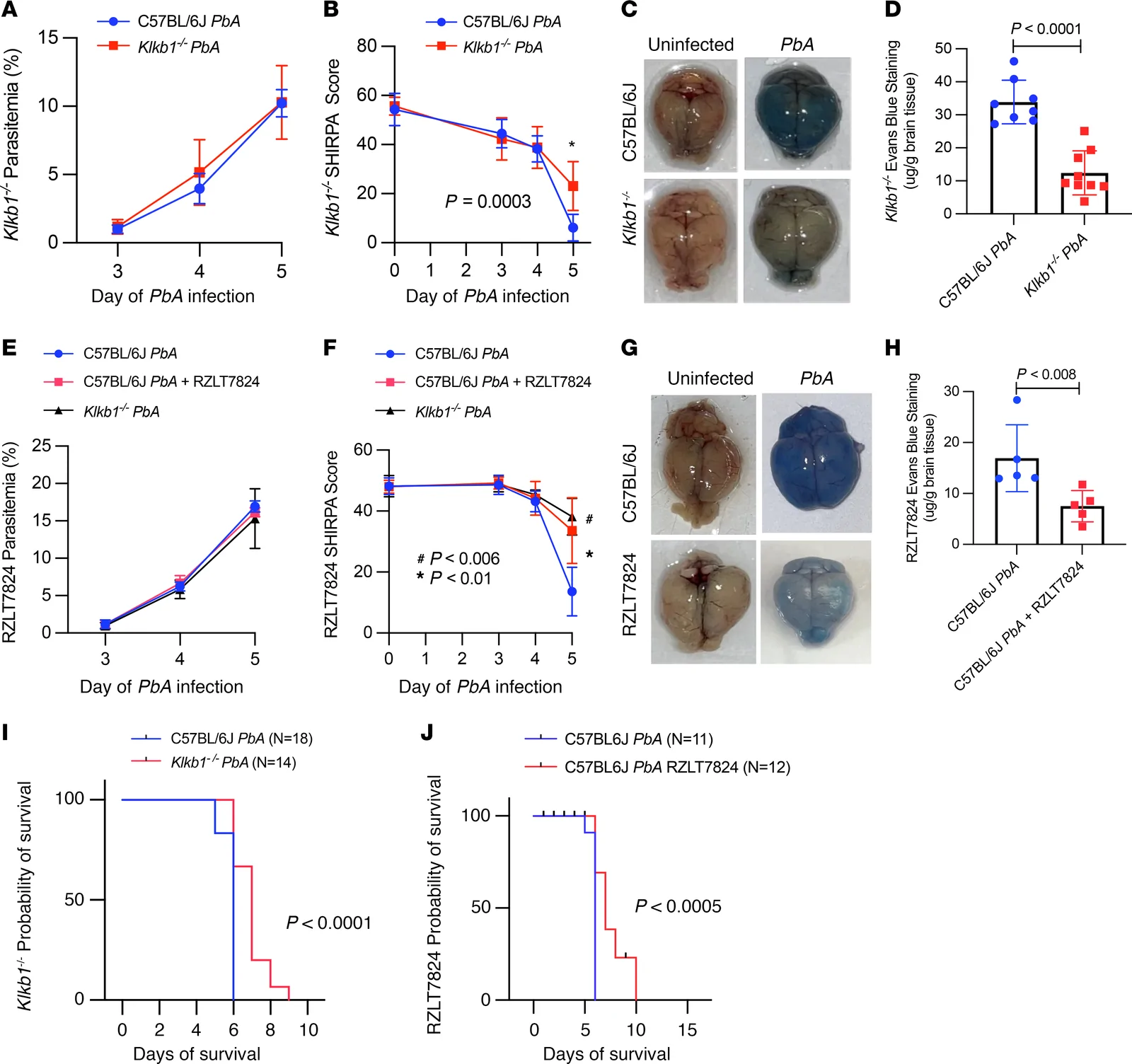
Characterization of *P*. *berghei* ANKA infection in prekallikrein-deficient mice and *PbA*-infected C57BL/6J mice given the plasma kallikrein inhibitor RZLT7824. Infected prekallikrein-deficient (*Klkb1*–/–) mice (*n* = 9) and their C57BL/6J controls (*n* = 8). (**A**) Parasitemia, (**B**) SHIRPA score, (**C**) representative brain images, and (**D**) Evans blue dye staining. Infected *P*. *berghei* ANKA–treated (*PbA*-treated) mice with RZLT7824 or controls next were investigated. *PbA*-infected C57BL/6J mice were treated with the plasma kallikrein inhibitor RZLT7824 starting on day 3. These studies are shown. (**E**) Parasitemia, (**F**) SHIRPA score, (**G**) representative brain images, and (**H**) Evans blue dye staining with *n* = 5 in each group of animals. The plasma kallikrein inhibitor RZLT7824 is compared against both infected C57BL/6J and *Klkb1^–/–^* mice in the SHIRPA scoring. (**I**) *Klkb1^–/–^* mouse Kaplan-Meier plot of *PbA*-infected C57BL/6J (*n* = 18) versus KO (*n* = 14) mice is shown. (**J**) A Kaplan-Meier plot of *PbA*-infected C57BL/6J (*n* = 11) mice versus C57BL/6J mice treated with RZLT7824 (*n* = 12). In each graph the combined data represent mean ± SD of 2 independent experiments with 2 or 3 mice in each experimental condition. Comparisons between groups on day 5 of the SHIRPA scoring and Evans blue uptake were performed by unpaired 2-tailed *t* test. *P* < 0.05. Differences in the Kaplan-Meier plots survival curves were determined by Mantel-Cox and Gehan-Breslow-Wilcoxon tests performed independently.

PK-null (*Klkb1^–/–^*) mice were investigated since PKa is the major enzyme to liberate BK from HK in human plasma. Parasitemia levels in *Klkb1^–/–^* mice were like those of wild-type mice (Figure 4A). Notably, *Klkb1^–/–^* mice had a highly significant reduction in neurologic deterioration on the SHIRPA score (*P* = 0.0003) (Figure 4B) and brain Evans Blue dye uptake (*P* < 0.0001) (Figure 4, C and D) (Supplemental Video 2). *Klkb1^–/–^* mice also had a significantly increased survival (*P* < 0.0001) and a mean survival of 7 days, whereas all the wild-type mice died by day 6 (Figure 4I and Supplemental Table 4). These findings showed that PK elimination alone modified ECM pathogenesis.

As an independent approach to examine the role of PKa in ECM pathogenesis, we determined if its enzymatic inhibition was also protective against vasogenic edema. RZLT7824 is a small-molecule inhibitor of PKa with an IC_50_ of 13 nM for isolated human PKa and >3.0 mM for human FXIIa, FXIa, FXa, FIIa, or plasmin. It inhibited mouse plasma PKa with an IC_50_ of 5 nM. RZLT7824 was administered at 60 mg/kg i.p. every 12 hours starting on day 3 of the *PbA* infection. On day 5, treated mice that exhibited identical parasitemia (Figure 4E) demonstrated significant preservation of neurologic and motor function, as shown by the SHIRPA score (*P* < 0.01) (Figure 4F) and reduced Evans Blue dye accumulation in the brain (*P* < 0.008) (Figure 4, G and H). In contrast, untreated infected C57BL/6J mice manifested neurologic defects (Supplemental Video 2). RZLT7824-treated*^-^* mice also had a significantly increased (*P* < 0.0005) mean survival of 7 days, whereas all the wild-type mice died by day 6 (Figure 4J and Supplemental Table 4; review Supplemental Videos 4, 5, and 6).

### Studies on potential mechanism(s) of plasma PK activation in ECM.

Initial studies determined if the parasite cysteine protease berghepain-2 has a role in BK formation in ECM. Since HK is a substrate of the cysteine protease falcipain-2 (PF3D7 1115700) and BK is a cleavage product (30), the contribution of the *PbA* ortholog berghepain-2 (PBANKA_0932400) to murine ECM was examined. Normal C57BL/6J mice were infected with wild-type *PbA* (*PBANKA^+/+^*) or *PbA* that had the berghepain-2 gene genetically silenced (*PBANKA^–/–^*). The levels of parasitemia were similar (10%) in the 2 groups at day 5 (Supplemental Figure 8A). The absence of berghepain-2 expression in blood-stage parasites did not alter neurologic deterioration on SHIRPA scoring (Supplemental Figure 8B) and Evans Blue dye brain uptake on day 5 (Supplemental Figure 8D). Thus, berghepain-2 did not contribute to murine ECM pathogenesis.

In the contact activation system of blood coagulation, activated factor XII (FXIIa) activates PK to PKa. We therefore infected factor XII–null mice (*F12^–/–^*) and compared them to infected wild-type mice. Parasitemia levels in wild-type and *F12^–/–^* mice were similar (Supplemental Figure 8E). However, FXII deficiency did not protect infected mice from progressive neurologic deterioration (Supplemental Figure 8F). In the Evans Blue dye assay, there was a significant reduction in brain edema (*P* < 0.03) but not to the extent seen with *Kng1^–/–^*, *Bdkrb2^–/–^*, or *Klkb1^–/–^* mice (Supplemental Figure 8H and Supplemental Table 4). This result could be explained by the reduction in a boost to PK activation due to the absence of FXIIa in reciprocal contact activation. Murine FXII deficiency did confer an increase in survival in ECM (*P* < 0.0001). *F12^–/–^* mice had a mean survival of 7 days, 24 hours longer than that of wild-type mice (Supplemental Figure 8I and Supplemental Table 4).

### Prolylcarboxypeptidase is the PK activator in ECM.

**Figure 5:**
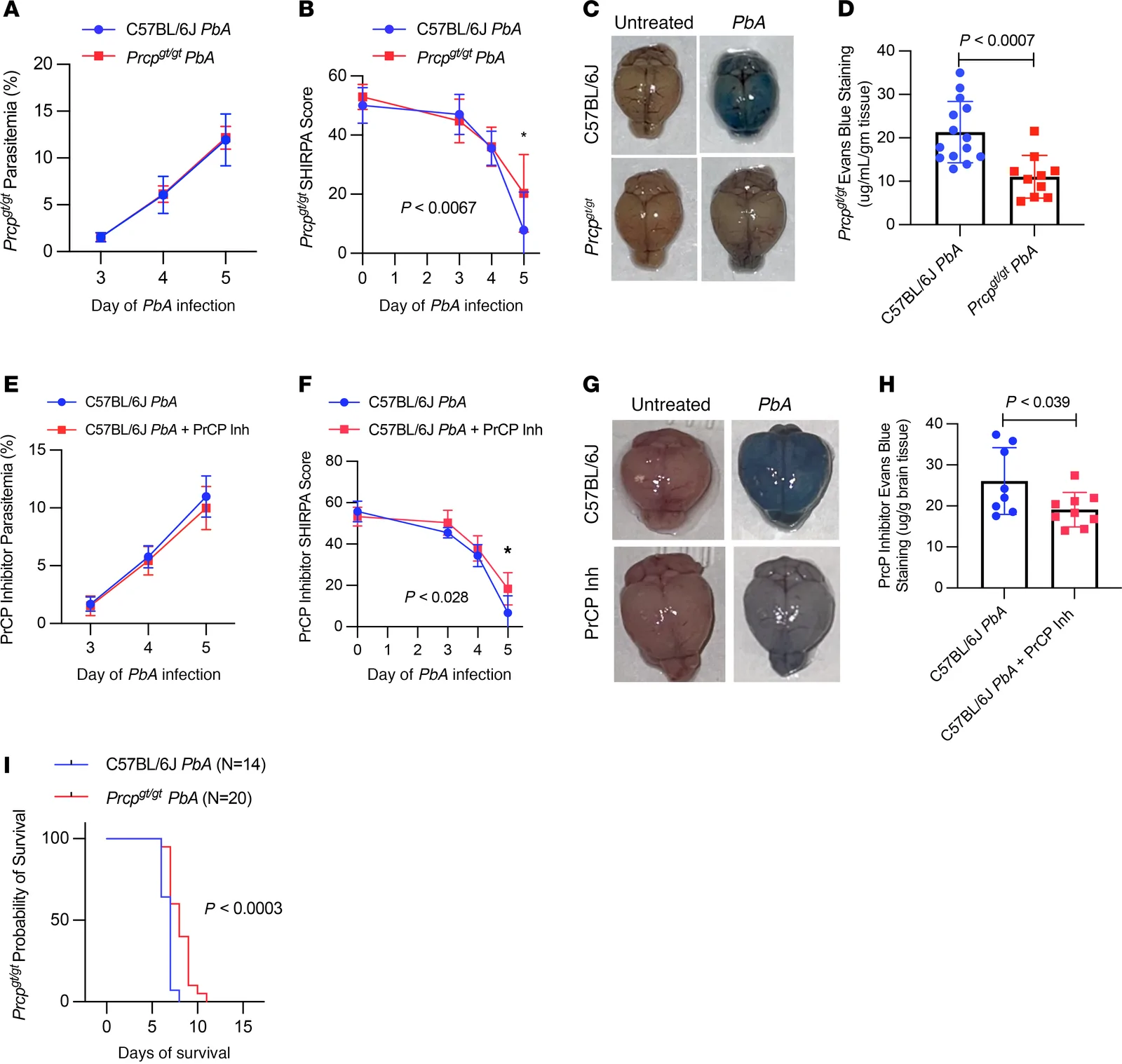
Characterization of *P*. *berghei* ANKA infection in prolylcarboxypeptidase gene trap and *P*. *berghei* ANKA–infected C57BL/6J mice given a prolylcarboxypeptidase inhibitor PrCP inhibitor. (**A**) Parasitemia, (**B**) SHIRPA score, (**C**) representative brain images, and (**D**) Evans Blue dye staining with 14 wild-type and 10 *Prcp^gt/gt^* mice. *P*. *berghei* ANKA–treated (*PbA*-treated) C57BL/6J mice were treated with the PrCP inhibitor starting on day 3. *PbA*-infected C57BL/6J mice without (*n* = 8) and with PrCP inhibitor (*n* = 9) were run simultaneously. (**E**) Parasitemia, (**F**) SHIRPA score, (**G**) representative brain images, and (**H**) Evans Blue dye staining were assessed. (**I**) *Prcp^gt/gt^* mouse survival: a Kaplan-Meier plot of *PbA*-infected C57BL/6J mice (*n* = 14) versus *Prcp^gt/gt^* (*n* = 10) mice. In each graph the combined data represent mean ± SD of 2 independent experiments with 5 or more mice in each experimental condition. Comparisons between groups on day 5 of the SHIRPA scoring and Evans blue uptake were performed by unpaired 2-tailed *t* test. *P* < 0.05. Differences in the Kaplan-Meier plots survival curves were determined by Mantel-Cox and Gehan-Breslow-Wilcoxon tests performed independently.

Plasma PK is activated on endothelial cell membranes by the serine protease prolylcarboxypeptidase (PRCP) (31). *Prcp*^gt/gt^ mice are gene trap hypomorphs with 3% PRCP mRNA (32, 33). *Prcp^gt/gt^* mice with 10% parasitemia (Figure 5A) had a significant reduction in neurologic deterioration by SHIRPA score (*P* < 0.0067) (Figure 5B) and brain Evans Blue dye uptake (*P* < 0.0007) (Figure 5, C and D) compared with wild-type controls. The mean survival of these animals after *PbA* infection 8.5 days (Figure 5I and Supplemental Table 4). To confirm a role for PRCP in ECM pathogenesis, wild-type C57BL/6J mice also were treated with an inhibitor of PRCP, named PrCP inhibitor, beginning at day 3 after *PbA* infection (34). Treated mice with a parasitemia of 10% showed a significant reduction in neurologic deterioration (*P* < 0.028) (Figure 5F) and brain Evans Blue dye uptake (*P* < 0.039) (Figure 5, G and H). These combined findings suggested that vessel wall PRCP, a PK activator, contributed to ECM pathogenesis along with HK, BK receptors and PK, independent of FXII.

**Figure 6:**
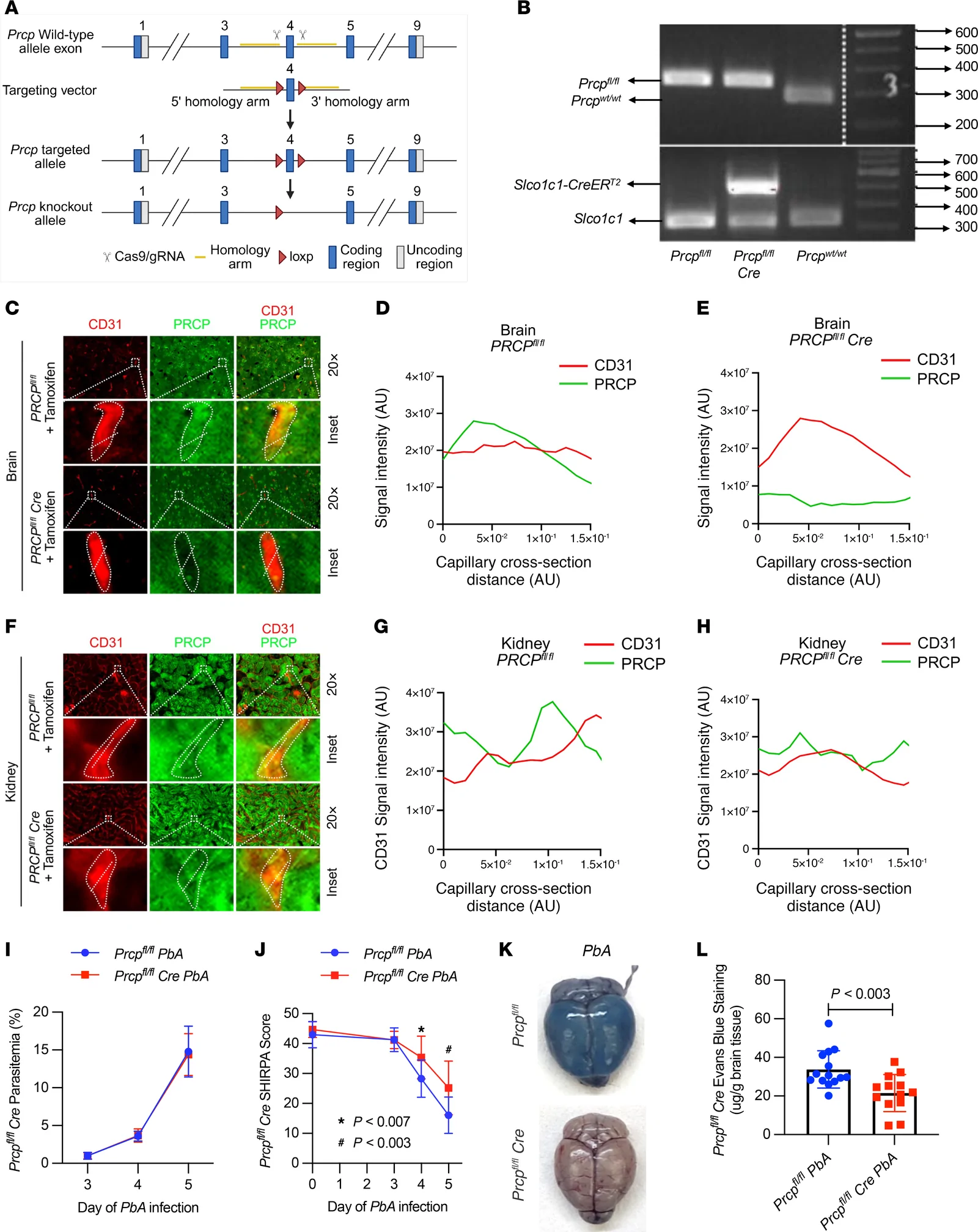
Studies with conditional KO mice of brain microvascular endothelial cell. (**A**) Targeting design for creation of *Prcp^fl/fl^* mice. The loxP sites were inserted around exon 4. Produced with BioRender. (**B**) Genetic characterization of the conditional KO of brain endothelial cell *Prcp*. Homozygous *Prcp^fl/fl^* mice were mated with hemizygous *Slco1c1-CreER^T2^* mice (35, 36) to create the conditional KO *Prcp^fl/fl^ ^Slco1c1-CreERT2^* mice. For presentation simplicity, the *Prcp^fl/fl^ ^Slco1c1-CreERT2^* mice are referred to as *Prcp^fl/fl^ Cre* mice. (**C**) Immunofluorescence staining of mouse brain microvascular tissue with CD31 and PRCP (original magnification, ×20). High-magnification (×100) images of brain microvessel boxed regions are shown, with white dotted lines encircling the enlarged microvessel. White dotted lines that run through the images show where vessel antigens were scanned for quantification. The scans are shown in the graphs to the right of the immunofluorescence (**D** and **E**). The immunofluorescence characterizes cerebral cortical tissue from tamoxifen-treated *Prcp^fl/fl^* and *Prcp^fl/fl^ Cre* mice. (**D**) A brain microvessel scan from the *Prcp^fl/fl^* mice. (**E**) A brain microvessel scan from the *Prcp^fl/fl^ Cre* mice. (**F**) Immunofluorescence staining of mouse renal microvascular tissue with CD31 and PRCP (original magnification, ×20). High-magnification (×100) images of magnified renal cortical microvessels are shown, with white dotted lines encircling the enlarged microvessel. White dotted lines that run through the images show where vessel antigens were scanned for quantification. The immunofluorescence characterizes renal cortical tissue from tamoxifen-treated *Prcp^fl/fl^* and *Prcp^fl/fl^ Cre* mice. (**G**) A renal cortical microvessel scan from the *Prcp^fl/fl^* mice. (**H**) A renal cortical microvessel scan from the *Prcp^fl/fl^ Cre* mice. The vessel wall was activated with tamoxifen treatment and *Cre* recombinase. *PbA* infection in *Prcp^fl/fl^* (*n* = 15) and *Prcp^fl/fl^ Cre* (*n* = 16) mice were compared for (**I**) parasitemia, (**J**) SHIRPA score, and (**K**) representative brain images. (**L**) Evans blue dye staining studies are shown with *n* = 14 *Prcp^fl/fl^* and *n* = 13 *Prcp^fl/fl^ Cre* mice. In each graph the combined data represent mean ± SD of 2 or 3 independent experiments with 5 or more mice in each experimental condition. Significant differences in the SHIRPA score on day 5 and Evans Blue uptake were determined by unpaired 2-tailed *t* test. *P* < 0.05.

*PbA* infection is associated with brain microvascular endothelium activation. Since PRCP had not been shown to activate PKa/kinin in vivo, we created brain microvascular endothelial cell–specific *Prcp* conditional KO mice. *Prcp*^fl/fl^ mice were created by flanking exon 4 with loxP sites (Figure 6A). *Slco1c1-CreER^T2^* mice express a tamoxifen-inducible *Cre* recombinase under control of a thyroxine transporter promoter in brain microvascular endothelium (35). This *Cre* mouse has been validated by several laboratories (36–38). Homozygous *Prcp^fl/fl^* mice were crossed with hemizygous *Slco1c1-CreER^T2^* mice. These mice (named *Prcp^fl/fl^ Cre*), which had the two separate genotyping features of being homozygously floxed (*Prcp^fl/fl^*) and containing the *Cre* recombinase gene (*Slco1c1-CreER^T2^*) (see Figure 6B), were selected for treatment with tamoxifen to induce recombination. Immunofluorescence studies showed that when tamoxifen-treated *Prcp^fl/fl^ Cre* mice and *Prcp^fl/fl^* mice, the experimental control, were compared, brain microvascular endothelial cell PRCP was removed from the *Cre*-containing mice (Figure 6C). As shown in the top left inset of Figure 6C, brain cortex tissue from tamoxifen-treated *Prcp^fl/fl^* mice showed strong red CD31 vessel antigen. That same vessel when outlined contained green PRCP antigen. When combined, the merged image had an orange color, showing the presence of both antigens in the vessel wall. Figure 6D shows a scan across the *Prcp^fl/fl^* mouse vessel, as indicated by the white dotted line in the images, showing the presence and overlap of both the CD31 (red) and PRCP (green) antigens. Brain cortex tissue from *Prcp^fl/fl^ Cre* mice (in the bottom left inset of Figure 6C) also had a strong red CD31 vessel antigen but, in contrast, no green PRCP antigen in the vessel. The absence of the PRCP antigen in the image appears as a black hole in the immunofluorescence. Merging with red vessel wall CD31 with no vessel wall green from PRCP did not dilute the red color. Furthermore, a scan of the antigen(s) in the graph in Figure 6E showed no green PRCP antigen overlapping the red CD31 vessel antigen. These combined data indicated that PRCP antigen was not present in the brain microvessels of the tamoxifen-treated *Prcp^fl/fl^ Cre* mice. These results contrasted with studies of kidney tissue taken from the same *Prcp^fl/fl^* and *Prcp^fl/fl^ Cre* mice after tamoxifen treatment (Figure 6F). Both mouse genotypes had overlapping CD31 and PRCP antigen in renal microvessels between renal proximal tubules (Figure 6, F, G, and H). In sum, these data confirmed the absence of PRCP in the *Prcp^fl/fl^ Cre* mice after tamoxifen treatment was specific for brain endothelium.

The conditional brain microvessel *Prcp*-KO mice were examined to see if they influenced *PbA* infection. After tamoxifen treatment, the *Prcp^fl/fl^* (control mice for this experiment) and *Prcp^fl/fl^* *Cre* mice have similar levels of parasitemia (Figure 6I). *Prcp^fl/fl^* *Cre* mice were significantly protected from neurologic deterioration, as determined by SHIRPA score at days 4 (*P* < 0.007) and 5 (*P* < 0.033), respectively, compared with the *Prcp^fl/fl^* mice (Figure 6J). Furthermore, there was a reduction of brain Evans Blue dye uptake (*P* < 0.003), indicating preservation of blood-brain barrier integrity in the *Prcp^fl/fl^* *Cre* mice (Figure 6, K and L and Supplemental Table 4). These combined data indicated that brain vessel wall PRCP had a significant role in the pathogenesis of ECM.

### The use of PKa inhibition as an adjuvant to artesunate in the treatment of ECM.

**Figure 7:**
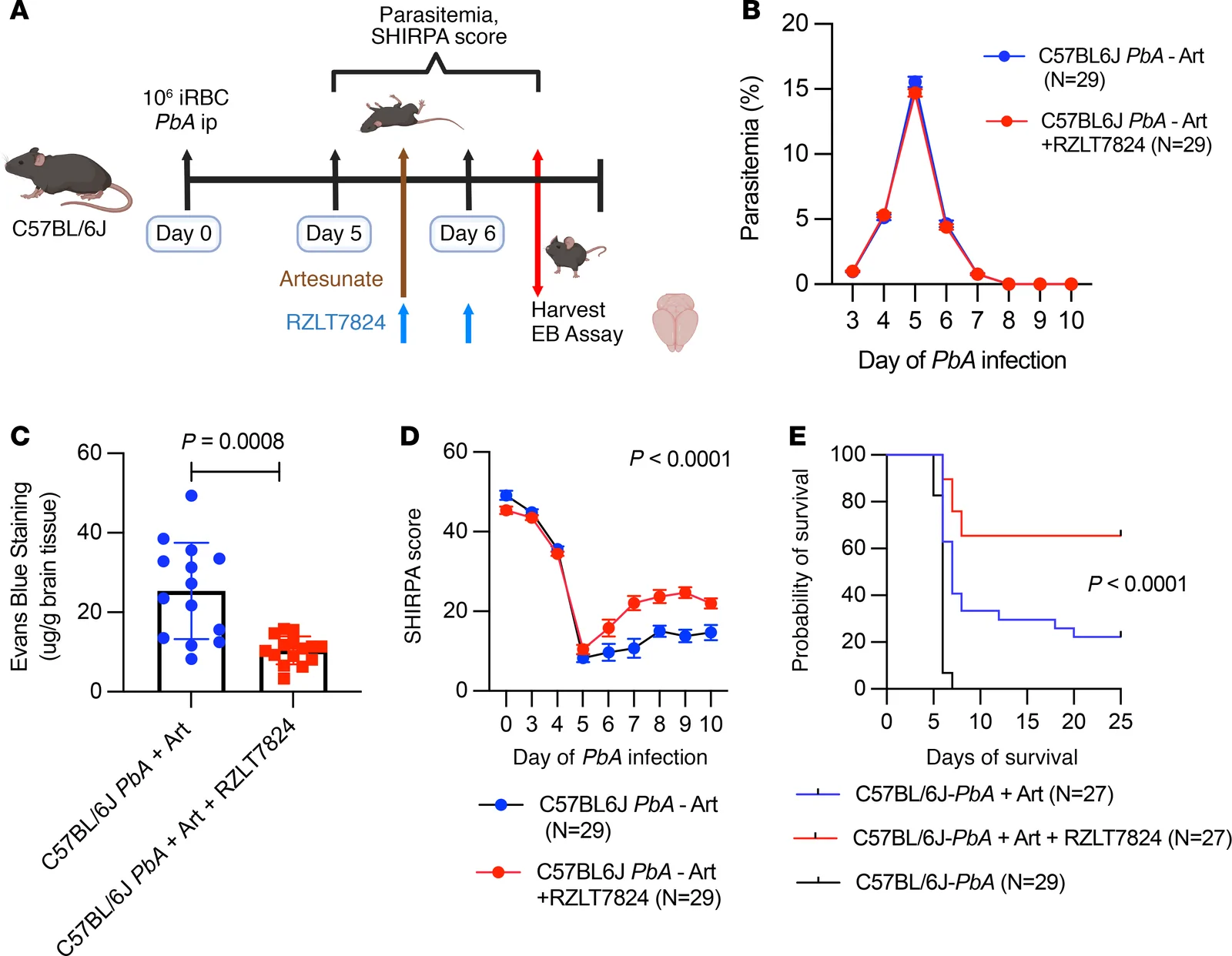
Characterization of RZLT7824 as an adjunct therapy to artesunate treatment in experimental cerebral malaria. (**A**) Treatment schema. On day 5, when C57BL/6J mice begin to show objective neurologic deterioration from *PbA* infection, 1 group of mice was given artesunate at 32 mg/kg i.p. once daily verses another group that was given artesunate on the same schedule and RZLT7824 at 80 mg/kg i.p. every 12 hours, both for 10 days. Produced with BioRender. (**B**) Parasitemia (*n* = 10 mice in each group). (**C**) Evans blue dye staining on day 6 with *n* = 14 mice treated with artesunate alone and *n* = 16 mice treated with artesunate and RZLT7824. (**D**) SHIRPA score (*n* = 29 mice in each group) after 10 days. (**E**) Kaplan-Meier survival plot with 27 infected wild-type mice treated with artesunate, 27 infected wild-type mice treated with artesunate and RZLT7824, and 29 infected wild-type mice alone. All animals in the survival studies were treated for 12 days, and the mice were monitored for 25 days total. In each graph the combined data represent mean ± SD of 3 independent experiments with 10 or more mice in each experimental condition. Significant differences in the SHIRPA score on days 5–10 and Evans Blue uptake on day 6 were determined by unpaired 2-tailed *t* test. *P* < 0.05. Differences in the Kaplan-Meier plots survival curves were determined by Mantel-Cox and Gehan-Breslow-Wilcoxon tests performed independently.

Using *PbA*-infected wild-type mice, we mimicked a situation that is commonly seen in hospitals in rural Africa when children present with coma and seizures with evidence of brain swelling and malaria retinopathy. We performed these studies with the PKa inhibitor RZLT7824 because (a) PK deficiency is a clinically benign condition, (b) *Klkb1^–/–^* mice are protected from thrombosis (39), and (c) several PKa inhibitors in different formulations are FDA approved to treat HAE without long-term consequences. Alternatively, PRCP is not an ideal target for these studies because its depletion produces hypertension, cardiac disease, and thrombosis (33, 40). The treatment protocol in Figure 7A shows that on day 5 after infection, when the animals showed objective signs of neurologic deterioration 4–12 hours into the day, one group was treated with the antiparasitic drug artesunate (32 mg/kg i.p.) daily and a second group was treated with artesunate plus RZLT7824 (80 mg/kg i.p.) every 12 hours. Murine parasitemia and SHIRPA score were evaluated daily through day 10. The two groups had similar parasitemia levels with a peak on day 5 before artesunate administration and approximately 100% decreased parasitemia 48 hours after treatment (Figure 7B). On day 6, the combined artesunate- and RZLT7824-treated animals had a significant reduction in brain Evans Blue dye uptake (*P* < 0.0008) (Figure 7C). Supplemental Video 3 shows surviving mice in both groups on day 6. When the animals’ behavior was characterized by the SHIRPA score (Figure 7D), the artesunate + RZLT7824 group had a significantly improved (*P* < 0.0001) SHIRPA score above 20 on late day 6. The SHIRPA score of the combined treated mice stayed above 20 through day 10. At no time did the SHIRPA score of the artesunate-treated only mice increase above 20. Figure 7E shows overall survival. In this experiment, mice were treated upon the onset of neurologic signs on day 5 until day 12, then followed for 25 days total. Untreated mice died by day 6. Mice treated with artesunate alone had only 28% survival at day 25. Alternatively, mice treated with combined artesunate and RZLT7824 had 68% survival at day 25. Adding the PKa inhibitor to treatment with antiparasite agent increased survival by 168%. These findings demonstrate that PKa inhibition provides significant therapeutic benefit when combined with artesunate by targeting host-mediated vascular dysfunction rather the parasite clearance.

## Discussion

MRI studies have shown that a major feature of pediatric CM is progressive brain swelling due to breakdown of the blood-brain barrier and vasogenic edema that can ultimately lead to brainstem herniation and death (7). We found that children with CNS-M and mice with ECM have cHK circulating in plasma with the presence of the 56 kDa and 46 kDa light chains of HK (Figure 1 and Figure 2D). This pattern of cleavage is an established marker of prior BK liberation, BK being a biologic peptide that induces vasogenic edema (19–24, 34). The observed HK cleavage, consistent with proteolysis by PKa or activated FXIIa indicates that the PKa/kinin system activation participates in the pathogenesis of CM (19–22).

The presence of circulating cHK is uncommon in humans and mice. In humans, cHK in plasma is characteristic of acute attacks of HAE due to C1INH deficiency or disfunction (20–22). Similarly, *Serping1^–/–^* mice that lack C1INH protein expression constitutively generate cHK (41). cHK is observed in patients with Alzheimer’s disease and cancer (42, 43). With respect to human CNS-M, only 40% of patients with CNS-M and 18% patients with UM had detectable cHK in plasma. Only 4 of the 8 children with plasma cHK had Blantyre scores of 2 or less. Thus, cHK is not a sensitive marker for pediatric CNS-M. Alternatively, all mice with ECM had cHK on day 5 when they had clinically objective neurologic deficits. The observation that some children with UM had cHK detectable in plasma suggest that circulating cHK is not specific to CM. However, these apparent differences are not necessarily due to an inconsistency between CM in children and mice. In ECM, plasma is collected when mice have advanced neurologic signs and a reduced SHIRPA score. In real-world clinical settings in rural Africa, parents bring their children to healthcare facilities at variable times after the onset of febrile illness — some at the first sign of sickness and others with advanced disease characterized by seizures and coma (44). CM may occur late during a *Pf* infection when all the conserving mechanisms to prevent BK accumulation are saturated by a high rate of production (45).

The vasogenic peptide implicated in this study as a mediator of CM is BK. cHK is detectable in plasma from children with CNS-M and mice with ECM. Moreover, mice with ECM have elevated BK levels. Furthermore, genetic deletion of murine HK, the precursor of BK, provided strong protection against ECM pathogenesis even without treating the parasitemia. *PbA*-infected BK receptor–deleted mice (combined *Bdkrb1^–/–^Bdkrb2^–/–^*, *Bdkrb2^–/–^*, or *Bdkrb1^–/–^*) also had reduced ECM severity, with protection from neurologic deterioration and brain Evans Blue dye extravasation, corroborating the assessment that the biologically active peptide BK contributes to vasogenic edema. It is important to appreciate that this effect on ECM was not directed at the parasite, but the vertebrate host’s response manifested as cerebral edema. Experimental studies with cultured human brain vascular endothelial cells have shown that both the B2 and, to a lesser extent, B1 BK receptor, contribute to loss of barrier function, a critical step in vasogenic edema formation (15). In addition to stimulation of its own receptors, local BK formation may be proximal to barrier function changes via the VEGF-VEGFR2 and angiopoietin 1–TIE2 systems. BK stimulates VEGF production, which is elevated in CM, and the BK B2 receptor transactivates VEGFR2 (KDR/Flk-1) (46–48). BK also mediates its effect on barrier function through interacting pathways with angiopoietin 1–TIE2. Angiopoietin-2–null mice inhibit BK-stimulated vascular leakage (49). Moreover, overexpression of angiopoietin-1 blocks BK-induced vascular leakage and blockage of angiopoietin-1 promotes it (50). Additional studies are needed to examine the barrier function connections between the BK receptors and VEGF/VEGFR2 and the TIE2-angiopoietin systems.

**Figure 8:**
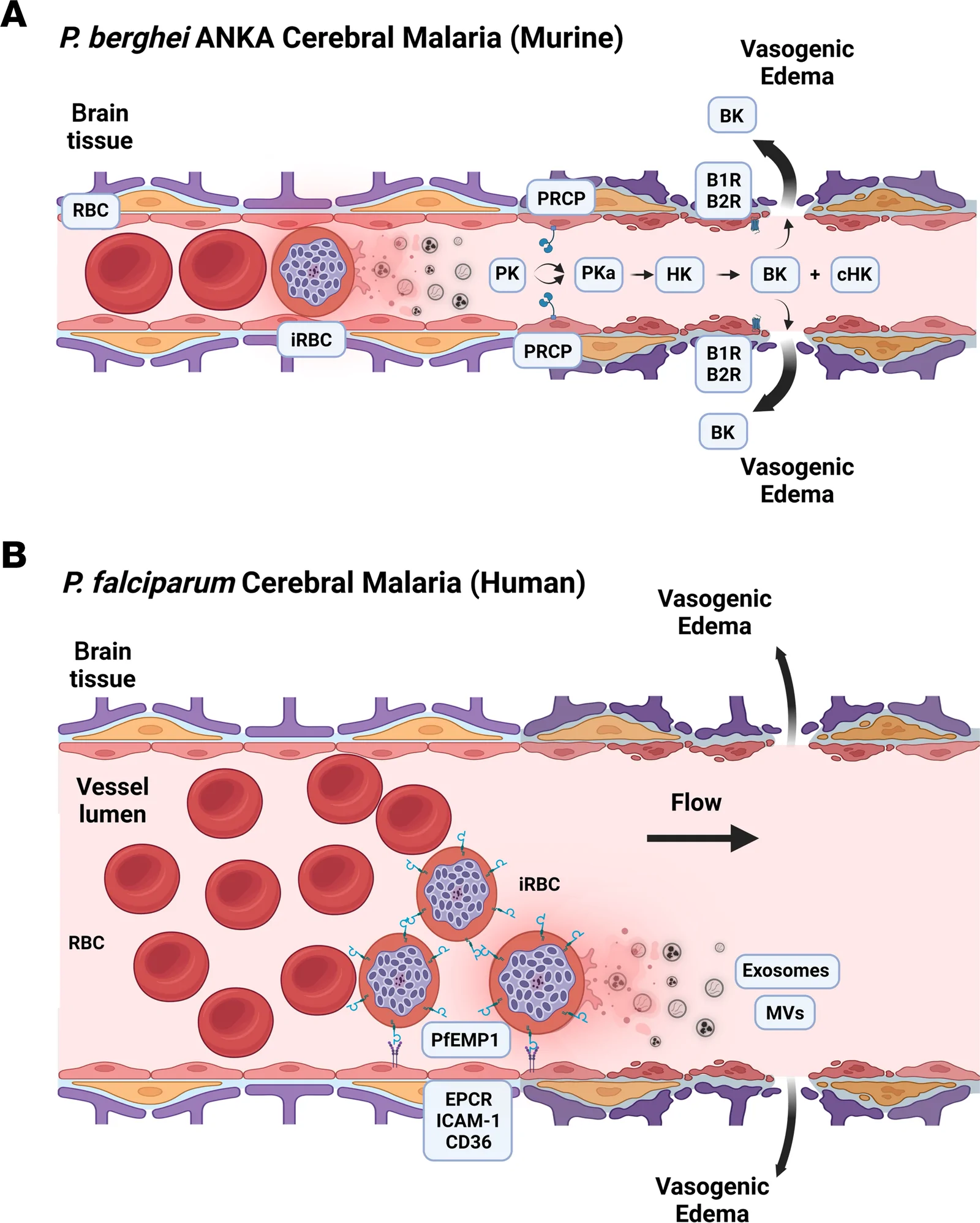
Vascular pathogenesis of murine and human cerebral malaria. (**A**) *P*. *berghei* ANKA (*PbA*) model of murine cerebral malaria. In *PbA*, single-cell brain microcapillaries became occluded with infected red blood cells (iRBC). The occlusion of the vessel and/or the effect of iRBC material plugging the vessel influences its endothelium to allow vessel wall prolylcarboxypeptidase (PRCP) to activate murine plasma prekallikrein (PK) to plasma kallikrein (PKa). PKa cleaves the HK bound to it to produce cHK liberating bradykinin (BK). Formed BK binds to the bradykinin B2 receptor (B2R) and the bradykinin B1 receptor (B1R). The B2R was the predominant receptor for BK binding. Activation of the B2R contributes to reduced endothelial cell barrier function and vasogenic edema. Gene deleted mice, *Kng1^–/–^*, *Klkb1^–/–^*, *Prcp^gt/gt^*, combined *Bdkrb1^–/–^Bdkrb2*^–/–^, *Bdkrb2^–/–^*, *Bdkrb1^–/–^*, or *Prcp^fl/fl^ Cre* mice showed significantly reduced brain edema and protection from neurologic deterioration. Alternatively, *F12^–/–^* mice do not have significant protection from neurologic deterioration. These data indicate that the PKa/kinin systems contributed to the vasogenic edema in *PbA*-induced murine (experimental) cerebral malaria. (**B**) Proposed mechanisms in human cerebral malaria pathogenesis. Unlike that in mice, human CM occurs in larger postcapillary venules where single iRBCs do not occlude the vessel. *P*. *falciparum*–infected RBCs express on their surface a protein called PfEMP1 (plasmodium falciparum membrane protein1) that binds the iRBCs to the vessel wall via endothelial cell membrane proteins that include endothelial cell protein C receptor (EPCR), ICAM-1, and CD36. BK liberation and vasogenic edema could be initiated by adherent iRBCs themselves or others that have ruptured. iRBCs, microvesicles, and/or exosomes also could serve as a source for falcipain-2 that can liberate BK from HK itself and, indirectly, generate BK through surface contact activation of factor XII by autoactivation and/or stimulate the endothelial cell membrane–associated enzyme prolylcarboxypeptidase. Both enzymes lead to activation of prekallikrein and the PKa/kinin system. **A** and **B** were produced with BioRender.

In mice we identified what we believe to be a novel mechanism for activation of the kallikrein/kinin system in ECM. Since the *Pf* protease falcipain-2 cleaves HK to produce cHK and BK (30), we were surprised to observe that *PbA* infection with trophozoites that did not express the falcipain-2 ortholog berghepain-2 were not protected from ECM. Furthermore, we did not anticipate that *F12^–/–^* mice would have the least protection from ECM compared with all other murine deletion models examined. We speculate that the minor effect of FXII deletion on Evans Blue dye extravasation observed was the result of an intact PK activation system that did not have a burst in PKa formation by reciprocal activation of formed factor XIIa. Alternatively, PK deficiency was highly protective against ECM and led us to consider another PK activator independent of FXII. PRCP is an endothelial cell S1 serine protease that is an activator of PK to PKa independent of FXII (31, 51). We observed that *Prcp^gt/gt^* mice, C57BL/6J wild-type mice treated with a PRCP inhibitor (34), and *Prcp* brain microvascular conditional KO mice had reduced neurologic deterioration and Evans Blue dye extravasation. Endothelial cell PRCP activated PK to PKa independent of FXIIa. The activated PKa then cHK to liberate BK, which bound both the B2R and the B1R and produced vasogenic edema (Figure 8A) (34). This pathway is initiated at the vessel wall and may be unique to murine ECM since microvascular vessel occlusion is the observed mechanism (52)

Our ECM studies suggest that the entire PKa/kinin system, not a single substrate (e.g., HK), enzyme (e.g., PKa, PRCP), or receptor (e.g., B2R, B1R), is a host factor that contributes to ECM pathogenesis. Elimination or interference with the function of any kallikrein/kinin system component ameliorates ECM. In theory, targeting any of these proteins by any technique could be contributory to improvement of the outcome of ECM. Since CM and ECM are disorders of a swelling brain within the fixed space of the skull, treatments targeting the vasogenic edema itself along with antiparasite therapy may produce improved outcomes in children. This notion was first demonstrated in mice by Higgins et al. in their investigation that showed that the addition of angiopoietin 1 infusion to mice treated with artesunate significantly increased survival in the ECM (14). Our investigation of the BK system had the same conclusion on the importance of adjunct treatment of CM to decrease brain edema along with antiparasite therapy. Artesunate-based antiparasite therapy is currently the standard-of-care treatment for pediatric CM. Clinical recovery is often observed within approximately 2 days, the time it takes to reduce the parasite biomass. In the mouse model, we showed that at 24 hours after adding PKa inhibition to artesunate therapy, there is (a) improvement in neurologic behavior, (b) reduction in brain Evans Blue uptake, and (c) increased survival compared with artesunate therapy alone. At 25 days, 13 days after stopping treatment, survival of the combined treated mice is 168% better than in mice treated with artesunate alone. These data indicate that, in addition to ongoing efforts to treat severe malaria with antiparasite drugs, adjunctive treatment that targets cerebral vasogenic edema by any of several pathways may substantially increase survival by more rapidly reducing brain swelling. This approach may also have potential to improve neurocognitive outcomes in surviving children.

It is well documented that human CM due to *Pf* infection and ECM due to *PbA* infection are different in several ways. *Pf*-infected RBCs are sequestered in medium- and large-sized brain vessels that express RBC surface proteins such as PfEMP-1 that bind to endothelial cell protein C receptor (EPCR), ICAM, and CD36 on the vessel wall (Figure 8B). In contrast. *PbA* iRBCs occlude mouse brain microvascular vessels where the diameter of the arteriole or capillary is about the same size of the diameter of the RBC (Figure 8A) (52). Normal flexible RBCs pass through vessels readily, but infected RBCs have reduced flexibility and occlude the vessel leading to distal thrombus (Figure 8A). Although these features distinguish human CM and mouse ECM from each other, the final common pathway for vasogenic edema in both may be due to BK formation and similar pathophysiology in the brain. Notably, blood-brain barrier dysfunction with MRI evidence for vasogenic edema, brain accumulation of cytotoxic CD8^+^ T cells, and extravasation of both uninfected and iRBCs occur in both (12, 13, 52–56). If BK is pathogenetic for the vasogenic edema in *Pf* CM, its formation could arise from different mechanisms in human CM and mouse ECM. In humans, BK formation in *Pf* could occur by falcipain-2 directly cleaving HK; FXII autoactivation on parasite, RBC, or endothelial cell extracellular vesicles; activating PK, which leads to BK formation; or vessel wall PRCP activating PK independent of FXIIa (Figure 8B). However, regardless of the precise mechanisms of BK formation, if BK is present, it will induce vasogenic edema in humans through mechanisms like those observed in mice. Therefore, the same therapeutic agents effective in ECM may also be applicable in humans.

### Limitations of the study.

The hypothesis that BK is a mediator of *Pf* CNS-M could not be confirmed in the Kenyan children because plasma samples were collected in sodium heparin without protease inhibitors to prevent in vitro formation and degradation of BK. Furthermore, plasma collected into sodium heparin obviated our ability to measure residual PK, FXII, or FXI in plasma and interfered with C1INH antigen studies. Additional studies are needed to determine if *PbA*-infected mice treated with artesunate plus RZLT7824 have long-term preserved neurologic and cognitive function relative to treatment with artesunate alone.

## Methods

### Sex as a biologic variable.

Our study examined male and female children, and similar findings are reported for both sexes. Likewise, our study examined male and female animals, and similar findings are reported for both sexes.

### Animal studies.

Wild-type C57BL/6J mice were purchased from The Jackson Laboratory. All KO mice were on a C57BL/6J background. All mice (wild-type and KO) had murine ECM when infected with *PbA*. *Kng1*^–/–^ KO animals were obtained in-house (29). *F12*^–/–^ mice have been previously described (57). *Klkb1*^–/–^ mice were developed at the Texas Genomic Institute and provided by Edward Feener, Beth Israel Hospital, Harvard University, Boston, Massachusetts, USA (58). *Prcp*^gt/gt^ mice were generated at the University of Michigan from KST302 ES cells obtained from Bay Genomics (32). *Bdkrb1^–/–^* mice were obtained in-house (59). *Bdkrb2^–/–^* mice (strain B6/129S7-*Bdkrb2*^tm1Jfh^) were purchased from The Jackson Laboratory. These animals were backcrossed 10 generations onto a C57BL/6J background (60). The double BK B1 and B2 receptor–deficient mice (*Bdkrb1^–/–^Bdkrb2^–/–^)* were provided by Rafal Pawlinski and Erica Sparkenbaugh, from the University of North Carolina, Chapel Hill, North Carolina, USA (61). Immunoblots of *Kng1^–/–^, Klkb1^–/–^,* and *F12^–/–^* plasma from each of these mice had no plasma protein on immunoblot, and the *Prcp^gt/gt^* mice had no PRCP antigen on immunoblot of kidney. Purchased C57BL/6J wild-type mice were used as simultaneous controls throughout these experiments.

### Preparation of brain endothelium PRCP conditional KO mice.

*Prcp* conditional KO mice (C57BL/6JSmoc-*Prcpem(flox)/Smoc*) (*Prcp^fl/fl^*) (catalog NM-CKO-230233) were created at Shanghai Model Organisms Center Inc., Shanghai, China (62). Exon 4 of the *Prcp* transcript was the floxed region (Figure 7A). The targeting construct using CRISPR/Cas9 gene editing was composed of FRT-splice acceptor-IRES-lacZ-polyA-loxP-neomycin cassette-polyA-FRT-loxP inserted upstream of exon 4. A second loxP site was inserted downstream of exon 4. Flp-mediated recombination removed the FRT-flanked lacZ and neo cassette, leaving exon 4 floxed. Genomic DNA was isolated from the tail, and the *Prcp^fl/fl^* mice were genotyped by PCR with 32 cycles of 94°C for 30 seconds, 58°C for 30 seconds, and 72°C for 5 minutes. The forward (TCCGCCTGGCTAAATGTTCA) and reverse (CGGCAGAGGTTCAGAACAGA) genotyping primers defined a 248 bp band in wild-type mice, a 343 bp band in homozygous floxed mice, and both bands in heterozygous mice with the floxed gene (Figure 6B).

To prepare the conditional KO, *Slco1c1-CreER^T2^* mice were obtained from Markus Schwaninger at the University of Lubeck, Lubeck, Germany. These mice take advantage of the selective expression of the thyroxine transporter, *Slco 1c1*, in brain endothelial cells (35). Briefly, a bacterial artificial chromosome was chosen to contain the mouse *Slco1c1* locus, and by homologous recombination the Cre recombinase (iCre) and a mutated human estrogen receptor (*ER^T2^*) were electroporated into bacteria. The cloned genomic fragment with the *iCreER^T2^* was inserted at the ATG site of the *Slco1c1* gene (35). Transgenic offspring were created by microinjection into B6D2F1 hybrid mouse pronuclei (35). Genomic DNA was isolated from the tail, and the *Prcp* mice were genotyped by PCR by 30 cycles of 94°C for 30 seconds, 60°C for 30 seconds, and 72°C for 45 seconds. The forward (CTAGGCCACAGAATTGAAAGATCT) and reverse (GTAGGTGGAAATTCTAGCATCATCC) genotyping primers defined a Cre^–^ 324 bp band in wild-type mice. A 521 bp band produced by the forward (GCTATTCATGTCTTGGAAGCC) and reverse (CAGGTTCTTCCTGACTTCATC) genotyping primers defined the presence of the *Cre^+^*. Demonstrating both bands defines hemizygous transgenic mice containing the floxed gene (Figure 6B) (*Prcp^fl/fl^ Cre*^+^). Brain endothelial cell deletion of mouse PRCP was performed by mating homozygous *Prcp^fl/fl^* mice with transgenic hemizygous *Slco1c1-CreER^T2^* mice. The *Cre* recombinase in the *Prcp^fl/fl^ Cre* mice was induced by 50 mg/kg tamoxifen for 5 days.

Additional validation that brain endothelial cell PRCP was removed was performed by immunofluorescence. Briefly, mouse brain and kidney sections were incubated with antibodies against CD31 and mouse PRCP (See Supplemental Table 1 for a complete description of the primary and secondary antibodies used in these studies). The goat anti-mouse polyclonal antibody against mouse PRCP (anti-TDN20) was reared with a peptide from the mouse PRCP amino acid sequence TNDFRKSGPYCSESIRKSWN at Q.C.B. Custom Antibody Service (33). Quantification of CD31 and PRCP expression in the brain sections and kidney was performed with assistance of FIJI software (63). Immunofluorescence micrograph channels were separated, and regions of interest (ROIs) were selected using the straight-line tool. In the “Analyze” toolbox, Plot Profile was selected. Values of distance and signal intensity were plotted in *xy* axis graphs using GraphPad Prism 10.6.1. The signal from the same ROI in the green and red channels was obtained and plotted as indicated. Regions where both signal intensities increased were considered as colocalization. Regions where the signal intensity was flat indicated no antigen present.

### Parasites.

The wild-type rodent malaria parasite, *PbA*, MRA-868 7, was obtained through BEI Resources. Transgenic *PbA* parasites (*PBANKA^–/–^*) (line 1619 strain, https://www.pberghei.eu/index.php; RMgm, 32) that lack the gene encoding Berghepain-2 (PBANKA_0932400) were obtained from the Leiden Malaria Research Group, Leiden University Medical Center (64). Both wild-type and transgenic parasite lines have been maintained by combination of passage in C57BL/6J mice and cryogenic storage of iRBCs (65).

Additional methods are provided in the Supplemental Methods.

### Statistics.

Data from the patient and ECM studies were compared in the text and graphed as the mean ± SD with individual values indicated unless otherwise stated. Each point represented an individual patient or animal. The total number of patients or animals in each group was described in the figures or in figure legends. All data of various animal groups were examined for Gaussian (normal) or nonparametric distribution (e.g., Shapiro-Wilk). For data that were normal in distribution, comparison between 2 groups was performed by a 2-tailed (paired or unpaired) *t* test, and comparison among 3 groups was performed by 1-way ANOVA. Nonparametric data were evaluated by Mann-Whitney or Kolmogorov-Smirnov tests. When multiple comparisons of data were made, both were examined by ANOVA. Parametric data were corrected using Tukey’s test, and nonparametric data were corrected with Dunn’s test. Statistical significance was defined as *P* < 0.05. Kaplan-Meier statistics were used to characterize survival curves. The Mantel-Cox and Gehan-Breslow-Wilcoxon tests were used to determine significant differences between the groups. Statistical data were analyzed using PRISM 11.0.

### Study approval.

Ethical review and approval for the collection of samples from Kenyan children was obtained from the Kenya Medical Research Institute Scientific and Ethical Review Unit (SERU protocol 3485, Nairobi, Kenya). University Hospitals Cleveland Medical Center/Case Western Reserve University IRB (no. 12-16-15) approved collection of human plasma from normal human donors for assay standardization at Case Western Reserve University (IRB CASE 19Z05, no. 01-06-02).

All studies were approved by Case Western Reserve University Institutional Care and Use Committee (protocol 2014-0089) and performed in accordance with the guidelines of the American Association for Accreditation of Laboratory Animal Care and the NIH. Experiments were performed using 6- to 8-week-old male and female mice.

### Data availability.

All supporting data values provided in figures, text, and supplemental materials are in the Supporting Data Values file. Each tab refers to all the data in each figure in the manuscript text or supplemental materials. All reagents and animals used in these studies are available upon request to the authors.

## Author contributions

AHS, JWK, and AASP conceived the project. ASP, DET, YJS, AAM, SS, RPSA, YSP, TG, BBB, and AED performed critical research. JWK, DM, SO, YSP, and AED acquired clinical patient samples. ASP, JWK, and AHS wrote the manuscript. ASP, RPSA, JS, CCN, AASP, OJTM, DG, MB, PJR, CJJ, KRM, BBB, and AED edited the manuscript. AHS is responsible for the final version.

## Conflict of interest

AHS is on the scientific advisory board of Rezolute Inc.

## Funding support

This work is the result of NIH funding, in whole or in part, and is subject to the NIH Public Access Policy. Through acceptance of this federal funding, the NIH has been given a right to make the work publicly available in PubMed Central.

- NIH grant AI130131 (to JWK and AHS).
- NIH grant HL157405 (to AHS).
- NIH grant HL143402 (to KRM and AHS).
- NIH grant HL144115 (to OJTM, DG, and AHS).
- Case Western Reserve University and University Hospitals Collaborative Science Award (to AHS and JWK).

## Supplementary Material

Supplemental data

Unedited blot and gel images

Supplemental video 1

Supplemental video 2

Supplemental video 3

Supplemental video 4

Supplemental video 5

Supplemental video 6

Supplemental video 7

Supporting data values

## Figures and Tables

**Table 1:**
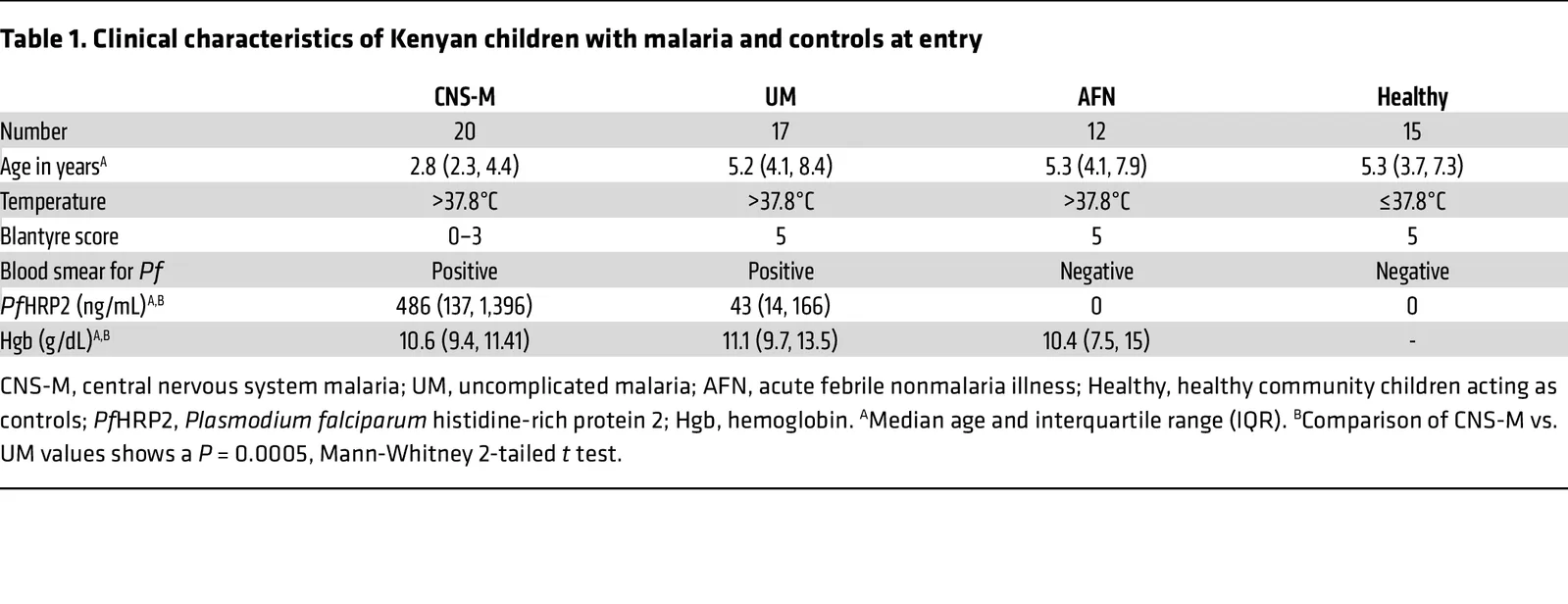
Clinical characteristics of Kenyan children with malaria and controls at entry

**Table 2:**
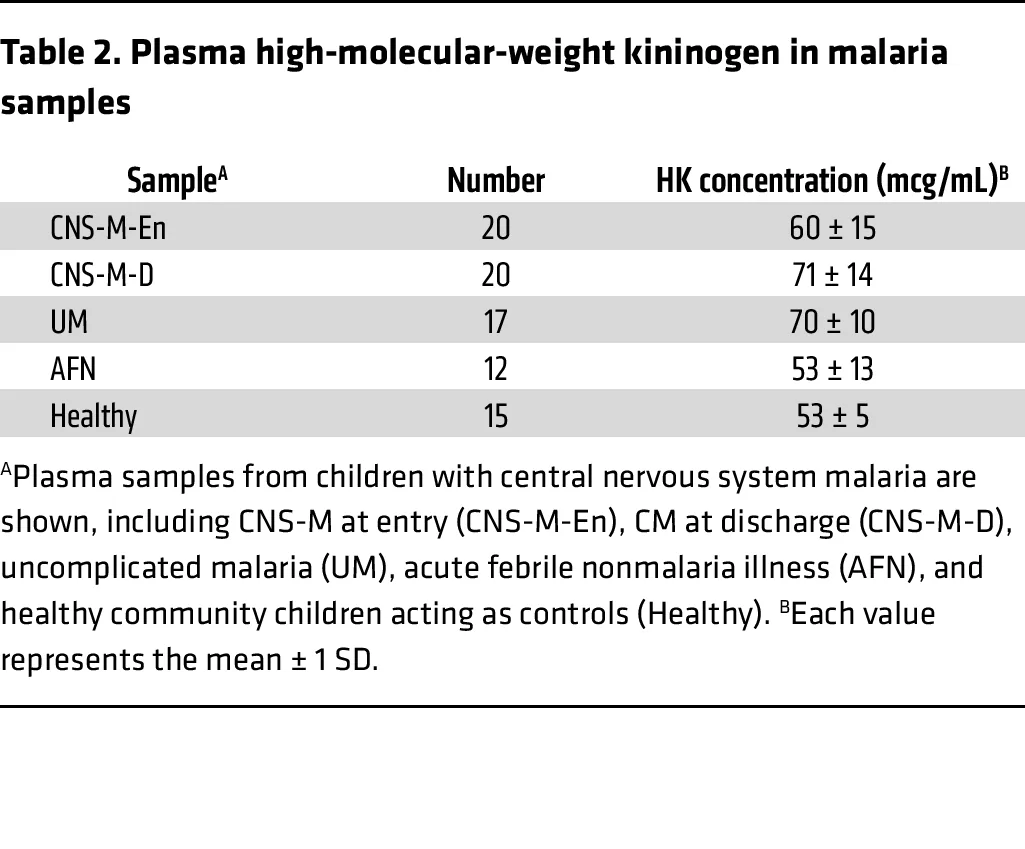
Plasma high-molecular-weight kininogen in malaria samples

## Version 1 07/14/2026

In-Press Preview

## Version 2 09/01/2026

Electronic publication
