# Supplementary material for: Bradykinin contributes to vasogenic edema in murine experimental cerebral malaria
Source: J Clin Invest. 2026 Jul 14;136(17):e202285. doi: 10.1172/JCI202285 (PMC13528921; doi:10.1172/JCI202285)
Supplement: Supplementary file 1 [file jci-136-202285-s159.pdf]

**Supplemental Information for:** 6-30-26

# Bradykinin Contributes to Vasogenic Edema in Experimental Murine Cerebral Malaria

Alessandro de Sa Pinheiro, Douglas E. Teixeira, Rodrigo Pacheco Silva-Aguiar, Young Jun Shim, Alona A. Merkulova, Sadiq Silbak, Yelenna Skomorovska-Prokvolit, David Midem, Sidney Ogolla, Bjoern B. Burckhardt, Tanja Gangnus, Julio Scharfstein, Celso Caruso-Neves, Owen McCarty, David Gailani, Michael Bader, Philip J. Rosenthal, Arlene E. Dent, Chris J. Janse, Keith R. McCrae, Ana Acacia de Sa Pinheiro, James W. Kazura, Alvin H. Schmaier*

***Address Correspondence to:**

Alvin H. Schmaier, M.D.

Email: Schmaier@case.edu

**Supplemental Methods**

*Materials.* The chromogenic substrates, H-D-Pro-Phe-Arg-pNA·2HCl (S-2302) (Cat #S820340) for plasma kallikrein. αFXIIa, and βFXIIa, H-D-Glu-Pro-Arg-pNA·2HCL (S2366) (Cat # S821090) for factor XIa, H-D-Phe-Pip-Arg-pNA·2HCl (S2238) (Cat # S820324) for thrombin (FIIa), and H-D-Val-Leu-Lys-pNA.2HCL (S2251) (Cat # S820332) for plasmin were purchased at DiaPharma. Z-D-Arg-Gly-Arg-pNA 2HCL (Biophen-CS11) (Cat # A229014) for factor Xa was purchased from Anaira Diagnostica. Purified single chain HK, PK, PKa, FXIIa, βFXIIa, FXIa, FXa, thrombin, and plasmin were purchased from Enzyme Research Laboratories (Cat # HT1300, Cat # HPK1302, Cat # HPKa1303, Cat # HFXIIa 1212a, Cat # HFXIIab, Cat # HFXIa 1111a, Cat # HFXa 1011, Cat # HT 1002a, and Cat # Hplasmin, respectively). The PRCP inhibitor (named "PrCP Inhibitor") was purchased from Sigma-Aldrich (Cat #5.04044). This compound is a brain-penetrant, dichlorobenzimodazolopyrrolidinamide compound with selective inhibition of PRCP of 1 to 2-8 nM of human or mouse PRCP in the absence or presence of albumin (S1). RZLT7824, a plasma kallikrein inhibitor from Rezolute, Inc., was generously provided by Dr. Jeffrey Breit, Rezolute, Inc., Redwood City, CA. Artesunate was purchased from Sigma-Aldrich (Cat # PH R2573).

*Plasma preparation from malaria study participants.* Peripheral blood samples from Kenyan children under 10 years with central nervous system malaria (CNS-M), uncomplicated malaria (UM), acute febrile non-malarial illness (AFN), or healthy community control children (Healthy) were collected into BD Heparin Blood Collection tubes at the Chulaimbo Sub-County Hospital in western Kenya (26). Please note that the term CNS-M, not cerebral malaria (CM), is used for these children in accordance with our recent study indicating that healthcare personnel did not have the capacity

perform ophthalmoscopic examination to evaluate retinal pathology (26). Blantyre scores of children diagnosed with CNS-M ranged from zero to 3. Ten of 20 children examined in the current report had Blantyre scores of ≤ 2. Plasma was prepared from these samples and were frozen at -80$^{o}$C. These samples remained frozen until assay at Case Western Reserve University. In control experiments, plasma from healthy malaria naïve North American adults was collected into sodium heparin (1 U/ml), EDTA (5 mM), or sodium citrate (3.2 gm%) anticoagulants and showed similar structural stability of plasma HK when incubated at 37$^{o}$C at 1 and 24 h and room temperature for 1 week. No sample showed spontaneous cleavage of intact 120 kDa HK into 56 and 46 kDa bands on reduced SDS-PAGE.

Blood from the CNS-M patients (See **Table 1**) was collected on admission to the hospital (CNS-M Entry, CNS-M-En) (26). CNS-M diagnosis was based on clinical presentation with seizures/coma (Blantyre score 0-3), fever, a *Pf* positive blood smear, and no other apparent cause of neurologic dysfunction. A paired blood sample was collected at the time of hospital discharge (CNS-M Discharge, CNS-M-D) 2 to 3 days later following completion of treatment with intravenous artesunate, clinical recovery with Blantyre score of 5, a lack of fever, and a *Pf* negative blood smear (26). Blood was also collected from children with uncomplicated malaria (UM) (*Pf* positive smear, no CNS symptoms or signs, Blantyre score = 5), children with acute fever non-malaria illness (AFN), and healthy children living in the same community. Healthy children did not have fever (axillary temperature ≤37.8$^{0}$C), had a *Pf* negative blood smear, and no history of clinical malaria in the previous 6 weeks. CNS-M patients were hospitalized for intravenous treatment with artesunate, but UM and AFN patients were treated as outpatients and did not have recovery blood samples obtained (27).

*Murine experimental cerebral malaria (ECM)*. C57BL/6J wild-type and gene deficient mice were inoculated IP with 0.1 mL suspension of $10^6$ *Plasmodium berghei* ANKA (*PbA*)-parasitized red blood cells (S2). The progression of murine CM (ECM) was evaluated by measuring *PbA* parasitemia and motor and neurological behavior assessment (SHIRPA score) on day 3, 4 and 5 after infection (28, S3, S4). The SHIRPA score is based on observations of the neurological behavior, including transfer arousal, locomotor activity, tail elevation, wire maneuvers, and contact righting reflex were observed (28). Each are scored from 0 to 40. A high number is normal. CM mice are below 20. Parasitemia was measured by BD-LSR II (BD Bioscience) flow cytometry using an optimized Hoectsh-Thiazole Orange staining strategy and confirmed by optical microscopy of Giemsa-stained thin blood smears by analyzing at least 10 random microscopic fields (S3). Final values were expressed as the percent infected RBCs. When the PRCP inhibitor (PrCP Inhibitor) was used with wild-type mice, the animals were injected intraperitoneally daily with 100 μl of the agent starting on day 3 of the infection with a calculated dose based on the estimated blood volume (7% body weight) to achieve a peak concentration of 100 μM inhibitor (34). When the plasma kallikrein inhibitor RZLT7824 was used with wild-type mice, the animals were injected IP with 100 μl of the solution that was calculated to be 60-80 mg/kg mouse every 12 h also starting on day 3. In investigations comparing artesunate alone versus artesunate and RZLT7824 together began at day 5 when the animals had objective neurologic defects. Artesunate was administered at a dose of 32 mg/kg in 100 μl IP once daily and RZLT7824 was given twice. Note: animal movies 1 and 2 (control C57BL/6J and genetic deletion mice) were made on day 5 when the control animals demonstrated objective

neurologic deterioration on the SHIRPA score. Animal movie 3 was made on day 6 after 24 h of treatment.

*Cerebral edema measurements.* The evaluation of cerebral edema was performed using the Evans Blue extravasation assay (S4). Briefly, on day 5 of *PbA* infection mice received an intravenous orbital injection of 1% Evans Blue dye solution when objective neurologic findings were observed in C57BL/6J wild-type mice. After 2 h, the mice were euthanized and their brain was harvested, weighed, and incubated in 1 ml of formamide (37°C, 24 h) to extract the dye. Absorbance of the supernatant was measured at 600 nm. The concentration of dye was determined using a standard curve. Data are expressed as micrograms of dye normalized per gram of tissue. Brain mass of wild type versus *PbA*-infected mice without or with treatment was determined by direct weighing of the excise brain from the cranial cavity.

*Murine complete blood count***.** On day 5 of *PbA* infection, whole blood was collected from infected and control mice anesthetized with isoflurane. Following a midline celiotomy, blood was drawn from the inferior vena cava using a 20-gauge needle attached to a 1 mL syringe containing 0.05 mL of 3.2% sodium citrate. Approximately 0.5 mL of blood was collected and centrifuged at 514 × *g* for 5 minutes at room temperature. The resulting plasma was either used immediately or stored in aliquots at –80°C. Complete blood counts were performed using a HEMAVET analyzer according to the manufacturer's instructions.

*General coagulant assays.* The activated partial thromboplastin time (aPTT) was performed by mixing 50 μl citrated plasma from infected and non-infected mice with 50

μl pre-warmed aPTT reagent (Helena Laboratories) in a glass tube and incubated for 5 min at 37°C. The reaction was initiated by adding 50 μl of 35.3 mM $CaCl_2$. The endpoint clotting time was determined visually by constantly tilting the glass tube in a 37°C water bath. The prothrombin time (PT) was performed similarly by the addition of 50 μl of pre-warmed PT reagent which contains $CaCl_2$ (Thromboplastin Reagent, Helena) to 50 μl citrated plasma from infected and non-infected mice in a glass tube.

*Coagulation factor assays.* HK, FXI and FXII plasma levels were measured in infected and non-infected mice using human HK-, FXI- and FXII-deficient plasmas as substrate in an aPTT-based coagulant assay, as described previously (S5). William's trait (total kininogen deficiency) human plasma donated to this laboratory was used as substrate for the HK assay. Factor XI- and XII-deficient human plasma was obtained from George King Inc, Overland Park, KS. A standard curve was created using a serial dilution from pooled normal mouse plasma to calculate the levels of these proteins in murine samples. The final values were expressed in IU/ml that is defined as the amount of factor activity or antigen in one ml pooled normal human or murine plasma.

*Prekallikrein chromogenic assay.* The PK levels in plasma from infected and non-infected mice were measured by chromogenic assay (S6, S7). Fifty μl of each plasma sample was treated with 50 μl of 0.167 M HCl for 15 min at room temperature to eliminate C1 inhibitor and other SERPIN plasma kallikrein inhibitors (S7). Then, 50 μl of 0.1 M Na phosphate, 0.15 M NaCl, 1 mM EDTA pH 7.6 buffer and 50 μl of 0.167 M NaOH were then added to neutralize the acid treatment. To determine the residual plasma PK activity, 50 μl of treated samples was incubated with 50 μl of plasma PK activator (260 pM of βFXIIa) for 5 min. The concentration of βFXIIa added to activate

the plasma PK was previously shown to be insufficient to hydrolyze the chromogenic substrate itself. After the activation period, 50 μl of the chromogenic substrate S2302 (2 mM) was added, the reaction was stopped after 10 min of hydrolysis using 100 μl of 50% acetic acid and the OD was obtained at 405 nm. A standard curve was created using a serial dilution from pooled normal mice plasma. The final values are expressed in IU/ml. One unit is the amount present in 1 ml of pooled normal mouse plasma.

The specificity of RZLT7824 to inhibit isolated plasma kallikrein (PKa) and activated plasma prekallikrein in plasma was determined. The $IC_{50}$ of 1 nM PKa or 1 nM FXIIa using 30 to 3000 nM RZLT7824 were determined using 0.4 mM S2302. The $IC_{50}$ of 3 nM FXIa, FXa, thrombin, or plasmin using 30 to 3000 nM RZLT7824 was determined using 0.3 mM S2366, 0.15 mM Biophen-CS11, 0.3 mM S2238, or 0.4 mM S2251, respectively, in a Hepes buffer, pH 7.4.

*C1 inhibitor functional assay.* Plasma C1INH functional levels were performed by the assay of Shapira *et al*. modified for a microplate reader (S8). After mouse blood collection, 50 μl from infected and non-infected mouse plasma samples were first treated with methylamine (40 mM final concentration) for 2 h at 23$^{o}$C to eliminate plasma $\alpha_2$macroglobulin (74). Ten μl of the treated samples was diluted with 50 μl in PBS buffer containing 2 mg/mL of bovine serum albumin (fraction V). The reaction was started by adding 10 μl of plasma kallikrein (50 nM final concentration) to the PBS-BSA solution. The microtiter plate was previously prepared with each cuvette containing 0.6 mM of S2302 diluted in PBS-BSA total volume of 90 μL, at precisely at 0.5, 1.5, 2.5 and 3.5 min, 10 μl of plasma-treated plasma kallikrein was collected and added to different cuvette wells. After 4.5 min, the microtiter plate was inserted into the spectrophotometer

and absorbance was obtained at 405 nm for 15 min at room temperature. The half-life (T 1/2) of the kallikrein inhibition was determined by plotting the change in absorbance from 7 to 14 min and entering the ∆OD/min. The pseudo-first-order rate k' constant was calculated by dividing the ∆OD/min into the natural log of 2, 0.693, manually or using Prism 10.0. To obtain a C1 inhibitor final activity level, the k' constant was divided by the 2nd order rate constant of C1INH inhibition of plasma kallikrein 1.02 x $10^6$ $M^{-1}$. Since the mouse plasma was not independently characterized for its concentration of C1INH, all final values were normalized by the international standard that 1 ml plasma contains 1 unit of plasma C1INH (S9).

*Competitive ELISA for plasma high molecular weight kininogen (CELISA).* Plasma from children with CNS-M at hospital entry before artesunate treatment (CNS-M-En), CNS-M at discharge following clinical recovery (CNS-M-D), UM, acute fever non-malarial (AFN), and healthy community control children (Healthy) were obtained from whole blood collected into heparin anticoagulant. Plasma HK antigen levels in these samples were assayed by a competitive enzyme-linked immunosorbent assay (CELISA) as described previously using a human polyclonal anti-HK that detects both intact and cleaved HK (**Supplemental Table S1**) (S10, S11). The standard curve for the HK CELISA was performed with total kininogen deficient plasma reconstituted with known concentrations of purified HK. The HK assay has an intra-assay coefficient of variation (CV) of 7.7% and 8.6% with pure HK in total kininogen deficient plasma and normal human plasma, respectively, and an inter-assay CV of 8.8% of 10 runs over a 1-month period. All primary and secondary antibodies used are listed in **Supplemental Table S1**.

*Immunoblotting.* Normal human plasma, plasma from malaria patients, and infected and non-infected mice plasma were prepared for immunoblot for HK, PK, FXII, and C1 inhibitor by adding 5 μl and 10 μl of a 1:10 dilution of human or murine plasmas, respectively, in Laemmli sample buffer containing 5% β-mercaptoethanol and heated at 95$^{o}$C for 5 min. The relative concentration of protein in plasma was determined by performing immunoblots on 10-100% normal mouse plasma (NMP) samples prepared identically to unknown murine samples. All bands of the protein of interest (NMP or *PbA*-infected plasmas) were compared to transferrin and band intensity was determined by ImageJ. Samples were run on either a reducing 7.5% polyacrylamide Tris-HCl Criterion gel (Bio-Rad) or a 10% polyacrylamide Tris-Glycine gel, as indicated. In certain experiments, the gels were stained with Coomassie blue. In other experiments gel electrophoresis was followed by transfer to Immobilon-P membranes (Millipore, Bedford, MA, USA). The primary and secondary antibodies and dilutions used for immunoblotting are described in **Supplement Table S1, Supplement Table S5**. Protein bands were visualized with ECL substrate or Licor. Western blots were quantified by densitometric analysis (ImageJ). When immunoblots were used for quantitative analysis transferrin staining were used as loading controls.

Several antibodies to HK were used in these investigations (**Supplement Table S1, Supplement Table S5**). A goat polyclonal antibody to HK was used initially for the HK CELISA (S10). It was reared to purified HK in goats and was adsorbed with total kininogen deficient plasma (Williams plasma) and purified low molecular weight kininogen (S10). This antibody will detect total HK, intact and cHK. Cleaved HK was determined by the presence on immunoblot of the 56 and/or 46 kDa band(s) of the light chain of HK on samples reduced with 5% β-mercaptoethanol and heated for 5 min prior

to addition to the SDS-PAGE for later transfer for immunoblot. A rabbit polyclonal antibody reared to the human HK D5 peptide LDDDLEHQGGHVLDHGHKHK-HGHGHGKHKNKGKKNGK also was used to immunoblot human plasma HK. Two different rabbit polyclonal antibodies with similar sequences were prepared to detect murine HK D6 by immunoblot: anti-Cys-AHDDLIPDIHVQPDSLSFKLISDFPEAT or -AHDDLIPDIHVQPDSLSFKLISDFPEATSPK. Cys-AHDDLIPDIHVQ-PDSLSFKLISDFPEAT were prepared at BIOMATIK (Kitchener, ON, Canada). The other anti-mouse D6 antibody was generously provided by Dr. Keith McCrae, Cleveland Clinic Foundation. In certain experiments, the relative antigen concentrations of HK, PK, FXII, and C1 inhibitor in plasma from murine *P. berghei* ANKA infected blood were estimated by scanning the intensity of the antigen band(s) on immunoblots. Each sample's antigen level was compared to at least 8 normal mouse samples run on the same immunoblots after the values were normalized for loading with transferrin. Note: when using an anti-mouse D6 antibody, two bands for HK are present. Quantitative studies include both bands in the amount present in normal and *PbA*-infected murine samples. Results are expressed at IU/ml antigen concentration, 1 unit being the amount present in 1 ml of pooled normal mouse plasma.

*Bradykinin assay.* Blood samples from uninfected and *PbA*-infected mice for the bradykinin assay were collected into a cocktail of 7 protease inhibitors diluted 1:9 with blood at the final concentrations indicated: hexadimethrine bromide (3 mg/ml), nafamostat mesylate (6.9 μg/ml), EDTA (6.1 mM), sodium citrate (12.4 mM), formic acid (1%), omapatrilate (1 μM) and chloroquine (1 mM) (23). Plasma was prepared by centrifugation at 1200 xg for 5 min at room temperature in an Eppendorf centrifuge. The collected plasma was frozen at -80$^{o}$C until assay. Standards for the assay was the

addition of known quantities of bradykinin to murine HK deficient plasma from *Kng1*$^{-/-}$ mice whose plasma contains the same cocktail of inhibitors. The assay was performed at the Lerner Research Institute, Mass Spectrometry Laboratory for Protein Sequencing at the Cleveland Clinic, Cleveland, OH. At the time of assay, 100 μl samples were processed using a Waters Oasis μElute 96-well plate according to the manufacturer's recommendations. The eluted samples were dried in a Speedvac and reconstituted in 40 μl 0.1% formic acid. The LC-MS/MS (ThermoFisher Altis plus triple quadrupole mass spectrometer running in positive ion mode) was performed by injecting 20 μl of each processed sample onto a Gemini® 3 µm C18 110Å LC 150 x 2 mm column using a Vanquish UHPLC running at 0.30 mL/min with 3.2% DMSO in water and 0.1% formic acid as solvent A and 3.2% DMSO in methanol and 0.1% formic acid as solvent B. The data were processed using Thermo XCalibur software package. A processing method was developed using the transitions and calibration curve made by the standard samples, and the results were generated in Thermo XCalibur Quant Browser.

*Statistical Analysis.* Data from the patient and ECM studies were compared in the text and graphed as the mean ± SD with individual values indicated unless otherwise stated. Each point represented an individual patient or animal. The total number of patients or animals in each group was described on the figures or in figure legends. All data of various animal groups were examined for Gaussian (normal) or nonparametric distribution (e.g., Shapiro-Wilk). For data that were normal in distribution, comparison between 2 groups was performed by a two-tailed (paired or unpaired) t-test and 3 groups by ANOVA. Nonparametric data was evaluated by Mann-Whitney or Kolmogorov-Smirnov tests. When multiple comparisons of data were made, both were examined by ANOVA. Parametric data were corrected using Tukey's test and

nonparametric data were corrected with Dunn's test. Statistical significance was defined as a $P < 0.05$. Kaplan-Meier statistics were used to characterize survival curves. The Mantel-Cox and Gehan-Breslow-Wilcoxon tests were used to determine significant differences between the groups. Statistical data were analyzed using PRISM 11.0

**Supplemental References**

S1. Graham TH, et al. Discovery of non-benzimidazole and brain-penetrant prolylcarboxypeptidase inhibitors. *Bioorganic & Medicinal Chemistry Letters*. 2012;22(1):658-665.

S2. Lacerda-Queiroz N, et al. Inflammatory changes in the central nervous system are associated with behavioral impairment in Plasmodium berghei (strain ANKA)-infected mice. *Exp Parasitol*. 2010;125(3):271-278.

S3. Grimberg BT, et al. A. Monitoring plasmodium falciparum growth and development by UV flow cytometry using an optimized Hoechst-thiazole orange staining strategy. *Cytometry A*. 2008;73(6):546-554.

S4. Thumwood CM, et al. Breakdown of the blood-brain barrier in murine cerebral malaria. *Parasitology.* 1988;96(Pt 3):579-589.

S5. Colman RW, et al. Williams trait. Human kininogen deficiency with diminished levels of plasminogen proactivator and prekallikrein associated with abnormalities of the Hageman factor-dependent pathways. *J Clin Invest*. 1975;56(6):1650-1662.

S6. Stavrou EX, et al. Reduced thrombosis in Klkb1$^{-/-}$ mice is mediated by increased Mas receptor, prostacyclin, Sirt1, and KLF4 and decreased tissue factor. *Blood*. 2015;125(4):710-719.

S7. De La Cadena RA, et al. Evaluation of a microassay for human plasma prekallikrein. *J Lab Clin Med*. 1987;109(5):601-607.

S8. M. Schapira, et al. New and rapid functional assay for C1 inhibitor in human plasma. *Blood*. 1982;59(4):719-724.

S9. Schmaier AH, et al. Plasma prekallikrein assay: reversible inhibition of C-1 inhibitor by chloroform and its use in measuring prekallikrein in different mammalian species. *J Lab Clin Med*. 1984:104(6):882-892.

S10. Schmaier AH, et al. High-molecular weight kininogen. A secreted platelet protein. *J Clin Invest*. 1983;71(5):1477-1489.

S11. Schmaier AH, et al. The expression of high molecular weight kininogen on human umbilical vein endothelial cells. *J Biol Chem*. 1988;263(31):16327-16333.

**Supplemental Figures & Legends**

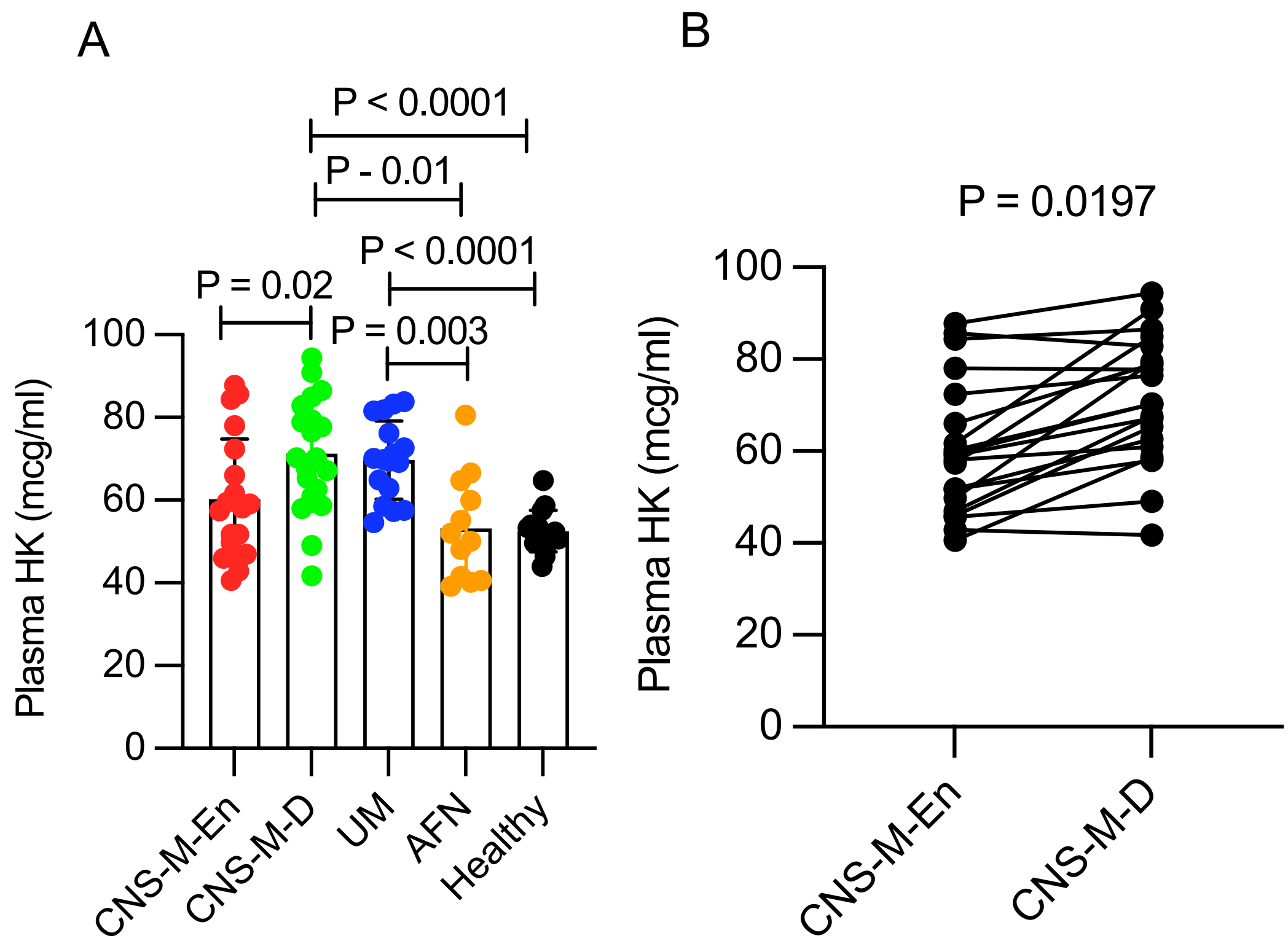


**Supplemental Figure S1**. . **A**. Plasma HK levels in children with central nervous system malaria (CNS-M) at hospital entry (CNS-M-En, n =20), and hospital discharge (CNS-M-D, n = 20), uncomplicated malaria (UM, n = 17), acute febrile non-malaria illness (AFN, n = 12), and healthy community control children (Healthy, n =15). HK levels were measured by CELISA and are presented as mean ± SD. The statistical analysis between groups was performed by unpaired two-tailed t test and one way ANOVA correcting for multiple comparisons using Tukey's test. P < 0.05 was considered significant. Statistical comparisons shown are for all significant difference between groups based upon the ANOVA and Tukey's test. The absence of comparison between groups indicates non-significant differences. **B**. Paired samples of plasma HK concentrations of CNS-M patients at hospital entry (CNS-M-En) and discharge (CNS-M-

D). The data were analyzed by group paired two-tailed t-test (n=20 paired samples to test).

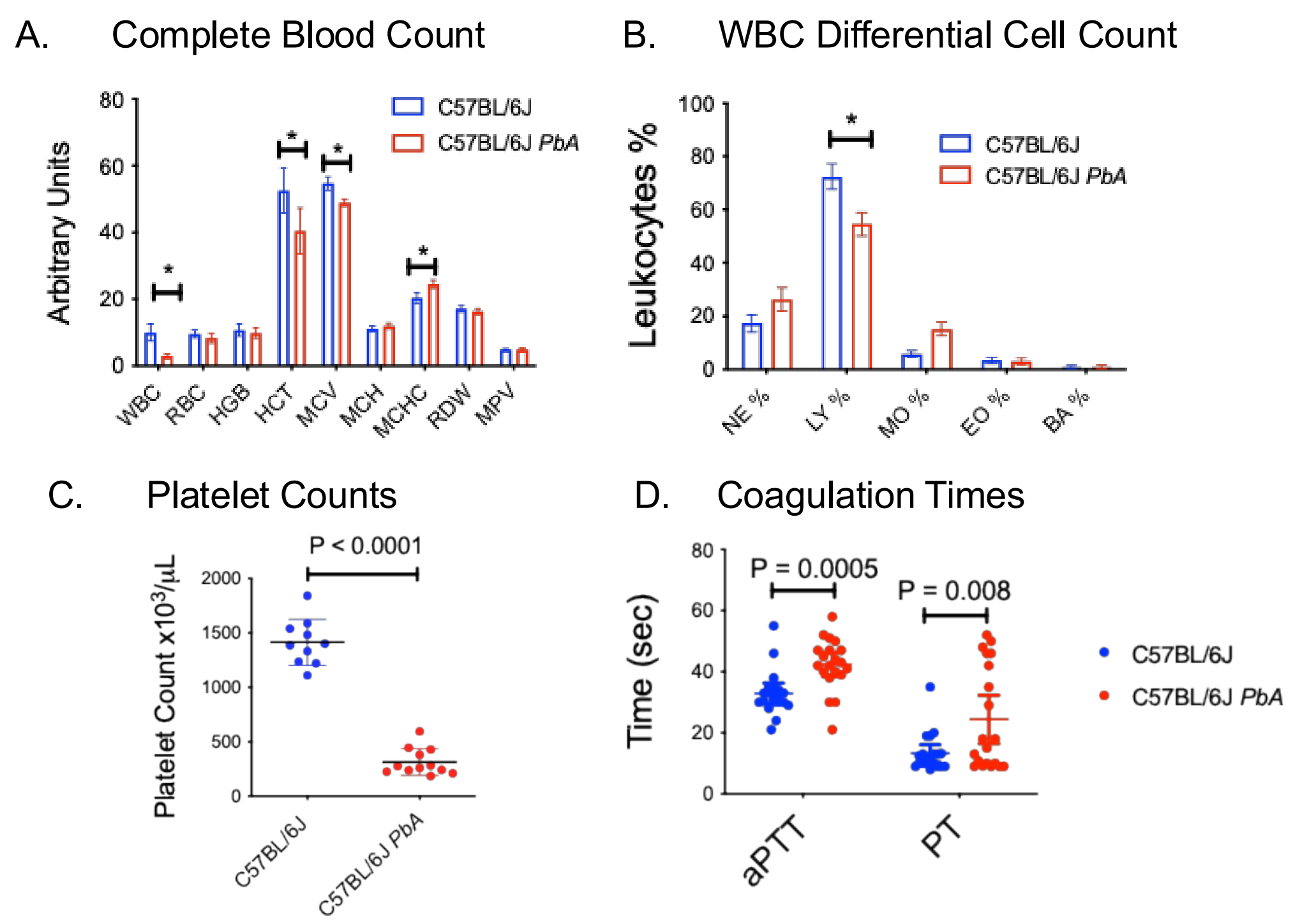


**Supplemental Figure S2**. Clinical laboratory parameters of *Plasmodium berghei* ANKA infected and uninfected C57BL/6J mice. **A**. Complete blood count of uninfected (C57BL/6J) (blue bars) and infected (C57BL/6J *PbA*) (red bars) mice. The data were the mean ± SD of 12 individual samples. An asterix (*) indicates P < 0.05. **B**. Leukocyte differential cell counts of uninfected C57BL/6J (C57BL/6J) and *PbA*-infected (C57BL/6J *PbA*) mice. The data were the mean ± SD of 12 individual samples. NE=neutrophils, LY=lymphocytes, MO=monocytes, EO=eosinophils, BA=basophils. **C**. Platelet counts of uninfected C57BL/6J (C57BL/6J) (blue dots) and *PbA*-infected C57BL/6J (C57BL/6J *PbA*) (red dots) mice. The data were the mean ± SD of 10-12 individual samples. **D**. The activated partial thromboplastin time (aPTT) and prothrombin time (PT) of uninfected C57BL/6J (C57BL/6J) and *PbA*-infected C57BL/6J (C57BL/6J *PbA*) mice. Data are presented as the mean ± SD CI of 20 individual samples. Comparison between uninfected and infected mouse samples were made by two-tailed t test, P < 0.05 was considered significant.

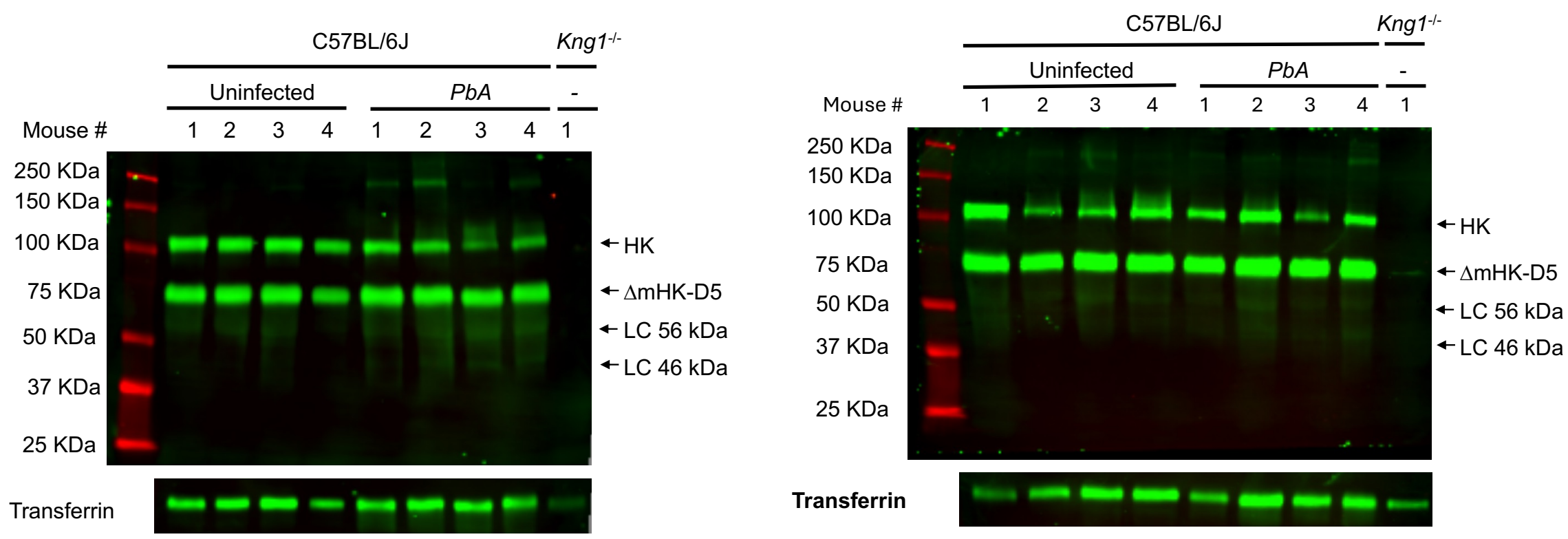


**Supplement Figure S3**. Mouse HK immunoblots for Figure 2C using a rabbit anti-peptide antibody to domain 6 of mouse HK (See Suppl. Table S5) on a 7.5% SDS-PAGE. This figure shows the 8 uninfected wild-type C57BL/6J mice and 8 *PbA*-infected wild-type mice. Each sample was normalized first for loading with transferrin. When normalizing the immunoblot samples, both bands of HK (HK and ΔmHKD5) were included. First, the wild-type uninfected samples were normalized. Then, the 8 infected samples were normalized against the mean of the uninfected wild-type mice. The data from these samples are seen in **Fig 2C**.

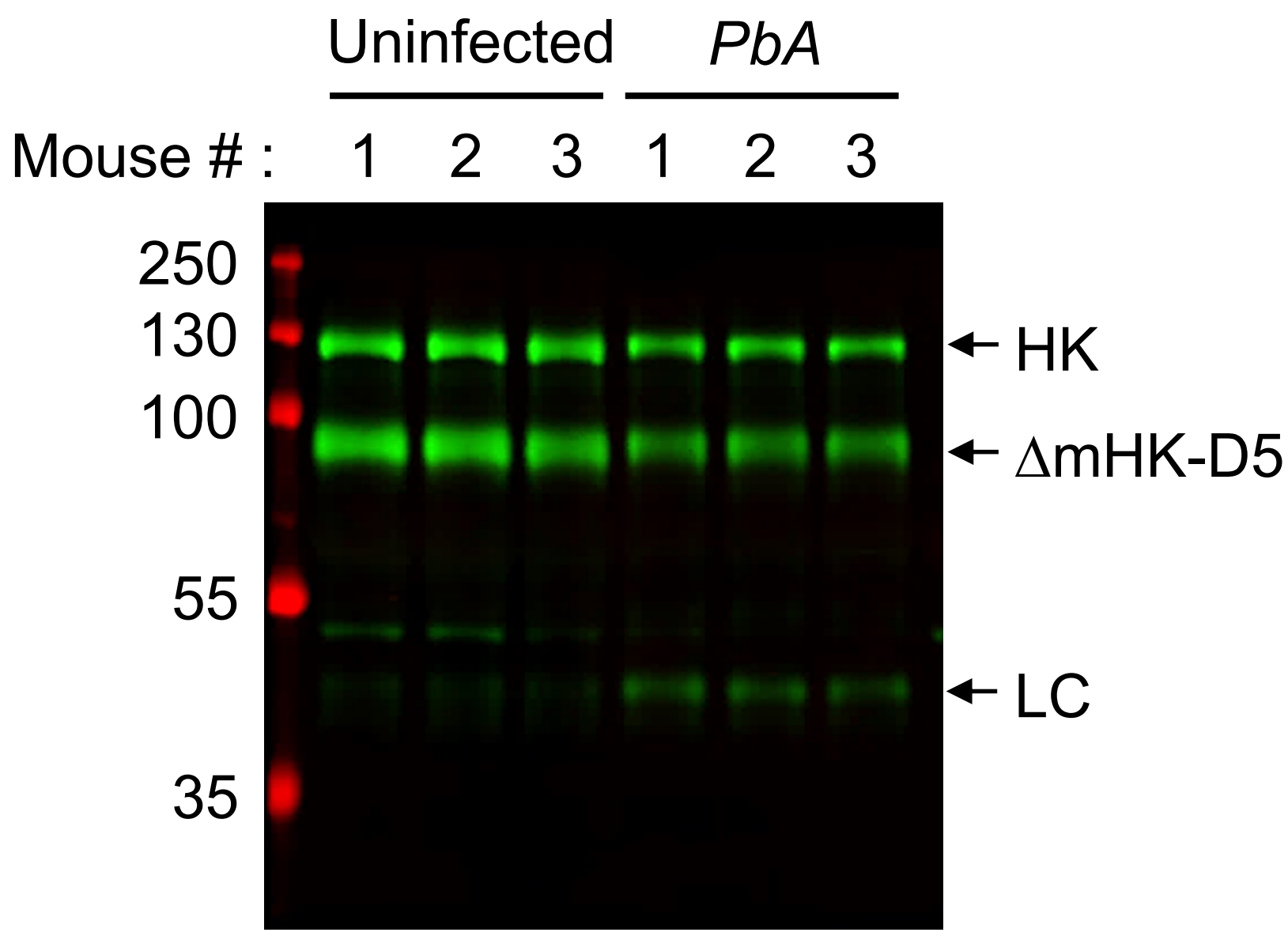


**Supplemental Figure S4**. Immunoblot analysis of plasma HK in uninfected (n=3) or *Plasmodium berghei* ANKA infected (n=3) samples using a second anti-murine HK D6 antibody (See Suppl. Table S5) on a 10% Tris-Glycine gradient SDS-PAGE. The antibody detects intact murine HK at ~130 kDa and the unique form of murine HK lacking D5 (ΔmHK-D5) at ~100 kDa in both groups. In infected mice, reduced intact HK and ΔmHK-D5 are accompanied by the appearance of the ~46 kDa HK light chain, consistent with HK cleavage.

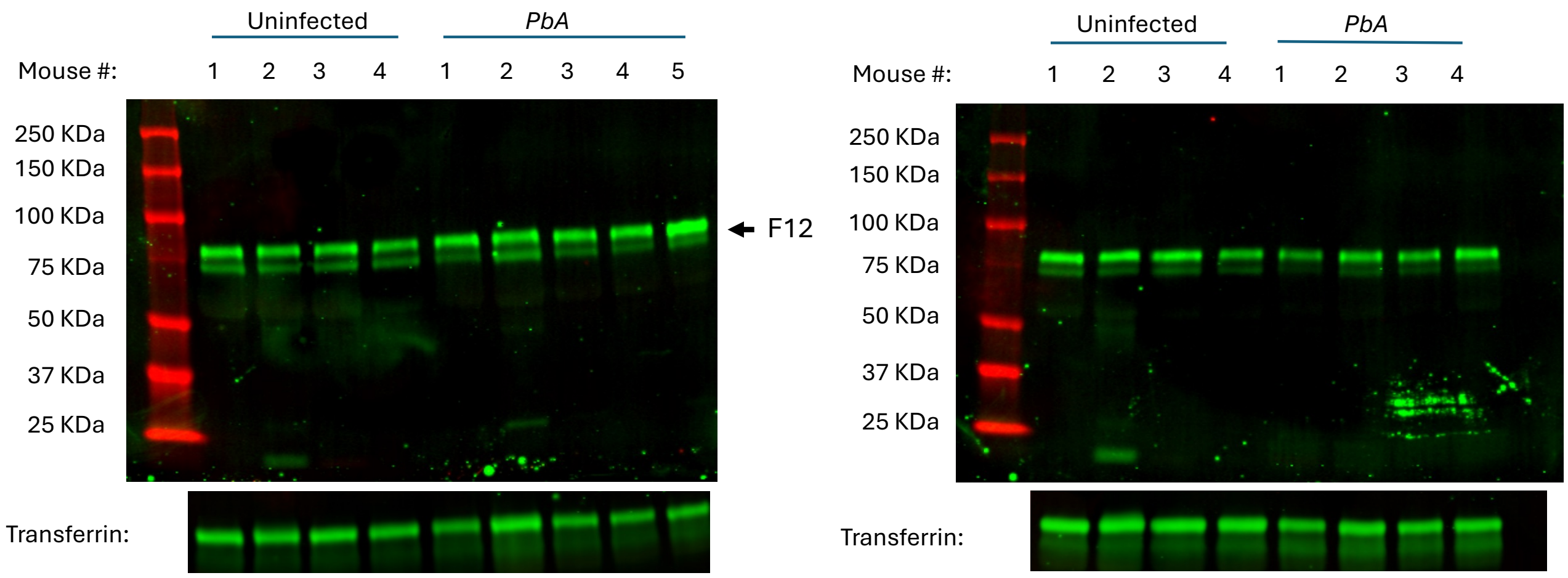


**Supplement Figure S5**. Immunoblots analysis of plasma Factor XII (FXII) from uninfected C57BL/6J mice (n=8) and *Plasmodium ber*ghei ANKA infected (*PbA*) (n = 9) mice. Samples were normalized to transferrin and densiometric values from infected mice were normalized to the mean of uninfected controls. Quantified data are presented in Figure 2G

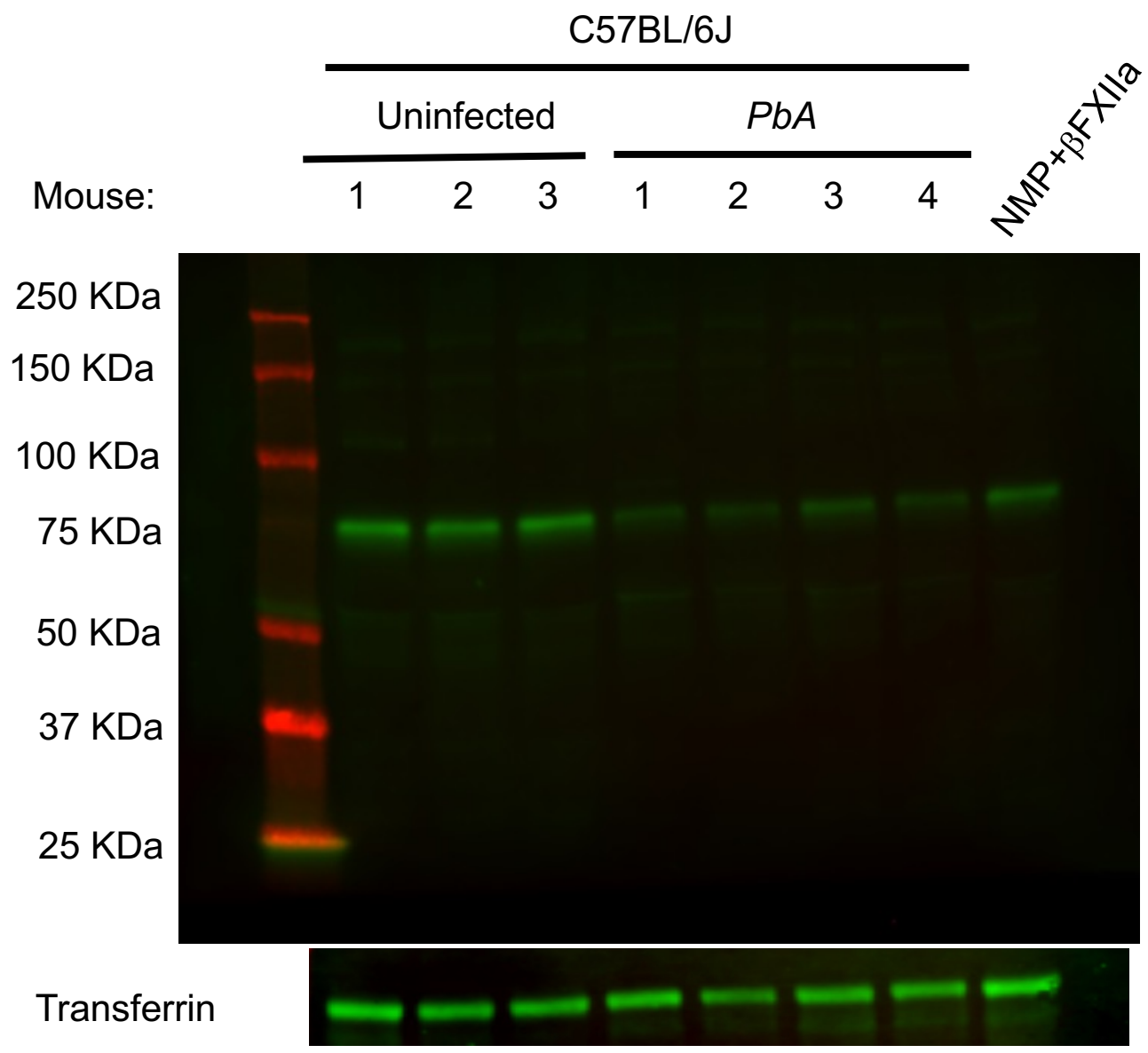


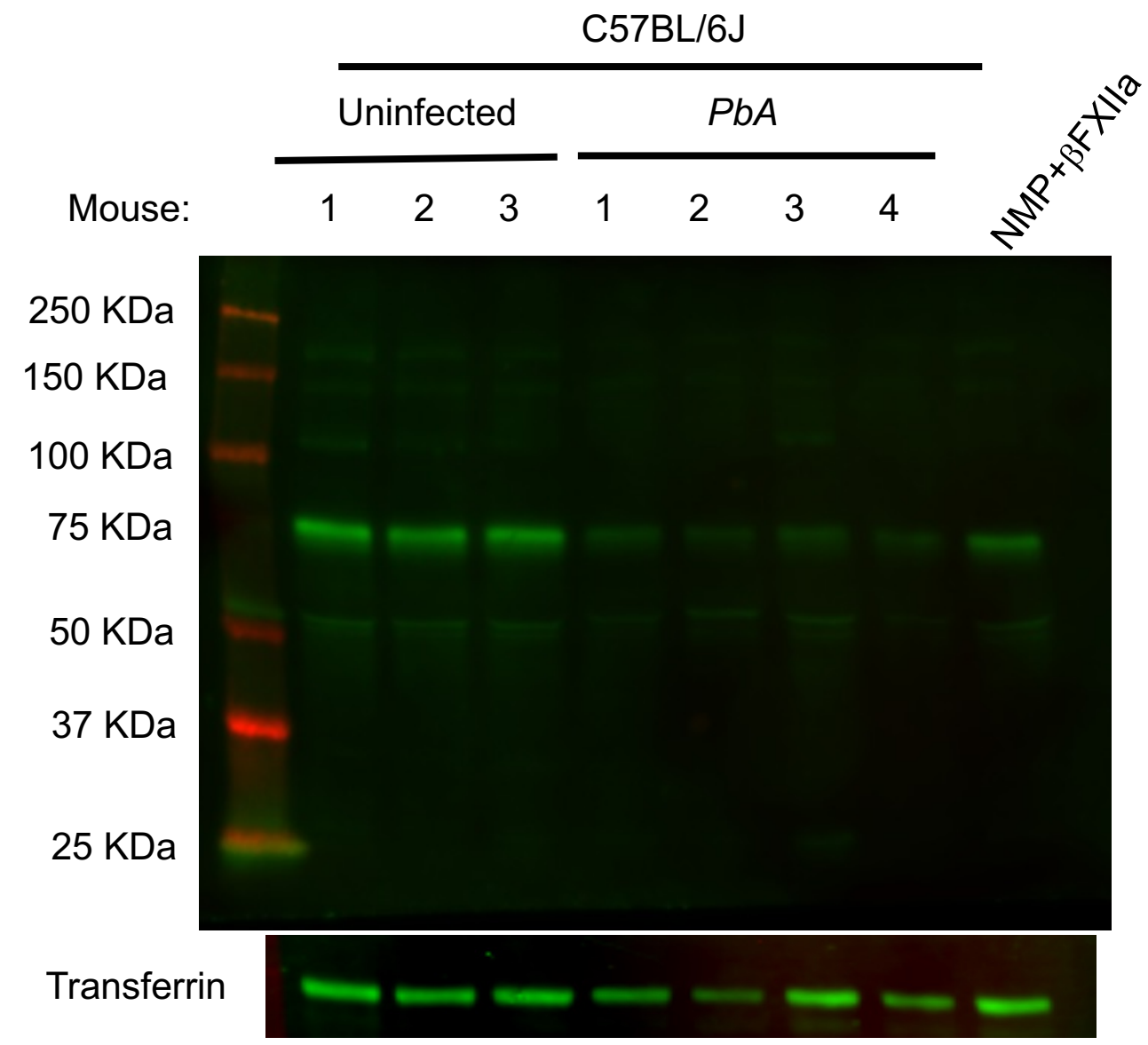


**Supplement Figure S6**. Immunoblot analysis of plasma prekallikrein (PK) from uninfected C57BL/6J (n = 6) and *Plasmodium berghei* ANKA infected (*PbA*) n = 8) mice. NMP+βFXIIa is normal mouse plasma treated with βFXIIa. Samples were normalized to transferrin and densitometric values from infected mice were normalized to the mean of uninfected controls. Quantified data are presented in Figure 2I.

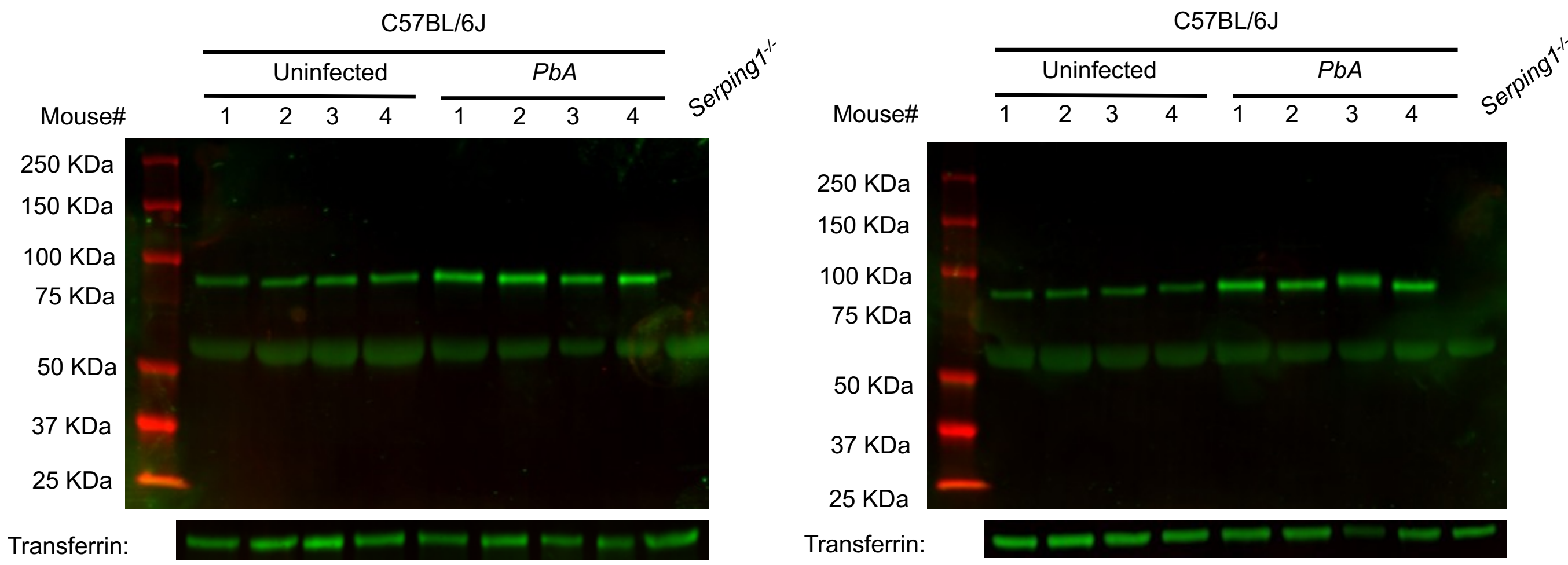


**Supplement Figure S7**. Immunoblot analysis of plasma C1 inhibitor from uninfected C57BL/6J (n = 8) and *Plasmodium berghei* ANKA (*PbA*) (n=8) mice. Samples were normalized to transferrin and densitometric values from infected mice were normalized to the mean of uninfected controls. Quantified data are presented in Figure 2 L.

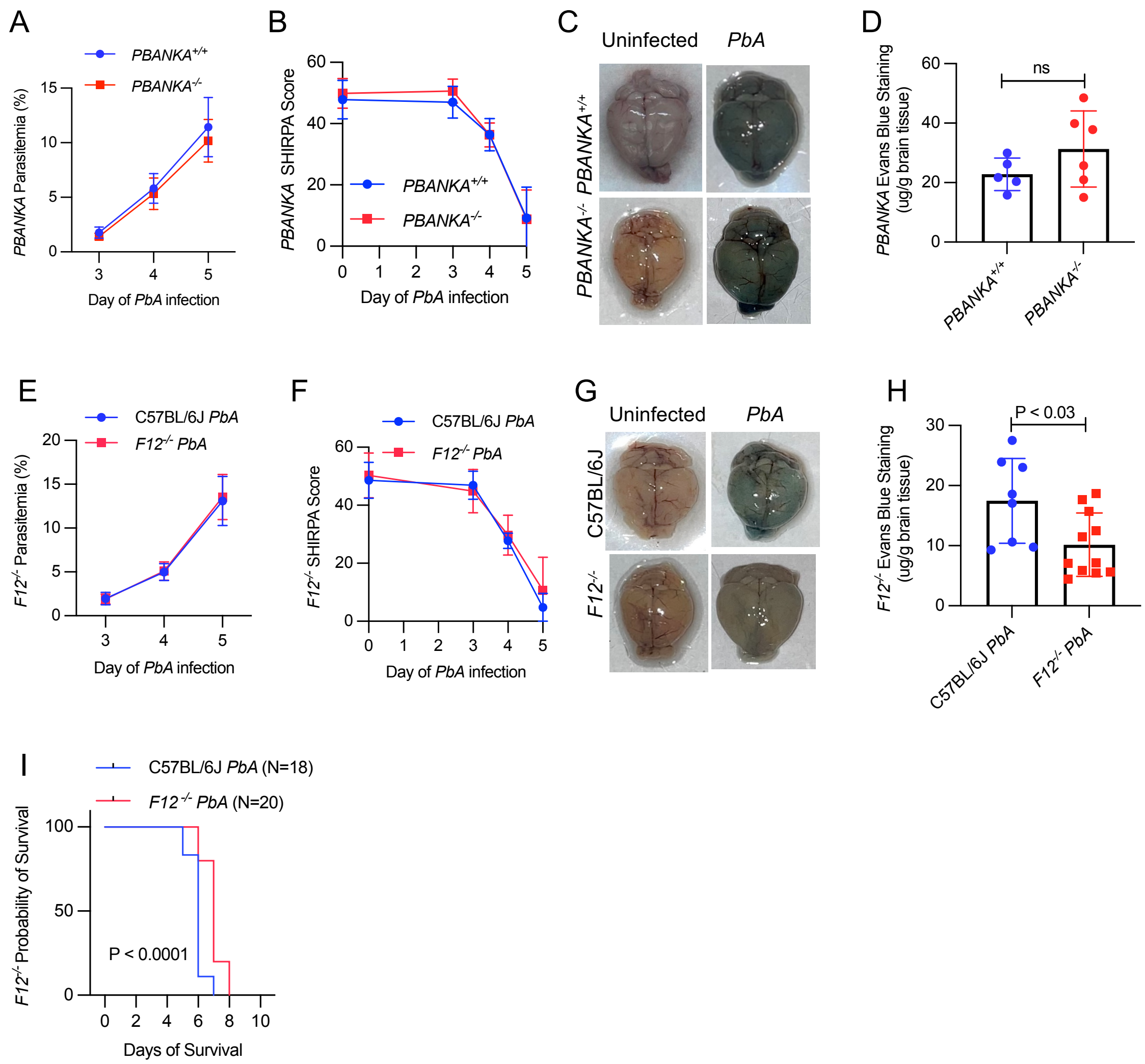


**Supplemental Figure S8**. Characterization of *PBANKA*$^{-/-}$ and *F12*$^{-/-}$ mice. C57BL/6J mice infected with *P. berghei* ANKA (*PBANKA*$^{+/+}$ *PbA*) or berghepain-2-deleted PbA (*PBANKA*$^{-/-}$ *PbA*). *PBANKA* are trophozoites that contain ($^{+/+}$) (n=5) or do not contain ($^{-/-}$) (n=6) the *P. berghei* cysteine protease berghepain-2. **A**. Parasitemia: **B**. SHIRPA Score. On day 5, *PBANKA*$^{-/-}$ mice had no protection from neurologic and motor deterioration of the *P. berghei* infection. **C**. Representative brain images following Evans Blue dye injection. **D**. Evans Blue dye staining in mice. These data were the mean $\pm$ SD of 1 experiment. Next, *F12*$^{-/-}$ mice infected with *P. berghei* ANKA were examined. **E**. Parasitemia, **F**. SHIRPA Score. On day 5, *F12*$^{-/-}$ mice had no protection from neurologic

and motor deterioration of the *P. berghei* infection. **G**. Representative brain images of Evans Blue dye uptake. **H**. Evans Blue dye staining in the brain was slightly less in the *F12*$^{-/-}$ than the control C57BL/6J mice. There were 8 uninfected C57BL/6J wild-type and 12 *F12*$^{-/-}$ mice in Figs. E, F, and G. **I**. Kaplan-Meier plot of survival of *PbA*-infected *F12*$^{-/-}$ mice (n=20) and infected wild-type C57BL/6J mice (n=18). Comparisons between groups on day 5 of the SHIRPA scoring and Evans blue uptake were performed by unpaired two-tailed t test, $P < 0.05$ was considered significant. Differences in the Kaplan-Meier plots survival curves were determined by Mantel-Cox and Gehan-Breslow-Wilcoxon tests performed independently.

**Supplemental Table S1:** Primary and Secondary Antibodies Used in the CELISA, Immunoblot, and Immunofluorescence Studies.

| Figure | Primary Antibody | 1°ab Species | Source, Cat# | Dilution or Concentration | 2°ab Species | Source, Cat# | Conjugate | Dilution or Concentration |
|---|---|---|---|---|---|---|---|---|
| Fig. 1A | Anti-Human Polyclonal HK | Goat | Custom made (Ref#S10) | 1:4000 | Rabbit anti-Goat | Sigma A-7650 | Alkaline Phosphatase | 1:3000 |
| Fig. 1C | Anti-Human HK D5 | Rabbit | Custom made (Anti-LDDD...) | 1 μg/ml | Goat ant-Rabbit | Licor, 926-32211 | IRDye 800CW | 1:5000 |
| Fig. 2C, | Anti-Mouse HK D6 | Rabbit | Custom made (Anti-Cys-AHDD...) | 2 μg/ml | Donkey anti-Rabbit | Jackson Immuno Res 711-035-152 | HRP | 1:5000 |
| Fig. 2D | Anti-Mouse HK D6 | Rabbit | Custom made (Anti-Cys-AHDD...) | 2 μg/ml | Goat anti-Rabbit | Licor 926-32211 | IRDye 800CW | 1:10000 |
| Fig 2G & Fig. S5 | Anti-Mouse FXII | Rabbit | American Research Products #PAA677Mu01 | 1 μg/ml | Goat anti-Rabbit | Licor 926-32211 | IRDye 800CW | 1:10000 |
| Fig. 2I & Fig. S6 | Anti-Mouse PK | Goat | R&D System AF2498 | 1 μg/mL | Donkey anti-Goat | Invitrogen A15999 | HRP | 1:5000 |
| Fig 2L & Fig S7 | Anti-Mouse C1INH | Rabbit | Proteintech #12259-1-AP | 0.3 μg/ml | Goat anti-Rabbit | Licor 926-32211 | IRDye 800CW | 1:10000 |
| Fig 6C & 6F | Anti-Mouse CD31 | Rat | BD Biosciences #550274 | 1:25 | Donkey anti-Rat | Invitrogen A21209 | Alexa Fluor 594 | 1:100 |
| Fig 6C & 6F | Anti-Mouse PRCP | Goat | Custom made (Anti-TND20 antibody (Ref #33) | 1:50 (brain) to 1:100 (kidney) | Donkey anti-Goat | Invitrogen A11055 | Alexa Fluor 488 | 1:100 (brain) and 1:200 (kidney) |

| | | | | | | | | |
|---|---|---|---|---|---|---|---|---|
| Sup Fig. S1A & S1B | Anti Human HK D5 | Rabbit | Custom made (Anti-LDDD... antibody | 1 μg/ml | Donkey anti-Rabbit | Jackson Immuno Res 711-035-152 | HRP | 1:5000 |
| Sup Fig S3 | Anti-Human HK D6 | Goat | Custom made (Anti-Cyst-AHDD) | 1:1000 | Donkey anti-Goat | Jackson Immuno Res 705-035-147 | HRP | 1:5000 |
| Sup Fig. S4A | Anti-Mouse HK D6 | Rabbit | Custom made (Anti-AHDD...) | 1 μg/ml | Goat anti-Rabbit | Licor 926-32211 | IRDye 800CW | 1:10000 |

**Supplemental Table S2**: Hematologic and Coagulation Parameters in Uninfected and *PbA*-infected C57BL/6 Mice

| Assay | Number | Uninfected C57BL/6J Mice[1] | *PbA*-Infected C57BL/6J Mice[1] | P value |
|---|---|---|---|---|
| WBC X $10^3$/l | 12 | 10 ± 2.6 | 2.9 ± 0.71 | P < 0.0001 |
| RBC X $10^{12}$/l | 12 | 10 ± 035 | 8.3 ± 1.5 | ns[2] |
| Hgb (g/dl) | 12 | 11 ± 0.67 | 9.9 ± 1.6 | ns |
| Hct (%) | 12 | 55 ± 1.6 | 40 ± 6.9 | P < 0.0001 |
| MCV (fl) | 12 | 55 ± 2.0 | 49 ± 1.0 | P < 0.0001 |
| MCH (pg) | 12 | 11 ± 0.5 | 12 ± 0.76 | ns |
| MCHC (g/dl) | 12 | 21 ± 1.4 | 25 ± 1.1 | P = 0.0021 |
| RDW (%) | 12 | 17 ± 0.91 | 16 ± 0.81 | ns |
| MPV (fl) | 12 | 4.9 ± 0.16 | 4.9 ± 0.48 | ns |
| Neutrophil (%) | 12 | 17 ± 3.3 | 26 ± 4.5 | ns |
| Lymphocyte (%) | 12 | 72 ± 4.8 | 55 ± 4.4 | P < 0.0001 |
| Monocyte (%) | 12 | 5.8 ± 1.2 | 15 ± 2.5 | ns |
| Eosinophil (%) | 12 | 3.4 ± 1.2 | 3.0 ± 1.3 | ns |
| Basophil (%) | 12 | 1.0 ± 0.47 | 1.0 ± 0.6 | ns |
| Platelet Count X $10^3/\mu$l | 10, 12 | 1413 ± 211 | 314 ± 123 | P < 0.0001 |
| aPTT (sec) | 20 | 31 ± 7.3 | 44 ± 8.8 | P = 0.0005 |
| PT (sec) | 20 | 11.7 ± 6.2 | 28 ± 17 | P = 0.008 |

[1] All values are mean ± 1 SD.

[2] ns = non-significant

**Supplemental Table S3**: Prekallikrein, Factor XII, and C1 Inhibitor levels in Uninfected and *PbA*-infected C57BL/6J Mice

| Assay | Number[2] | Uninfected C57BL/6J Mice | Infected C57BL/6J Mice | P value |
|---|---|---|---|---|
| Prekallikrein Activity[1] | 11,12 | 1.2 ± 0.28 | 0.86 ± 0.24 | P = 0.0002 |
| Prekallikrein Antigen * | 6, 8 | 1.0 ± 0.1 | 0.45± 0.1 | P = 0.0001 |
| Factor XII Coagulant Activity * | 8, 9 | 1.3 ± 0.09 | 1.4 ± 0.11 | P = 0.34 |
| Factor XII Antigen | 8,9 | 1.0 ± 0.1 | 1.4 ± 0.44 | P = 0.024 |
| Factor XI Coagulant Activity | 8,9 | 1.0 ± 0.16 | 1.0 ± 0.16 | P = 0.8 |
| C1 Inhibitor Activity * | 8,8 | 1.1 ± 0.19 | 1.6 ± 0.2 | P < 0.0001 |
| C1 Inhibitor Antigen* | 8, 8 | 1.0 ± 0.16 | 3.4 ± 0.71 | P < 0.0001 |

[1] All assay results are in IU/ml, which is the amount of protein in 1 ml plasma. The values are mean ± 1 SD

[2] Number of uninfected and infected samples, respectively

**Supplemental Table S4**: Summary of *PbA*-induced Infection in C57/BL/6J mice with Genetic Deletion of KKS & Certain Pharmacologic Inhibitors

| Genetics or Inhibitor | Protein | Parasitemia | SHIRPA Score* | Evans Blue** | Survival (Mean Day of Death)*** |
|---|---|---|---|---|---|
| *Kng1*$^{-/-}$ | Kininogen | 8% | P =0.0008 | P = 0.0006 | P = 0.0016 (7) |
| *Bdkrb1*$^{-/-}$ */Bdkrb2*$^{-/-}$ | Combined B1R & B2R KO | 17% | P =0.0006 | P = 0.0003 | P < 0.0001 (8) |
| *Bdkrb2*$^{-/-}$ | B2R | 13% | P < 0.0001 | P = 0.0012 | P = 0.0055 (7) |
| *Bdkrb1*$^{-/-}$ | B1R | 8% | P < 0.012 | P < 0.045 | P = 0.002 (7) |
| *Klkb1*$^{-/-}$ | Prekallikrein | 11% | P =0.003 | P <0.0001 | P < 0.0001 (7) |
| RZLT7824 | Plasma Kallikrein Inhibitor | 16% | P <0.01 | P < 0.008 | P = 0.0005 (7) |
| *F12*$^{-/-}$ | Factor 12 | 12% | NS | P < 0.03 | P < 0.0001 (7) |
| *Prcp*$^{gt/gt}$ | Prolylcarboxypeptidase | 12% | P <0.0067 | P < 0.0007 | P < 0.0003 (8.5) |
| PrCP Inhibitor | Prolylcarboxypeptidase Inhibitor | 10% | P = 0.0095 | P < 0.039 | - |
| *Prcp*$^{fl/fl}$ *Cre* | PRCP Conditional KO Brain Endothelium | 14% | P < 0.001 | P < 0.003 | - |

* Values comparing infected wild-type mice to the genetic deletion animal or pharmacologic inhibitor are presented as the degree of significant change between the experimental mice and their simultaneous controls. For SHIRPA Score, the P value is the significance of the degree of reduction from neurologic and behavior deterioration.

**For Evans Blue studies, the P value indicates the significance of the degree of reduction of Evans Blue dye uptake into the brain.

***For Survival, the P value represents the significance of the increase in survival of the experimental animal. The number in parenthesis represents the median day of survival. Note: infected wild-type C57BL/6J mice live 6 days without treatment.

**Supplemental Table S5**. Antibodies to High Molecular Weight Kininogen used in this Investigation

| Antibody | Species | Abbreviation | Sequence | Domain | Figures |
|---|---|---|---|---|---|
| Anti-human HK | goat | | Purified HK | | Fig 1A |
| Anti-human HK | rabbit | Anti-LDDD… | LDDDLEHQGGHVLDHGHKHKHGHGHGKHKNKGKKNGK | D5 | Fig 1C, S1A, S1B |
| Anti-mouse HK | rabbit | Anti-CysAHD… | anti-Cys-AHDDLIPDIHVQPDSLSFKLISDFPEAT | D6 | Fig 2C, Fig 2D & Suppl. Fig. S3 |
| Anti-mouse HK | rabbit | Anti-AHD... | AHDDLIPDIHVQPDSLSFKLISDFPEATSPK | D6 | Suppl Fig S4 |

**Legends for Movie Videos**

**Movies S1**, **S2**, and **S3** should be watched simultaneously.

**Movie S1** are infected wild-type (C57BL/6J) control mice with *P. berghei* ANKA (*PbA*) infection on day 5 with cerebral malaria. The earliest neurologic signs of CNS disease in these animals are their sitting up on their rear legs and the repetitive motion of the front paws.

**Movie S2** shows *PbA*-infected *Kng1*$^{-/-}$ (total kininogen null) mice on day 5 with cerebral malaria at the same time as the wild-type control mice in Movie S1.

**Movie S3** show *PbA*-infected combined *Bdkrb1*$^{-/-}$/*Bdkrb2-/-* mice (bradykinin B1 and B2 receptor knockouts) on day 5 with cerebral malaria at the same time as the wild-type control mice in Movie S1

**Movies S4**, **S5**, and **S6** should be watched simultaneously

**Movie S4** shows *PbA*-infected C57BL/6J wild-type mice on day 5 with cerebral malaria. It is the simultaneous infected control mice for Movie S5 and S6. Four mice are severely impaired with disease and 1 mouse has early neurologic signs.

**Movie S5** are *PbA*-infected *Klkb1*$^{-/-}$ mice on day 5 with cerebral malaria. The video was taken at the same time as Movie S4. Four mice appear normal; one mouse in the lower right is impaired.

**Movie S6** are *PbA*-infected C57BL/6J wild-type mice on day 5 treated with the plasma kallikrein inhibitor RZLT7824 for 2 days prior. Most animals are moving well part of the time. The video was taken at the same time as Movie S4.

**Movie S7**: Wild-type mice with *PbA*-infection on day 6. One group got artesunate therapy alone on day 5 (top video); The other group got artesunate therapy along with the plasma kallikrein inhibitor RZLT7824 on day 5 at the same time (bottom video). The videos of the surviving mice were taken on day 6, 24 h after starting treatment. Differences in neurologic behavior were observed within 24 h between the two groups of mice.
