# Supplementary material for: Bradykinin contributes to vasogenic edema in murine experimental cerebral malaria
Source: J Clin Invest. 2026 Jul 14;136(17):e202285. doi: 10.1172/JCI202285 (PMC13528921; doi:10.1172/JCI202285)

Unedited Figure 1A

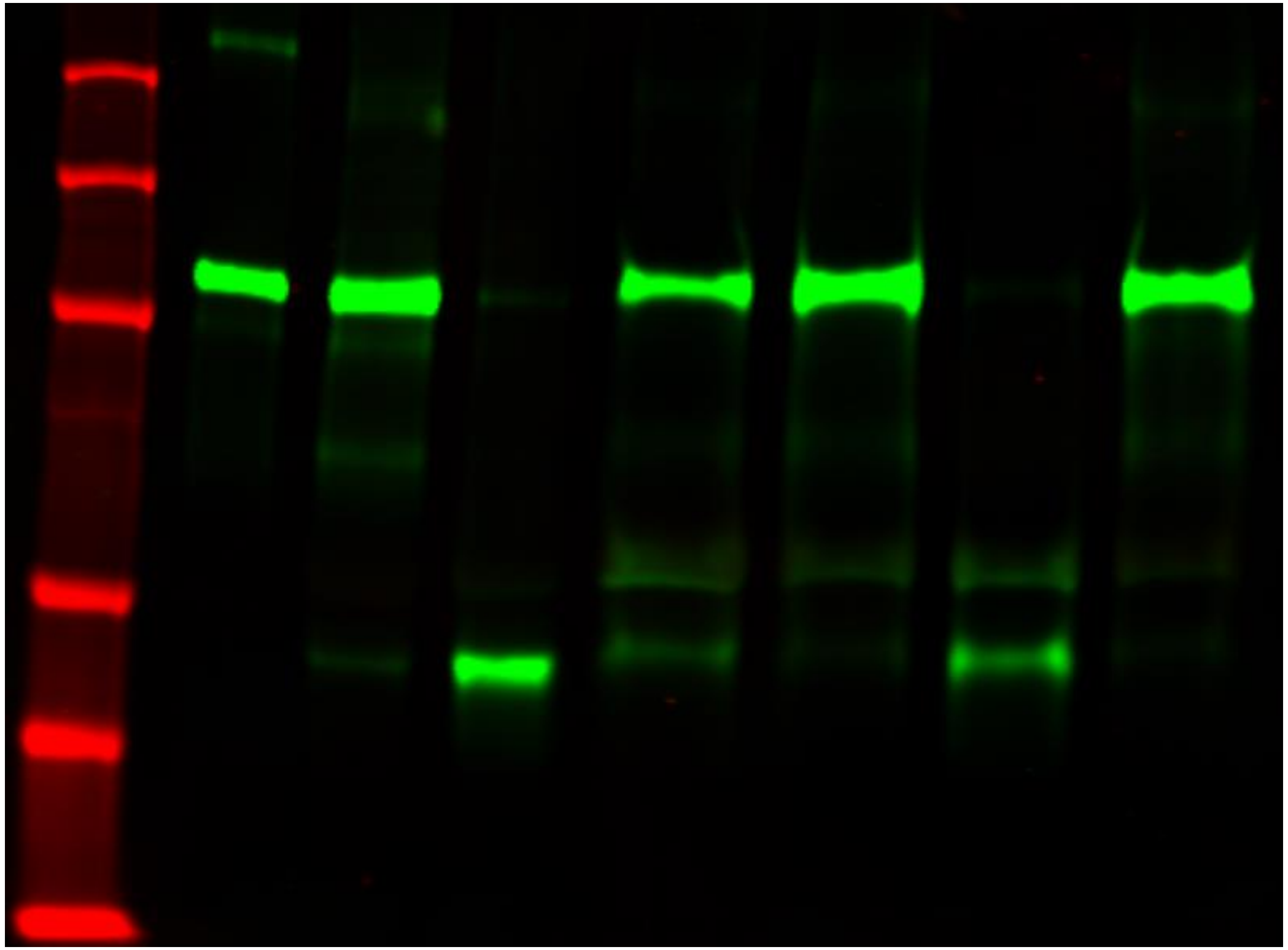

Unedited Figure 1B

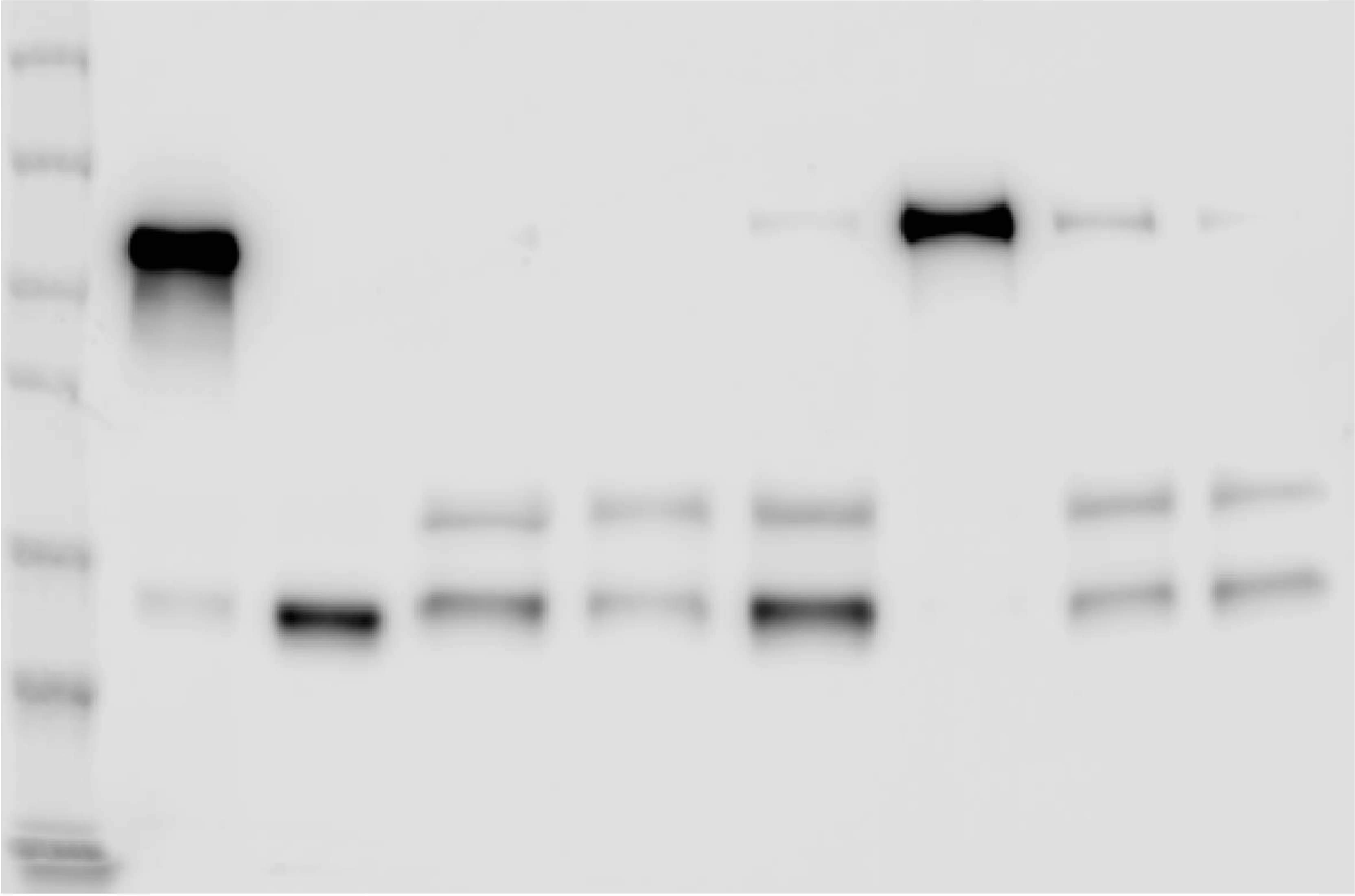

Unedited Figure 2D

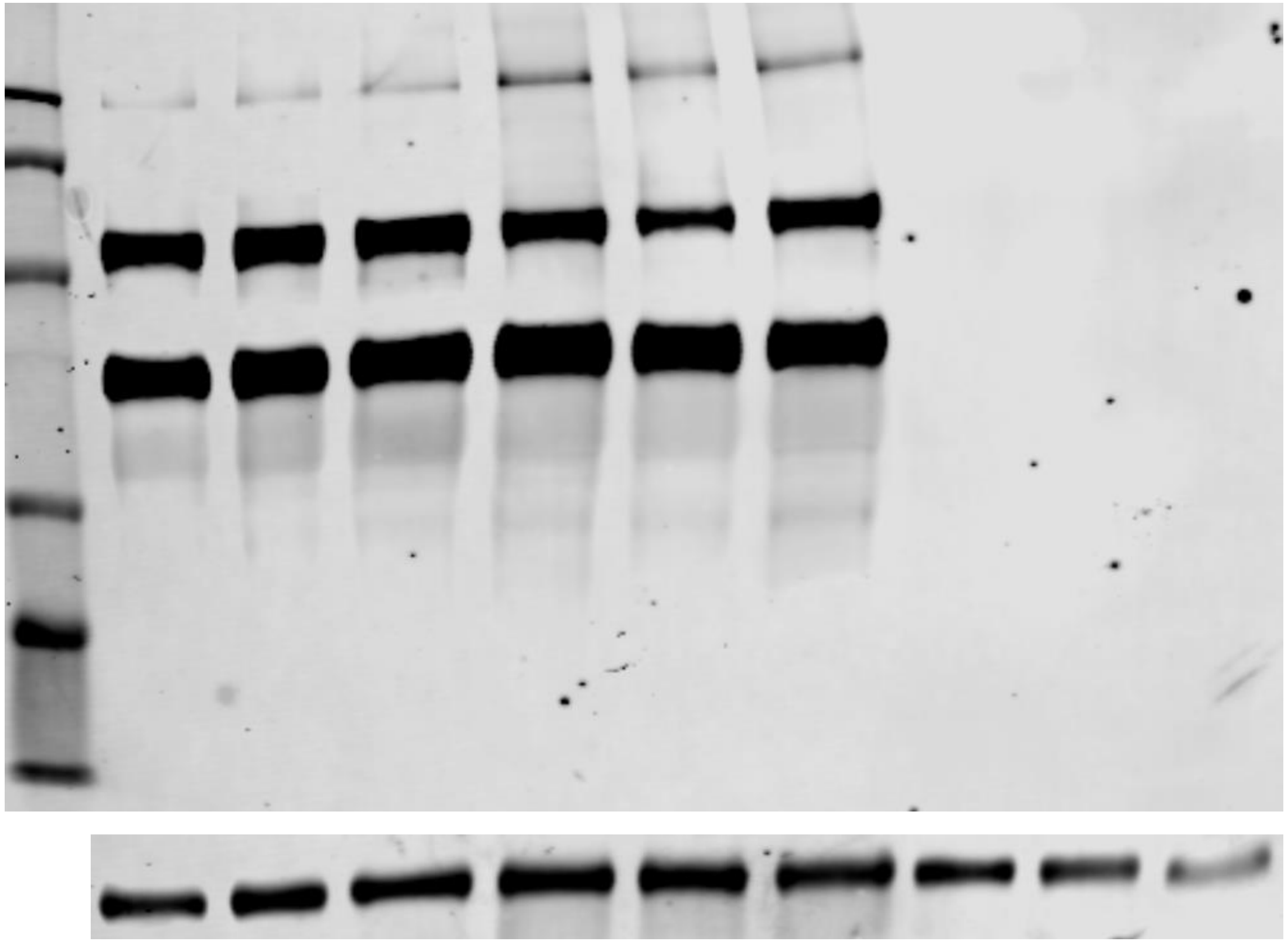

← Cropped transferrin blot (see next panel)

Unedited Figure 2B Transferrin blot

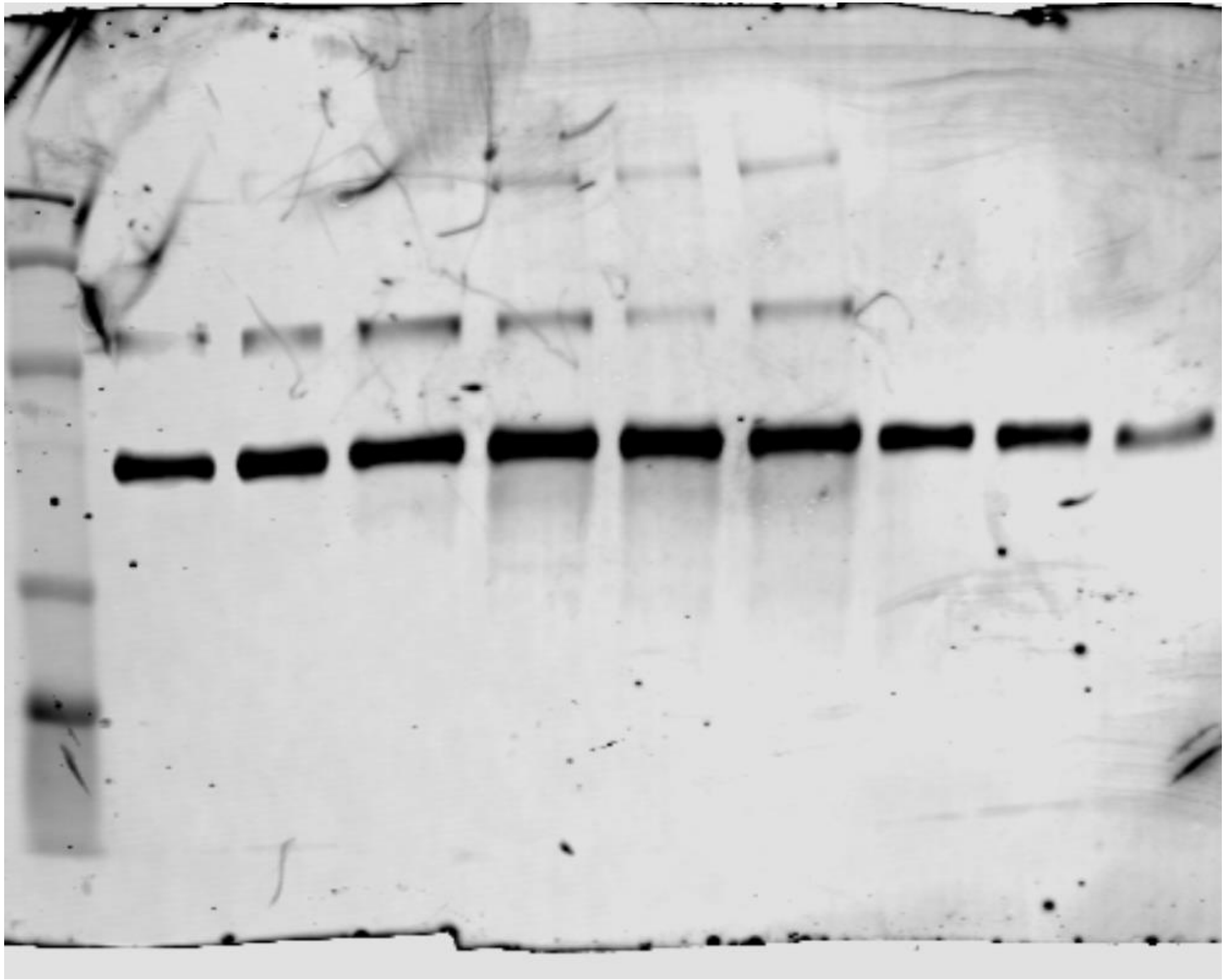

Unedited Figure 6B

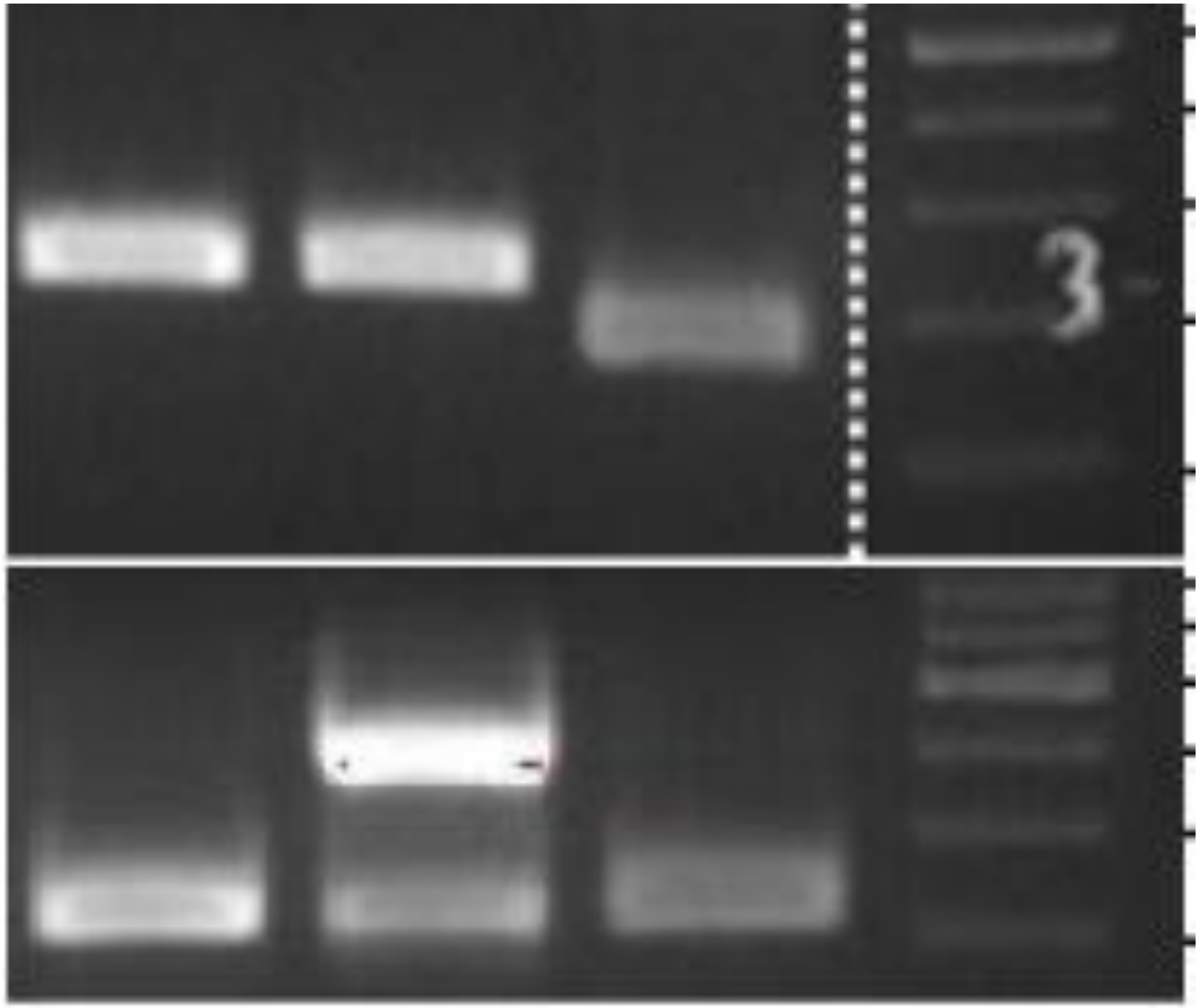

Supplement Figure S3 for HK

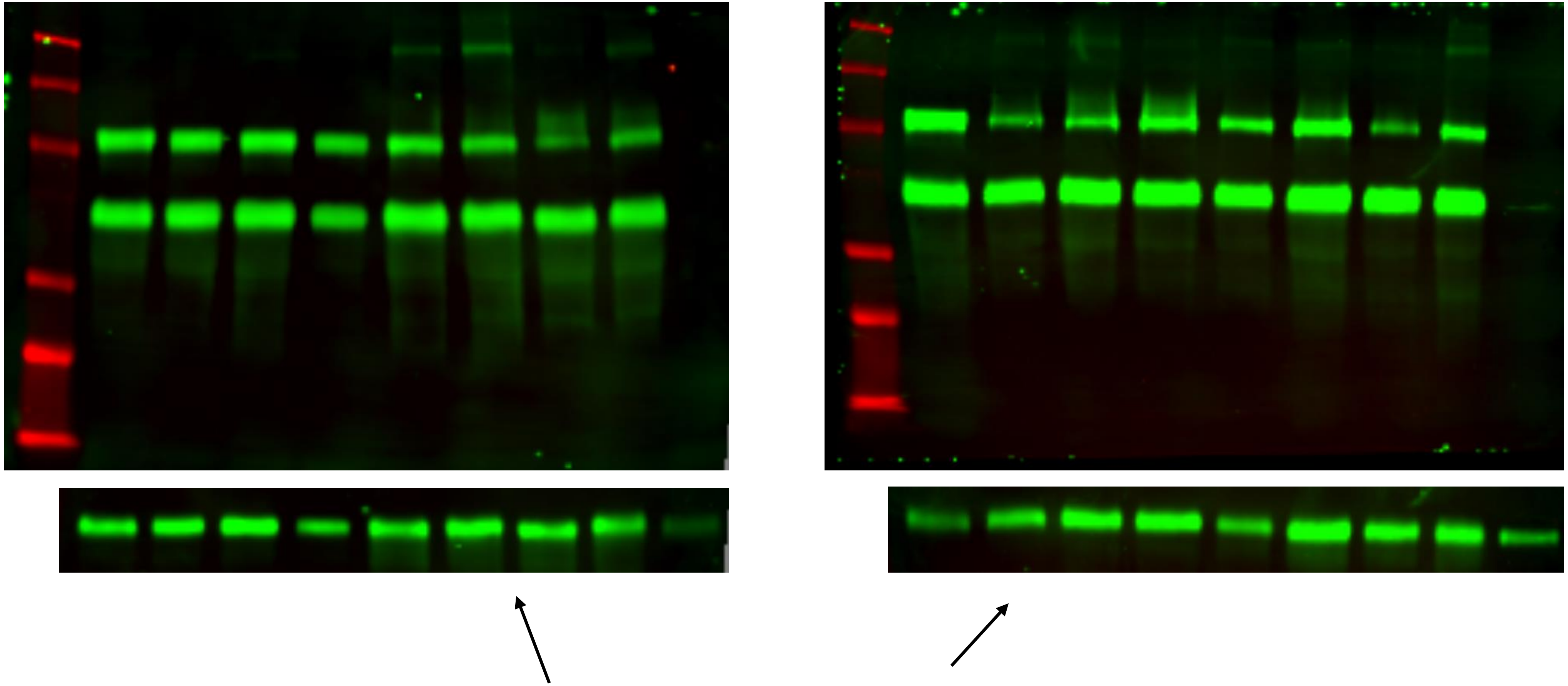

Cropped Transferrin Blot Loading Controls
(See next panel)

Supplement Figure S3 –-2 Transferrin blots for mouse HK

Transferrin Loading Control

A

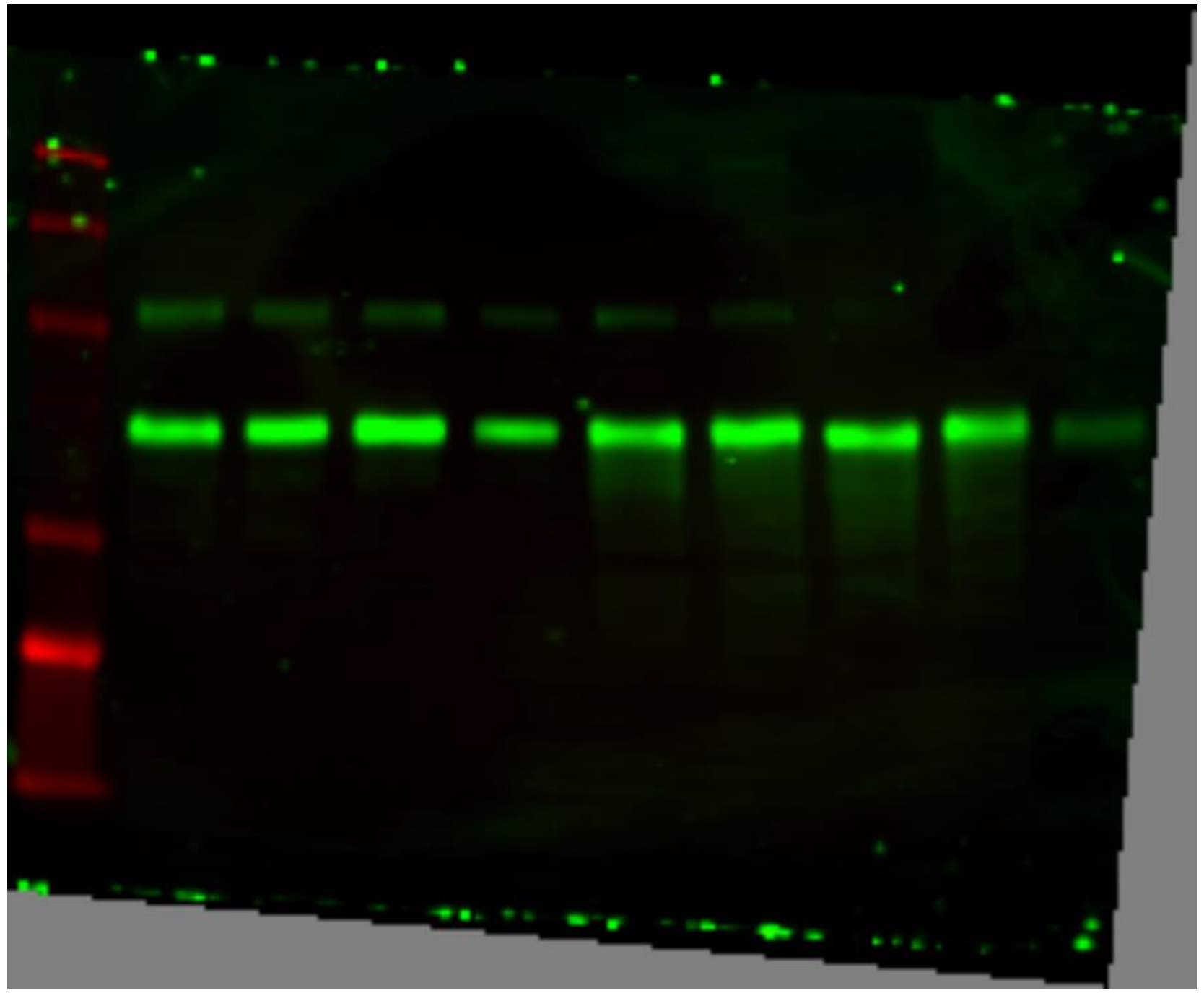

Transferrin Loading Control

B

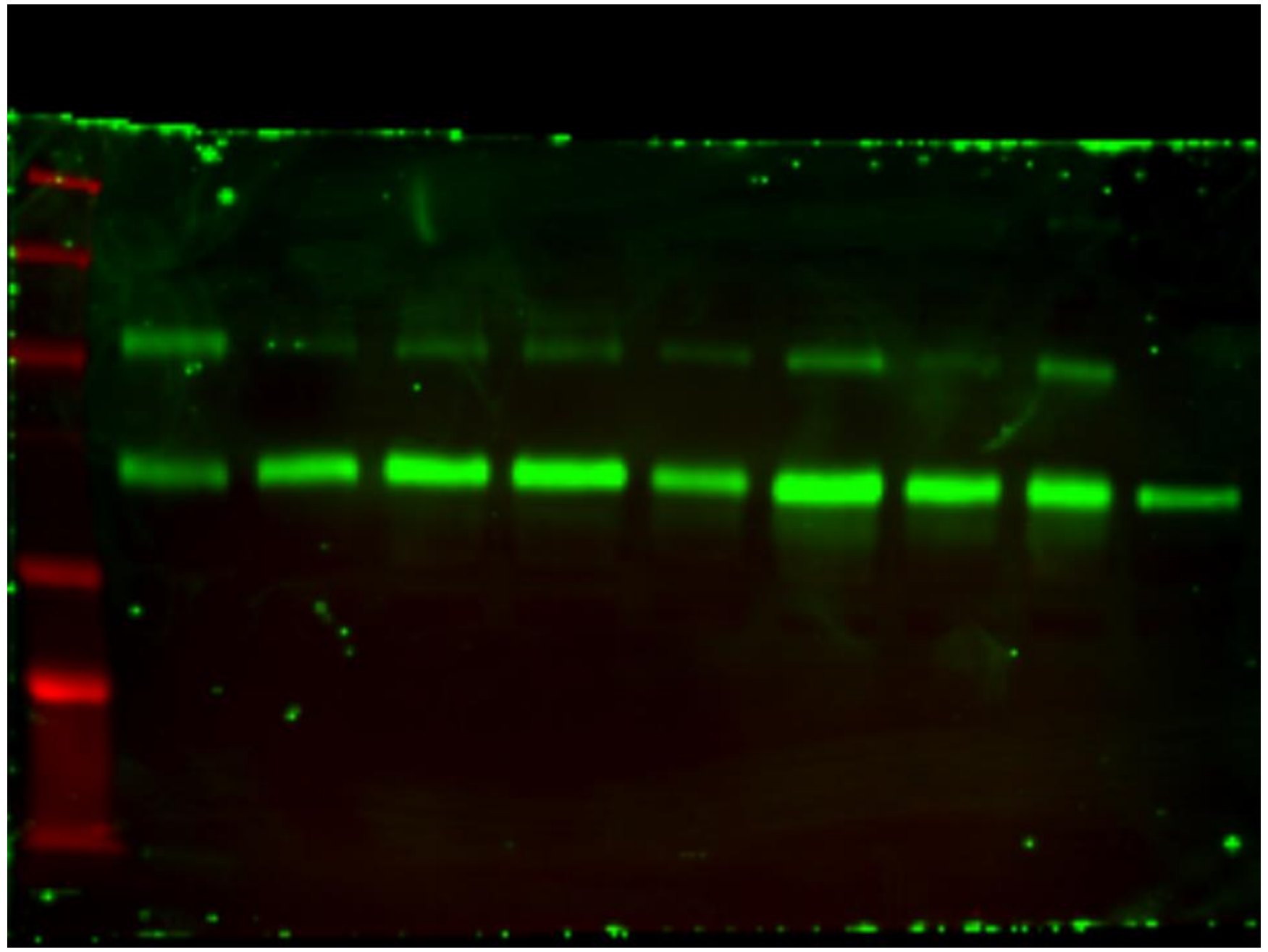

Supplement Figure S4 for HK

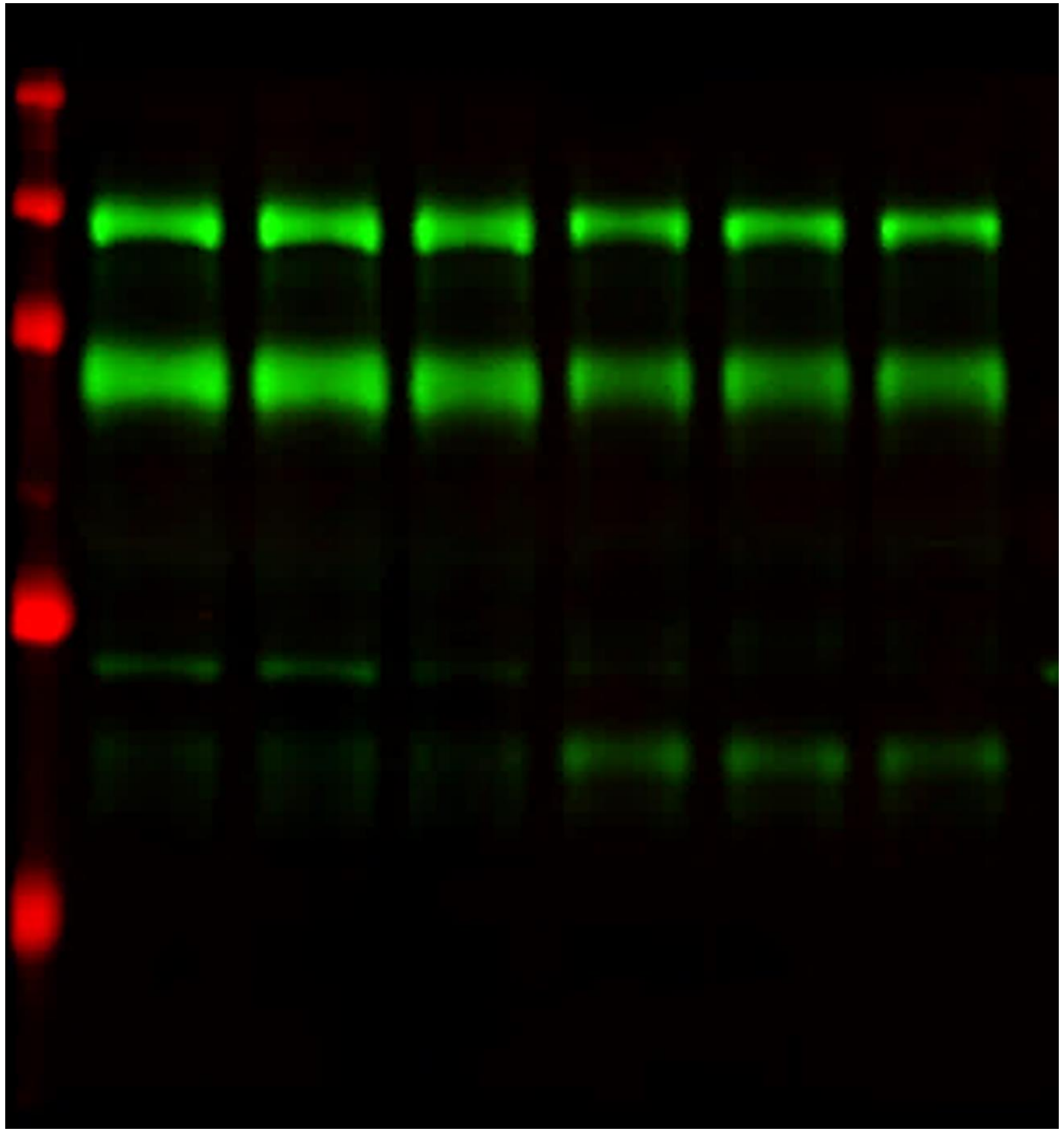

Supplement Figure S5 for Factor XII Quantification

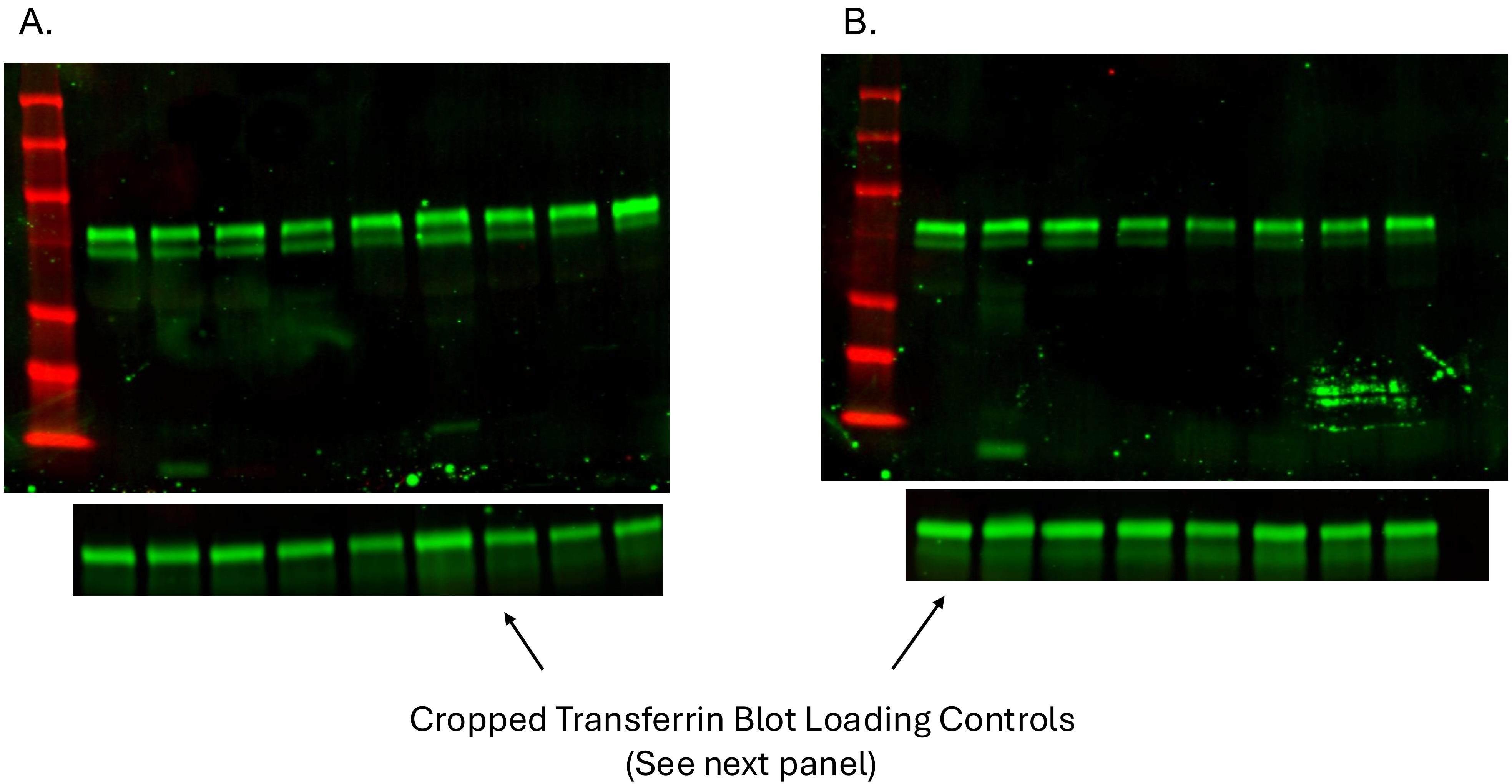

Supplement Figure S5 - Transferrin blots for mouse FXII

A Transferrin Loading Control

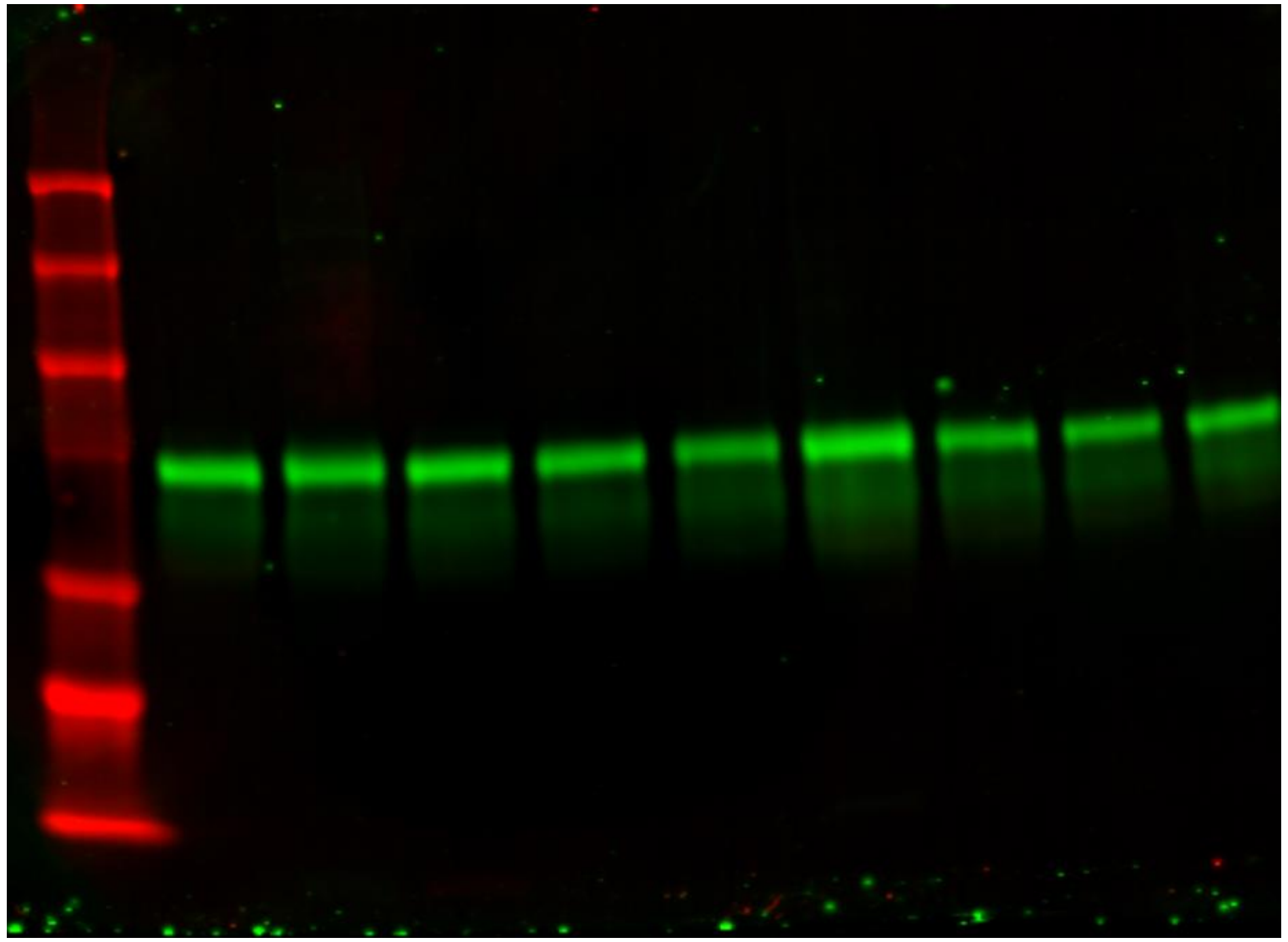

B Transferrin Loading Control

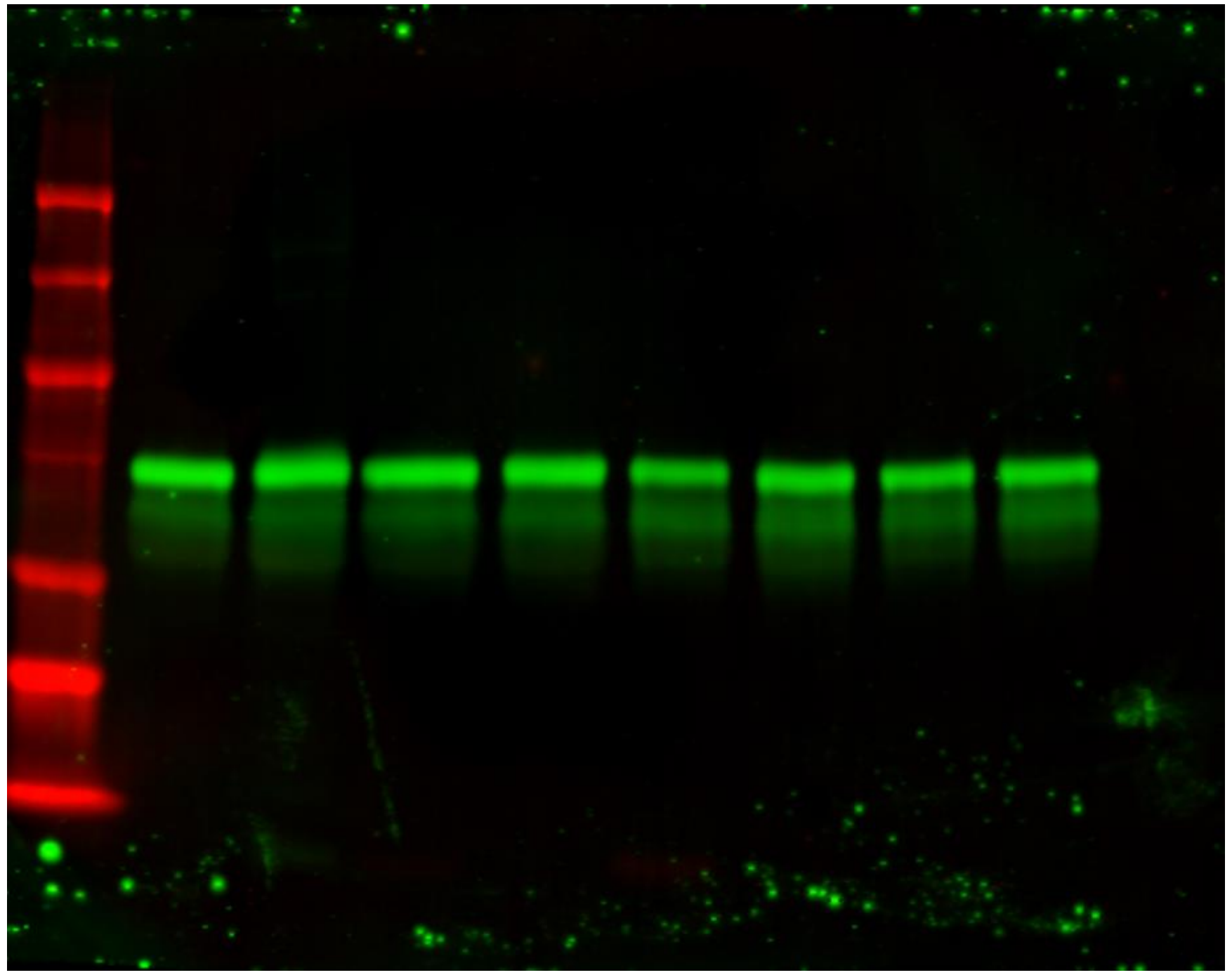

Supplement Figure S6 for PK Quantification

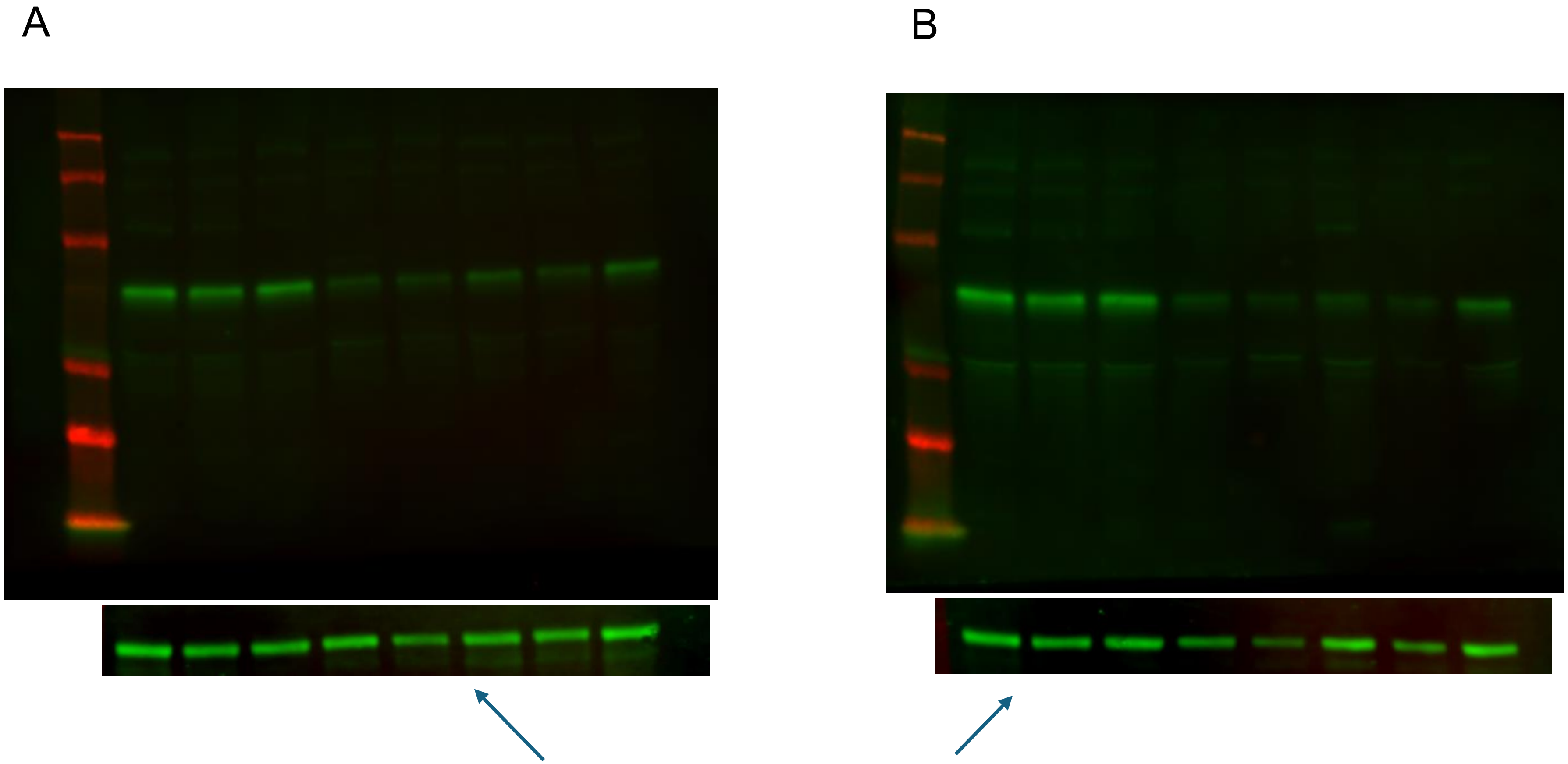

Cropped Transferrin Blot Loading Controls
(See next panel)

Supplement Figure S6 - Transferrin blots for mouse PK

A Transferrin Loading Control

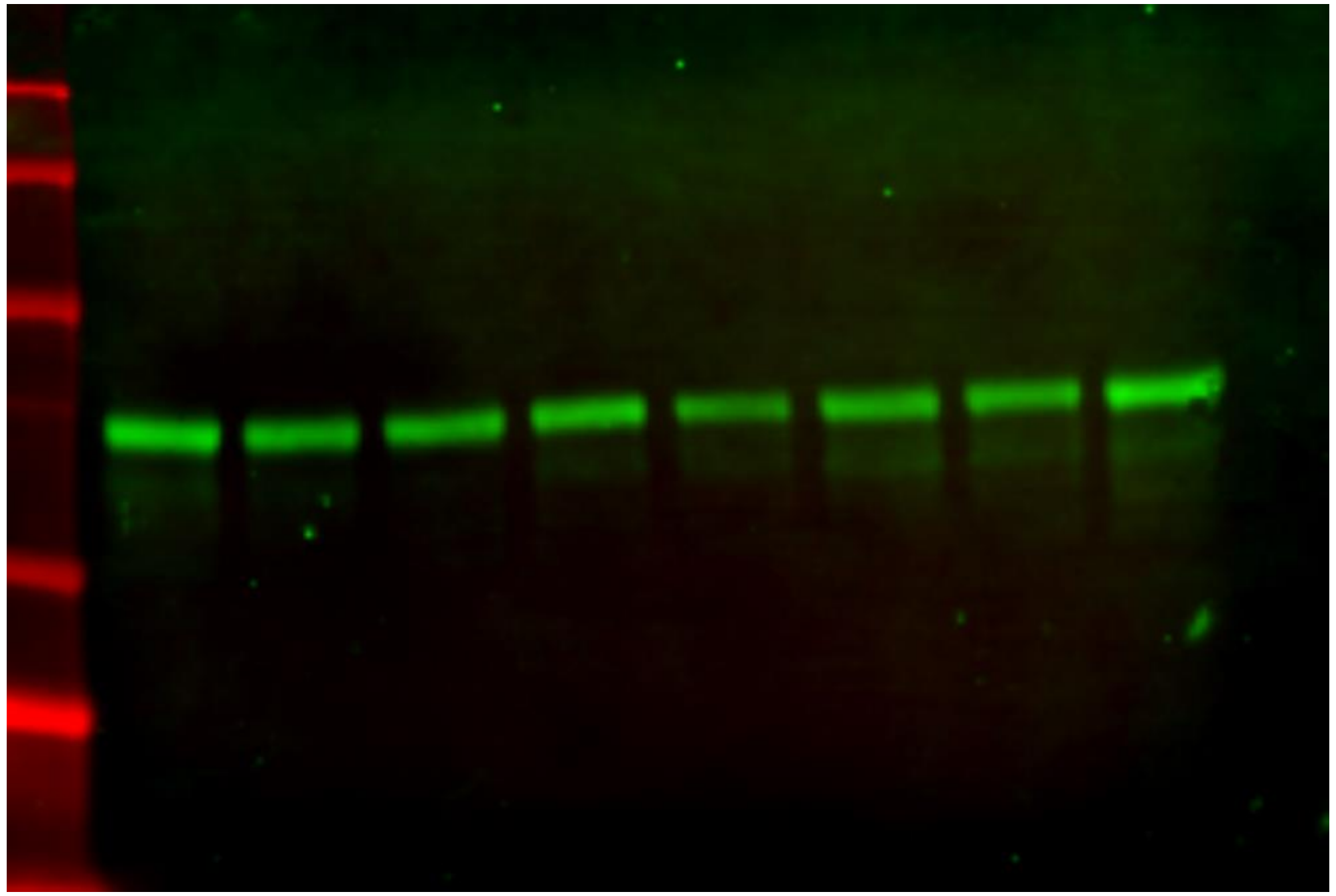

B Transferrin Loading Control

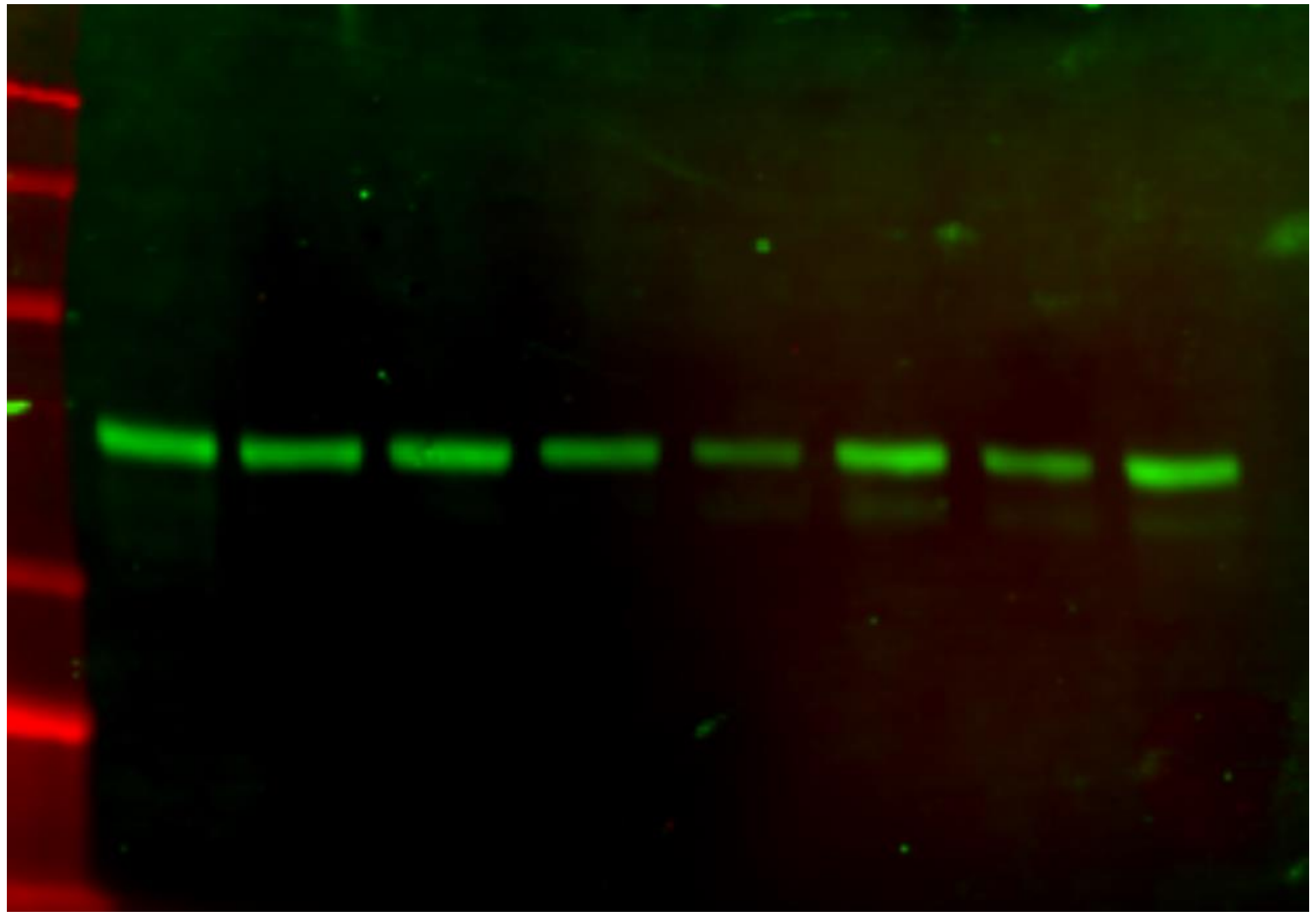

Supplement Figure S7 for C1INH Quantification

A

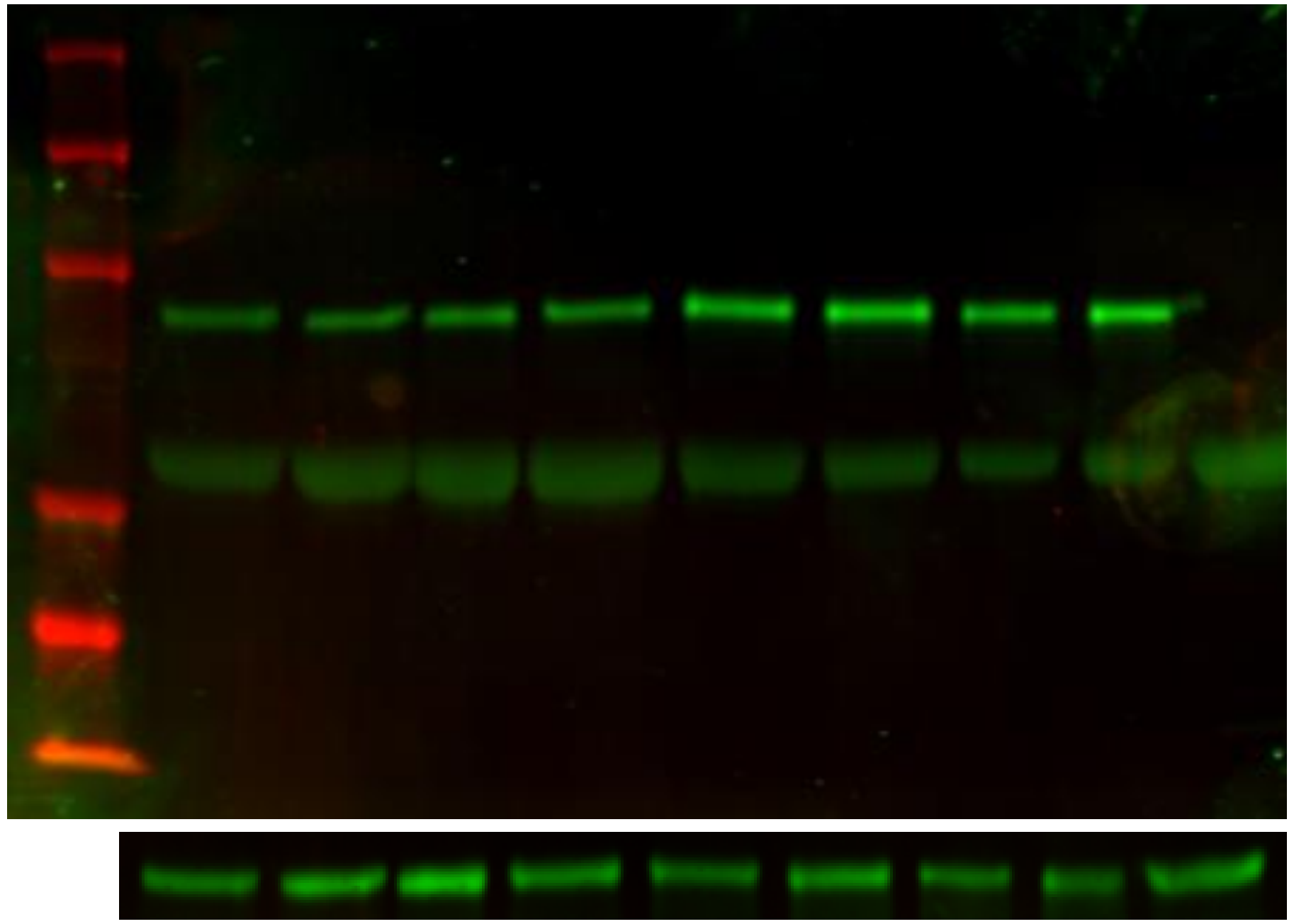

B

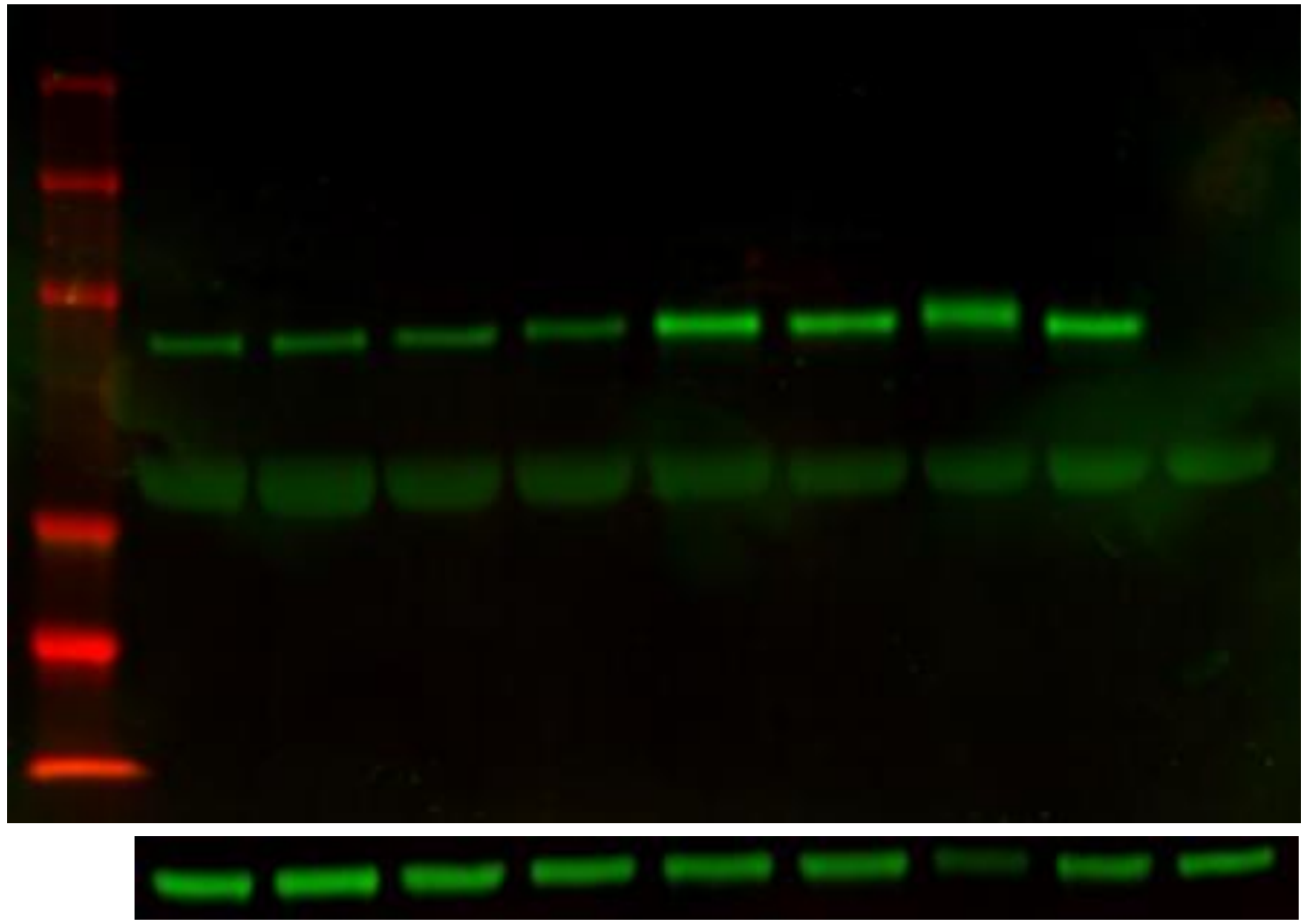

Cropped Transferrin Blot Loading Controls
(See next panel)

Supplement Figure S7 Transferrin blots for mouse C1INH

A Transferrin Loading Control

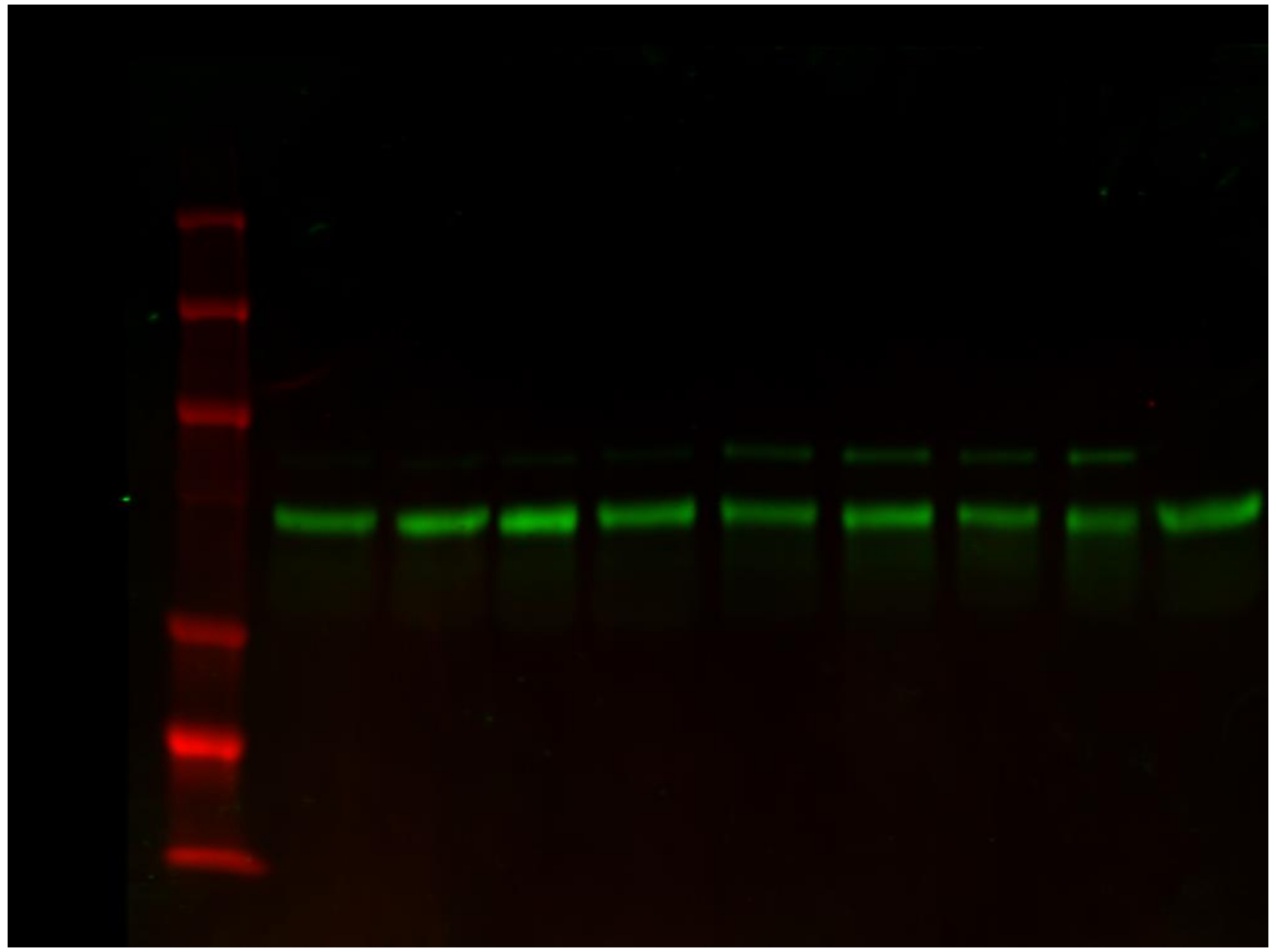

B Transferrin Loading Control

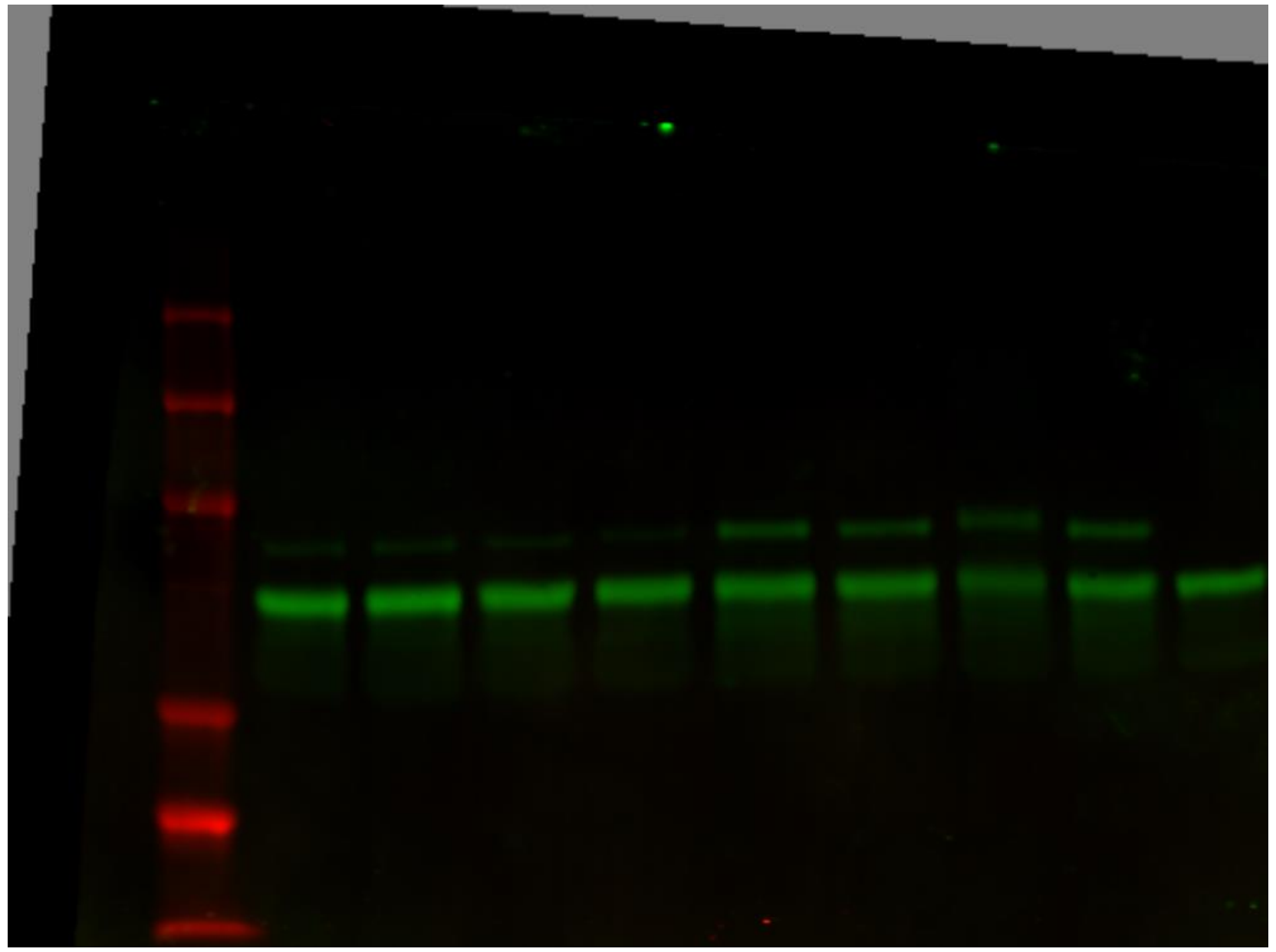

Supplement: Supplementary file 2 [file jci-136-202285-s160.pdf]
